# Archaeal Viruses, Not Archaeal Phages: An Archaeological Dig

**DOI:** 10.1155/2013/251245

**Published:** 2013-04-07

**Authors:** Stephen T. Abedon, Kelly L. Murray

**Affiliations:** Department of Microbiology, The Ohio State University, 1680 University Drive, Mansfield, OH 44906, USA

## Abstract

Viruses infect members of domains *Bacteria*, *Eukarya*, and *Archaea*. While those infecting domain *Eukarya* are nearly universally described as “Viruses”, those of domain *Bacteria*, to a substantial extent, instead are called “Bacteriophages,” or “Phages.” Should the viruses of domain *Archaea* therefore be dubbed “Archaeal phages,” “Archaeal viruses,” or some other construct? Here we provide documentation of published, general descriptors of the viruses of domain *Archaea*. Though at first the term “Phage” or equivalent was used almost exclusively in the archaeal virus literature, there has been a nearly 30-year trend away from this usage, with some persistence of “Phage” to describe “Head-and-tail” archaeal viruses, “Halophage” to describe viruses of halophilic *Archaea*, use of “Prophage” rather than “Provirus,” and so forth. We speculate on the root of the early 1980's transition from “Phage” to “Virus” to describe these infectious agents, consider the timing of introduction of “Archaeal virus” (which can be viewed as analogous to “Bacterial virus”), identify numerous proposed alternatives to “Archaeal virus,” and also provide discussion of the general merits of the term, “Phage.” Altogether we identify in excess of one dozen variations on how the viruses of domain *Archaea* are described, and document the timing of both their introduction and use.

## 1. Introduction

…most viruses infecting archaea have nothing in common with those infecting bacteria, although they are still considered as “bacteriophages” by many virologists, just because archaea and bacteria are both prokaryotes (without nucleus). [1]

For historical reasons, bacteriophage is widely used to refer to viruses of bacteria (and sometimes even archaea). The problem with such nomenclature is that it artificially divides the virosphere into two camps, with viruses of bacteria and archaea on one hand and viruses of eukaryotes on the other. [2]

Viruses are infectious agents that alternate between autonomous, encapsidated states known as virions, which are “packages of genes” [3], and unencapsidated, intracellular states known as infections [1], infected cells [3] or, more holistically, as “Virocells” or “Ribovirocells” [4, 5]. Numerous differences exist among viruses in terms of virion morphology, genome architecture, and infection strategy [6], and viruses also may be differentiated as a function of host range [7]. While it is possible to describe a virus's host range in terms of what species or even subspecies or strains of cellular hosts it is capable of infecting, it is also possible to distinguish between susceptible hosts more broadly. For example, one can, though with some ambiguity, distinguish between those hosts that are macroscopic versus those that instead are microscopic, with the latter hosting what can be described as viruses of microorganisms, or VoMs [8].

Among the viruses of microorganisms are those that infect microscopic eukaryotes along with those that instead infect prokaryotes. While the viruses of eukaryotes nearly exclusively are described as just that, that is, as viruses, the viruses of prokaryotes have been burdened with a more complicated naming history. Here we look at the naming conventions that have been applied to the latter, with some emphasis on considering the relative merits of the term “Phage” as a descriptor particularly of the viruses of domain *Archaea*. We agree with what we observe to be a near consensus within the field that the use of the term “Virus” as well as the qualification “Archae-” or “Archaeal”—as in, for instance, “Archaeal virus”—is both logical and reasonable, echoing, for example, the fairly common usage of “Bacterial virus” as an alternative to “Bacteriophage” or “Phage.” For approximately half of the 40 or so years that these viruses have been studied, however, the explicit phrase “Archaeal virus” did not exist in the published literature. The absence of this phrase prior to the early 1990s reflects the replacement only in 1990 of “Archaebacteria” [9] with “*Archaea*” [10] as a descriptor of this cellular group.

**Figure 1:**
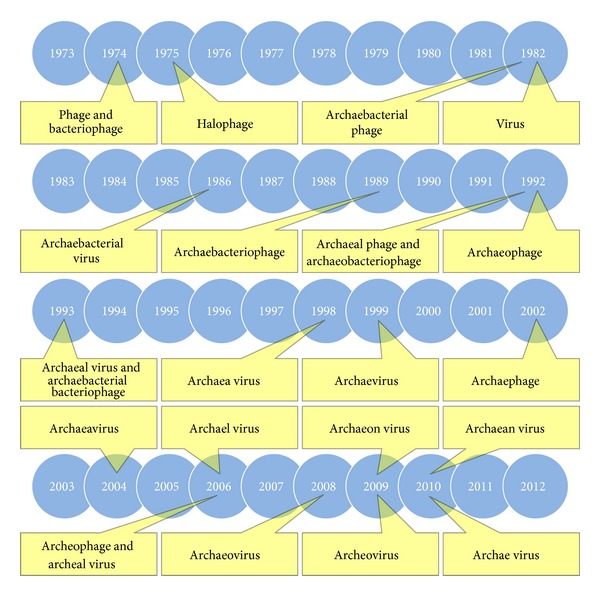
Various names that have been used to generally describe archaeal viruses as well as subsets of those viruses (e.g., “Halophage”). A sampling of those terms along with what to the best of our knowledge are their dates of introduction into the literature are presented in a timeline format. Note in particular the diversity as well as, even over the last decade, an apparently ongoing lack of consensus. References for first use, again to the best of our knowledge, are as follows: Bacteriophage (and Phage) [11], Halophage [12], Archaebacterial phage [13], Virus [13] (see, however, also [14]), Archaebacterial virus [15], Archaebacteriophage [16], Archaeobacteriophage [17], Archaeal phage [18], Archaeophage [19], Archaeal virus [20], Archaebacterial bacteriophage [21], Archaea virus [22], Archaevirus [23], Archaephage [24], Archaeavirus [25], Archael virus [26] (also found as a typo in a 1999 publication [27]), Archeal virus [28], Archeophage [29], Archaeovirus [30, 31], Archeovirus [32], Archaeon virus [33], Archae virus [34], and Archaean virus [35].

Here we consider the history of the general naming of archaeal viruses as found within the published literature. In addition to exploring the timing of the introduction of the term “Archaeal virus,” we also consider the transition from “Phage” to “Virus” as seen approximately a decade earlier as well as the use of various related terms including “Archaebacteria,” “Halophage,” and “Prophage.” A summary of some of the terms that have been used to describe archaeal viruses, along with what we have been able to ascertain are their dates of introduction into the literature, is presented in Figure 1. Overall use in the literature—particularly as seen in journal articles—of “Virus” and “Phage” (including variations on these terms) as well as “Prophage” and “Halophage”, is recorded in Table 1 (see Supplementary Materials available online at http://dx.doi.org/10.1155/2013/251245) as well as Table 2 (supplementary materials).

## 2. Kingdoms, Urkingdoms, Empires, Superkingdoms, and Domains

While schemes of organism classification have existed for millennia, prokaryotes were explicitly introduced into such systems only in 1938, by Copeland [36]. This “new” kingdom was dubbed the familiar Monera, as also used by Whittaker, in 1969, as part of his well-known five-kingdom system of organism classification [37]. Beyond Copeland's four-kingdom system and Whittaker's five-kingdom system, a modification was suggested in which cellular organisms were distinguished at a higher level than that of a kingdom, into simply prokaryotes versus eukaryotes [38], which can be designated as empires or superkingdoms [10, 39], that is, Prokaryota(e) versus Eukaryota(e). At that time, viruses similarly were distinguished, at least semantically, into phages (the viruses of prokaryotes) and viruses for everything else. For discussions of the early history of phages along with viruses more generally, see [3, 8, 40, 41] along with references cited.

As is well known especially to microbiologists, the five-kingdom system has given way to the three-domain system [10]. There the prokaryotes of kingdom *Monera* were, rightly or wrongly [42, 43], further differentiated into the domains *Bacteria* and *Archaea* versus the eukaryotes, with the latter dubbed domain *Eucarya* (or *Eukarya*) [44]. Note that the domain that would be named *Archaea* in 1990 [10] was first designated, in 1977, as “urkingdom… *archaebacteria*” by Woese and Fox [9]. From page 5089 of that publication (emphasis is theirs):

The apparent antiquity of the methanogenic phenotype plus the fact that it seems well suited to the type of environment presumed to exist on earth 3-4 billion years ago lead us tentatively to name this urkingdom the *archaebacteria*.

In terms of the viruses associated with each of these domains, an obvious question then was whether those infecting members of domain *Archaea* should retain the “Phage” designation or instead assume the more general term of “Virus.” Here we consider the history of this issue especially in terms of published usage, focusing particularly on the relatively long transition from “Bacteriophage” to “Archaeal virus.” We additionally consider overall usage as determined by examination of individual publications.

## 3. Methods

Myriad approaches were used to identify archaeal virus-associated publications. Where possible, publications were obtained, in various forms, and if not already searchable as PDFs (Portable Document Format) then digitally scanned and/or subject to optical character recognition (OCR). Articles were then computer searched using the Adobe Acrobat search function. This was initially done in bulk but ultimately individually, for various terms, where the context therefore could be observed. Terms found within reference lists were ignored and terms such as “Phage” that did not appear to be associated with considerations of archaeal viruses were also not considered (thus resulting in a “No” designation in Table 1, supplementary materials). Note that generally we equate “Virus” with “Viruses” as well as “Viral” but not with “Virion” and also consider the use of “Phage” as well as “Virus” both alone and as suffixes. We have also disregarded trivial misspellings such as “Archeal virus” as used in the same publication as “Archaeal virus.”

We avoid describing a publication as containing the term “Virus” if that term was limited to within the name of a virus, for example, “*Acidianus* bottle-shaped virus” [45]. We have not applied the same “rule” to virus names that contain “phage,” however, such as “*Methanobacterium* phage ΨM1”, since technically this could be described instead as, for example, “*Methanobacterium* archaeal virus ΨM1” or simply “*Methanobacterium* virus ΨM1”. The result of this bias is what we consider to be a conservative underestimation of the use of “Virus” along with an overestimation of use of “Phage.” In terms of consideration of use of “Phage” within the literature, we feel that this approach is reasonable particularly since a publication will still be described as containing “Virus” if it also uses “Virus” to describe archaeal viruses outside of the names of specific viruses. The example at the beginning of this paragraph, on the other hand, was designated as *not* containing “Virus” since the quoted text was the sole use of “Virus” to describe an archaeal virus that we were able to locate in the nonreferences portion of that publication. In any case, “Virus” as well as “Phage” must have been used to describe one or more archaeal viruses to be counted towards these tallies.

Our working assumption is that our reference list (supplementary materials) is less than fully complete. Explicitly missed are references that were not published in English as too patents. We also have likely missed various chapters along with whole books, especially those on subjects tangential to that of archaeal viruses. This is true also for materials that we were unable to obtain and therefore examine as well as any references that were not yet indexed nor easily identified prior to approximately January 1, 2013. Altogether, 694 references were identified, examined, and otherwise recorded as referring in some manner to archaeal viruses. All of these are listed in the reference section of the supplementary materials.

Just as human-generated indices can miss sought information, so too are digital searches fallible. It is possible also that relevant context was missed in the course of examining individual publications, though ultimately the decision as to whether a term such as “Phage” or “Virus” was used in a publication to refer to an archaeal virus, or not, was based on the experience of the lead author (S. T. Abedon). As with any “Experiment,” the resulting output—given limitations in technology, retrieval, and subjective judgment as well as simply “operator error”—should be viewed as representative, in this case of the archaeal virus literature, rather than either a complete or error-free sampling. 

## 4. The “Phage” to “Virus” Transition

The earliest references to viruses of domain *Archaea* described them as phages or bacteriophages. This usage was appropriate, especially at the point of initial discovery, as the classification of their hosts as distinct from bacteria was not yet appreciated; that is, and as noted above, the term “Archaebacteria” dates to 1977 [9] but the first such archaeal virus publication was that of Torsvik and Dundas in 1974 [11]. That paper, published in *Nature*, was titled “Bacteriophage of *Halobacterium salinarum*.”

The Torsvik and Dundas paper was followed by a publication during the next year, 1975, by Wais et al. [12]. This was also in *Nature* and was titled “Salt-dependent bacteriophage infecting *Halobacterium cutirubrum* and *H. halobium*.” In each case neither “Virus,” “Viral,” nor the root “Arch…” appear in the article. With some irony, however, the article immediately following Torsvik and Dundas describes bacteriophage PM2—presumably, though not specified, of *Pseudoalteromonas* of domain *Bacteria*—as both a “Virus” and “Viral” [46]. Equivalent use of “Phage” as well as lack of use of “Virus”, “Viral”, and “Arch…” can be seen also with Torsvik and Dundas in 1978 [47] and 1980 [48] as well as Pauling in 1982 [49]. Pauling, however, does extensively employ “Virion”, though we do not consider this term to be equivalent to “Virus” or “Viral” as phages too are routinely described as possessing virions, for example, bacteriophage virion(s) [50, 51].

Somewhat ambiguously, and in a report rather than an otherwise formal publication, Stube et al. [14] provide what to our knowledge is the first association of “Virus” with archaeal viruses. In their Table 2, which lists “Organisms of the Northern Arm of the Great Salt Lake,” the term “Halophages” as a “Scientific Name” is associated with “Bacterial viruses” as a “Common Name.” Later, on page 48, “Halophages” is then associated with “*Halobacterium halobium*” (see a footnote of Table 1, supplementary materials, for the full quotation of the latter). Of interest, for their Table 2, Stube et al. cite Post, 1975, in an article titled “Life in the Great Salt Lake” [52]. In that paper, reference is made only to “Bacterial viruses” (page 44) along with the statement (page 46) “Virus parasites of the bacteria also live in the lake.” The bacteria or at least “Halophilic bacteria” are indicated, however, as “*Halobacterium*—*Halococcus*” (page 44).

In 1982, in an article by Schnabel et al. [13] titled “*Halobacterium halobium* phage **ϕ**H,” the word “Virus” to our knowledge makes it first unambiguous as well as mainstream appearance in what would become the archaeal virus literature. This is found in the first sentence of the abstract: “Phage **ϕ**H, a novel virus of the archaebacterium *Halobacterium halobium*, resembles in size and morphology two other *Halobacterium* phages.” In addition, under keywords is found “archaebacteria/virus,” though otherwise “Phage” is used far more often than “Virus” in this publication. Furthermore, in two locations the term “Archaebacterial phage” can be found for the first time. This latter usage seems to reflect the citing of Fox et al. [53] as found in their Introduction (page 87).

The genus *Halobacterium* belongs to a group of organisms now known as archaebacteria which differ in many respects from both eucaryotes and eubacteria (Fox et al., 1980)… This paper presents the first analysis of the DNA of an archaebacterial phage.

By contrast, and notably, this publication by Fox et al. is not cited in Pauling's article of the same year [49], along with a lack of use of “Virus” or “Viral” other than in the reference section in that other 1982 publication. 

The Schnabel et al. [13] article represents something of a transition in usage towards the inclusion of “Virus” as a descriptor, though this could be viewed more as a matter of style rather than something that is particularly profound, as phages at this point in time (i.e., 1982) had been known to be viruses for decades (see, however, the immediately following paragraph). Certainly more than a matter simply of style, though, is the first use of “Archae…” as a qualifier for the type of virus under study. A third article from 1982, also from Schnabel and Zillig [54], splits the difference between these two other 1982 studies by citing Fox et al. [53] and referring to “Archaebacteria” while using “Phage” but neither “Virus” nor “Viral.”

The simultaneous timing of the introduction of the terms “Virus” and “Archaebacteria,” and variants, into what up to that point had been nearly indistinguishable from the otherwise “Bacteriophage” literature was not necessarily coincidental. In addition to Fox et al. [53] having been published in the journal *Science* in 1980, and thus widely disseminated well prior to 1982, it can be argued (Anonymous, personal communication) that “This is because Zillig was firmly of the view that archaea have viruses not bacteriophages. Also, because of the influence of Carl Woese, as the first conference on Archaebacteria was held in Germany, around this time (June-July, 1981). …Munich (that Carl attended)… at the very institute that Zillig and Schnabel worked.” As relayed to us by Stedman (personal communication), “the 1981 meeting was at the Max Planck Institute for Biochemistry, (actually just outside Munich in Martinsried) where both Wolfram Zillig (Departmental Director) and Heinke Schnabel (and her husband Ralf) worked as Group leaders.” The concept of archaebacteria thus appears to have been very much a part of the conversation among those individuals who then introduced the concept of “Virus” to what would become the archaeal virus literature.

These trends in usage continued in the following year, 1983. In an article by Rohrmann et al. [55], titled “Bacteriophages of *Halobacterium halobium*: virion DNAs and proteins,” the term “Viruses” is used (“The difference in sizes of the proteins between the two viruses, in addition to the restriction endonuclease fragment patterns, indicate [sic] that these two viruses are not closely related.”). Also used is “Archaebacteria” (“These results indicate that these halophages, the host of which is included among the archaebacteria…”). This article does not cite Fox et al. [53] but does cite an earlier though less prominent article by that same group, one that is titled, simply, “Archaebacteria” [56].

Further though not yet complete trending away from use of “Phage” as a descriptor of these viruses is seen in an article published by Janekovic et al. [57], also in 1983 and on which Zillig serves as a middle author. This paper cites Fox et al. [53], along with the even earlier Woese and Fox [9], and is titled “TTV1, TTV2, and TTV3, a family of viruses of the extremely thermophilic, anaerobic, sulfur reducing archaebacterium, *Thermoproteus tenax*.” The publication is interesting, etymologically, for at least two reasons.

1. This is the first archaeal virus paper for which *Halobacterium* spp. did not serve as hosts. Related to that point, these are the first viruses to be described of kingdom Crenarchaeota, versus the kingdom Euryarchaeota [10], where genus *Halobacterium* is a member of the latter.
2. There is an indication (page 45) of “particles also resembling viruses of eukaryotes rather than “normal” bacteriophages.” In particular, “These viruses are unlike bacteriophages known to date, including halobacteriophage **ϕ**H which resembles phages of eubacteria in many respects.”

We thus have a new host genus and kingdom as well as a new paradigm for the nature of archaeal viruses which, in at least some cases, are somewhat divergent from what is seen among the viruses of bacteria. It is possible that this combined novelty provided some basis for a change in perspective, that is, from describing these infectious agents of domain *Archaea* as “Phages” to instead describing them primarily as “Viruses.” In particular, the observation of virions that were not phage-like was suggestive of a kinship between the viruses of what would come to be known as domain *Archaea* and viruses that otherwise are described simply as “Viruses,” that is, eukaryotic viruses.

Consistent with this perspective, though placing the date of the transition approximately ten years later than as indicated here, is this 2012 sentiment from Felisberto-Rodrigues et al. [58]:

Although viruses infecting archaea are known since the early 1970s [11], they have only been studied in detail very recently. The notion that these viruses constitute a variety of bacteriophages with head and tail (Caudovirales), reinforced by the initial findings, was challenged by the analyses of samples isolated by Zillig and coworkers from extreme environments, rich in hyperthermophilic archaea, including the Icelandic solfatara [59]. These analyses revealed the presence of a large diversity of viral morphotypes, including viruses of linear, spindle-shaped, spherical and more exotic forms, such as drops and bottle-shapes.

## 5. Vestiges of “Phage” to Describe Archaeal Viruses

**Figure 2:**
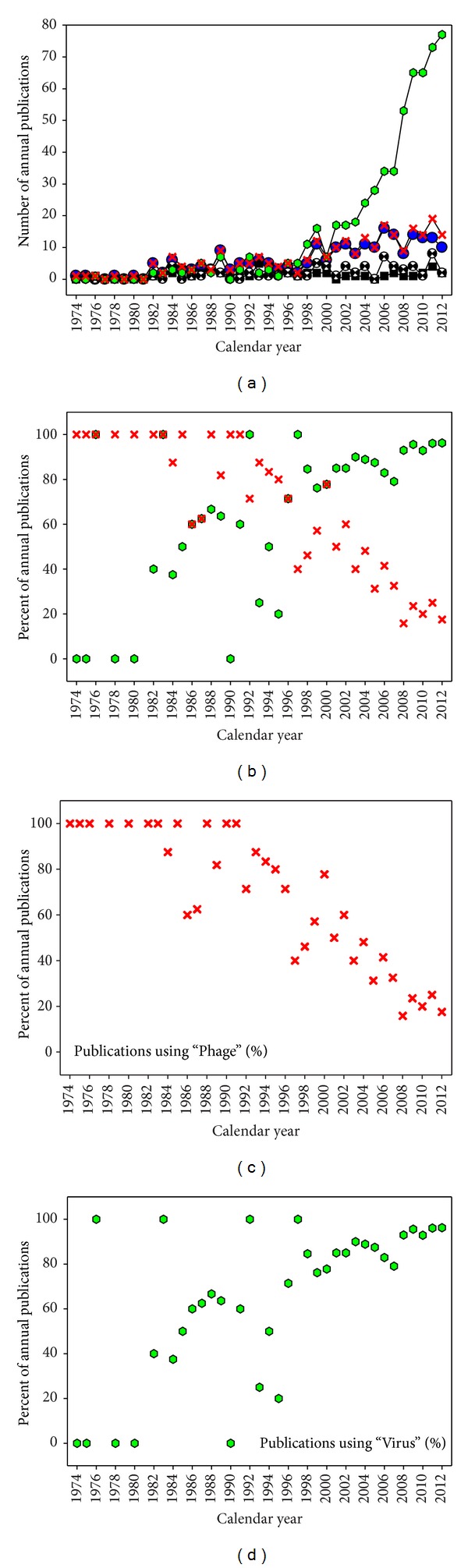
Counting publications using “Virus” (green hexagons), “Phage” (blue circles), “Halophage” (black squares), “Prophage” (“hourglass” circles), and an aggregate of all three phage terms, “All phage” (red Xs) to describe archaeal viruses. Presented are (a) the absolute number of publications, (b) the relative number of “All phage” versus “Virus”, (c) just “All phage”, and (d) just “Virus” (with (c) and (d) provided solely for clarity rather than to provide additional information). Shown are numbers of “Yes” statuses as indicated in Table 1, supplementary materials, but not “Yes/No” nor “No” entries.

Use of “Phage” as a descriptor for viruses of domain *Archaea* would continue at a rate of at least one reference per year to the present. At this point, consideration of these publications is less relevant to the transition to “Archaeal viruses” except for the sake of documenting ongoing use. We thus present these publications primarily in graphical as well as tabular form (Figure 2, and also Table 1, supplementary materials, resp.). In the latter we provide both absolute numbers of usage, by calendar year, as well as relative numbers.

In Figure 2, note particularly the post-2000 rise in absolute numbers of publications that use “Virus” (Figure 2(a)) as well as the associated somewhat steady decline in the number of publications that use “Phage” as a *fraction* of the total number of publications considering archaeal viruses (Figure 2(c)). Indeed, even as “Phage” has persisted in this literature, most of the same publications have also used “Virus” and this has been the case for three quarters or more of these publications that we have examined individually since 1997 (Figure 2(d)). We speculate that a driver of the *ongoing* transition from “Phage” to “Virus,” beyond personal preference by core authors working in the field, is inclusion of the term “Virus” in many names of archaeal virus isolates, such as *Sulfolobus* spindle-shaped virus 1 (SSV1) or *Haloarcula hispanica* pleomorphic virus 1 (HHPV1), for example, as summarized by Krupovic et al. [60].

As there is no centralized authority governing of the naming of archaeal viruses nor central control over whether they are described in names as viruses versus phages, the use of “Virus” in these names not only may help to drive the increasing use of “Virus” in the archaeal virus literature but also can represent a consequence of that trend. Particularly, the “rules” for governing virus naming range from proposals for formal guidelines [61] to the whims of individual discovers, for example, “Corndog” as the name of a bacterial virus [62], and both can be influenced by what usage otherwise is currently trending within a field. It is also conceivable that specific instances of retention or inclusion of the term “Phage” to describe archaeal viruses are a consequence of demands made by editors or reviewers, for example, as appears to be the case for the title of the 1999 Arnold et al. [63] encyclopedia article (K. Stedman, personal communication).

## 6. The “Archaebacterial Virus” to “Archaeal Virus” Transition

A number of descriptors exist that are synonymous with the term “Archaeal virus” as summarized in part in Figure 1, as well as numerous variations in spelling. Among these are terms that describe a subset of such viruses. The latter includes “Halophage” (as also presented in Figure 1), “Haloarchaeophage” (ditto), and “Haloarchaeal virus” as well as “Crenarchaeal virus” and “Euryarchaeal virus” plus additional variations. Notably, the latter three concepts are constructed of a combination of the term “Archaeal” and that of “Virus,” that is, just as is “Archaeal virus” itself. Nonetheless, though use of the term “Archaeal virus” now dominates within publications today, that was not always the case (Table 2, supplementary materials).

As we have considered above, there first was a transition from use of “Phage” to use of “Virus” for what now are known, at least in part, as archaeal viruses. This transition occurred in the early 1980s and it appeared to have coincided—as discussed above—with the introduction of the term “Archaebacteria” into the literature considering their viruses. The transition to “Archaeal virus” by necessity, however, could not occur until the term “*Archaea*” and therefore “Archaeal” came into being, with the former not invented until 1990 [10]. When, then, did the transition to the now familiar “Archaeal virus” actually occur? The answer, also as indicated in Figure 1, and again to the best of our knowledge, is 1993, with a publication by Nölling et al. [20]. Highlighting the transitional aspects of that publication, note that “Archaeal phage” is also used by these authors. The first use of the term “Virus” and “Archaeal” in the same sentence also can be found in a Nölling et al. paper, from 1991 [64] (p. 1981): “The presence of viruses and virus-like particles has been described in various representatives of the archaeal domain…”

Given the forty-year span since the first archaeal virus publication in 1974 [11], the use of “Archaeal virus” thus is both 20 years old this year and has been in use for approximately half of the age of the archaeal virus literature. See Table 2 (supplementary materials) for consideration of various synonyms for “Archaeal virus,” dates of use, and associated publications. All of the latter are also listed in Table 1 (supplementary materials).

## 7. Why Not Phage?

There clearly is a preference within the archaeoviral literature for describing the viruses of domain *Archaea* as “Viruses” rather than as “Phages” (Figure 2), and particularly as “Archaeal viruses” (Table 2, supplementary materials). Even so, is this choice legitimate, particularly given the precedence of use of “Phage” as a description of viruses of prokaryotic organisms? That is, why not “Phage”? Rather than addressing the latter question directly, we instead consider its converse: Why “Phage”?

The term “Phage” appears to have first been applied to bacteriophages to describe a macroscopic phenomenon that is not necessarily always of viral origin, that is, the lysing of bacterial cultures such that, in particular, they are to clear from a turbid state. This transition can be legitimately described as an “eating” or “devouring” as is the Greek origin of “Phage,” or alternatively something that “develops at the expense of something else” [65]. This “expense” is readily seen with phage-infected bacterial cultures, whether in broth or during the formation of plaques. Indeed, such macroscopically visible destruction of cells is not limited to phages as viruses. What *is* unique to phages, unlike other viruses, is that phages were discovered within the context of cell cultures, those of bacteria, rather than having only been subsequently studied within that context (e.g., tissue culture).

Contrasting bacterial phages, the first archaeal virus was discovered, at least initially, *not* specifically within the context of the destruction of a cell culture [11, pp. 680-681]:

During an investigation of flagella from *H. salinarium*, phage particles were observed in some crudely purified flagellar preparations. Phage-containing preparations did not give rise to plaques when plated with exponentially growing *H. salinarium* cells. Attempts at ultraviolet induction of *H. salinarium* cultures resulted in normal death curves without any concomitant phage production. Maintenance of batch cultures, started from isolated *H. salinarium* colonies, in the logarithmic phase of growth, by means of serial transfer of cultures in late log phase to fresh media, resulted in eventual lysis of the cultures. Lysis may occur after the first transfer or be delayed for more than six transfers.

These first archaeal viruses even so were described as phages, as domain *Archaea* had yet to be defined, such that *H. salinarium* was considered to be a bacterium. Is it legitimate therefore for researchers who study these viruses nevertheless to *not* feel beholden to the concept of phages, but instead to explicitly tie these viruses to the larger phenomenon of viruses? Our answer to that question is that it is not that the use of the term “Archaeal virus” should be questioned by phage researches but instead that “Phage” as a descriptor should be tolerated and perhaps even appreciated by virologists due to its historical roots and ubiquity of use. There should be no requirement, in other words, for emulation of use of the term “Phage” by other virologists, whether those individuals choose to study the viruses of eukaryotes, archaeans, or even of bacteria. Indeed, it is quite clear that those who study the viruses of *Archaea* prefer “Virus” to “Phage” as a descriptor of those infectious agents (Figure 2).

These latter statements certainly should not be construed as any advocacy for avoidance, particularly within the bacterial virus literature, of the use of “Phage” to describe bacterial viruses. Indeed, its use such as in abstracts and titles can be helpful to the extent that it aids other phage biologists—that is, bacterial virus researchers—in identifying phage publications. It is important nevertheless to acknowledge both the legitimacy and, in many or most cases, the *greater* legitimacy of the term “Virus” as a general descriptor of acellular but encapsidated infectious agents. For example, from Prangishvili [65], page 551:

It did not take d'Herelle long to realize that bacteriophages were the same type of biological entity as viruses of plants and animals. At this point, it would probably have been advantageous to play down the term “bacteriophage” and favor instead the more general term “bacterial virus”. In any case, d'Herelle's concept of bacteriophages as viruses of bacteria was widely accepted only much later, in the late 1930s.

Had viruses been discovered generally in a manner that was equivalent to that of phages, that is, within the context of the lysis and/or destruction of a cell culture, then it may have been legitimate to describe or at least to first have described all viruses as phages. Historically, however, this was not the case. The concept of viruses thus predates that of phages and so possesses greater legitimacy as a descriptor of what today universally are known as viruses, that is, than does the concept of phages.

## 8. Viruses, Tailed Viruses, Proviruses, and Haloviruses

An additional manner of considering the ideas covered in the previous section is that the concept of “Phage” is not synonymous with that of “Virus.” Certainly there is overlap, and particularly so in terms of how we think of the viruses of domain *Bacteria.* It is important though to resist the temptation to use “Phage” simply as a means of avoiding being overly repetitive in the use of “Virus.” If a virus of a prokaryotic host is not a bacterial virus then it is not a bacteriophage. Consequently, it would be suspect to call that virus a phage.

Similarly, it is important to keep in mind that the concept of “Tailed phage” is not identical to that of “Phage.” There are in particular phages that possess tails along with phages that lack tails, for example, [22, 66, 67]. It therefore does not logically follow that if a virus is a phage then it possesses a tail, nor, given that there are a number of archaeal viruses which also possess tails, that if a virus possesses a tail then it is a phage, that is, as equivalent to bacteriophage or bacterial virus. One can also consider the lack of complete overlap between the concept of “Prophage” and that of “Provirus,” with “Prophage” legitimately used to describe a provirus only to the extent that using “Phage” would also be a legitimate description of that virus. In each of these instances, to the extent that a trend exists among researchers to limit application of the term “Phage” in describing archaeal viruses (Figure 2), then it is reasonable to mostly avoid the use of “Phage” even as a qualified term in the archaeal virus literature. In particular, “Phage” as a synonym for “Tailed virus” is *not* a useful shorthand.

The term “Halophage” is by contrast less problematic and especially so when used as a general term, as in “Environmental halophages,” though to our knowledge it is not sanctioned by any governing body, such as ICTV. Even here, though, it can be preferable to avoid the concept of “Phage” altogether by using “Halovirus” instead of “Halophage” since “Halovirus” implies neither that a virus is archaeal nor bacterial. Indeed, just as prophages are only a subset of proviruses, tailed phages only a subset of tailed viruses, and bacteriophages only a subset of all viruses, halophages could be considered to represent only a subset of haloviruses. Though complicated, it is also possible to use the phrase “Haloarchaeal virus” or its equivalent if there is a need to distinguish between haloviruses in terms of the domain of their hosts, versus, for example, “Halobacterial virus.”

## 9. Conclusion

The viruses of domain *Archaea* were identified prior to appreciation of the existence of domain *Archaea* itself. Before introduction of the three-domain system of classification, it therefore was reasonable to describe these viruses as phages of bacteria, that is, as bacteriophages. Following the discovery of the concept of archaebacteria there appears to have been a shift away not just from “Bacteria” (as in bacteriophage) but also from use of “Phage” in describing these viruses, a shift that began in earnest especially during the early 1980s (Figure 2, and also Tables 1 and 2, supplementry materials). One then sees further movement during the early 1990s towards the use of “Archaeal viruses” as a descriptor (Table 2, supplementary materials).

Movement away from “Phage” has not been complete in considering archaeal viruses and we feel that there are various drivers towards retention of “Phage” or its derivatives to describe them. These drivers include inadvertent use (where an article otherwise uses “Virus” but in one or a few places “Phage” is substituted, in certain cases seemingly accidentally), failure to adequately distinguish between the concepts of “Phage” and “Virus” as applied to archaeal viruses, use of the term “Phage” to generally describe head-and-tail viruses, use of “Phage” within the context of “Halophage” (Table 1, supplementary materials, though “Halovirus” or “Haloarchaeal virus” are legitimate or even preferable substitutes), and the use of “Phage” within the context of “Prophage” (Table 1, supplementary materials, where “Provirus” would be preferable in the case of archaeal viruses). In addition, perhaps a special case is the use of “Phage” to describe specific archaeal virus isolates, such as “*Methanobacterium* phage ΨM1”. Also important towards impeding the transition from “Phage” to “Virus” in the archaeal virus literature has been a lumping together of bacteriophages and archaeal viruses in important databases [68].

Alternatives to “Archaeal virus” versus “Bacterial virus” versus “Eukaryotic virus” have been proposed by a number of authors. These include archaeovirus, bacteriovirus, and eukaryovirus as suggested by Raoult and Forterre [30] as well as Soler et al. [31], the slight variations of archeovirus, bacteriovirus, and eukaryavirus of Forterre and Prangishvili [32] or archeovirus, bacteriovirus, and eukaryovirus as presented as well by Pina et al. [69], and the bacterioviruses, archaealviruses, and eukaryalviruses of Comeau et al. [70]. See also archaebacteriophage versus eubacteriophage [71]. While we appreciate the unifying nature of these approaches, we do not necessarily advocate their general adoption if that would come at the expense of “Bacteriophage” (or “Phage”), “Archaeal virus,” or simply “Virus” as descriptors of the viruses of members of domains *Bacteria*, *Archaea*, and *Eukarya*, respectively.

Within publications in which archaeal viruses are discussed, we nonetheless feel that the use of “Phage” should be limited in favor of “Archaeal virus” for viruses of domain *Archaea* and “Bacterial virus” for viruses of domain *Bacteria*, or alternatively the various “Virus-” based proposed alternatives as listed immediately above. We suggest this merely for the sake of limiting ambiguity within the archaeal virus literature, with a clear distinction therefore maintained between archaeal viruses on the one hand and bacterial viruses on the other. By contrast, within the bacterial virus literature, or when referring to specific phages (e.g., phage T4 or phage *λ*), it is neither likely nor necessarily desirable to abandon or reduce use of “Phage” or “Bacteriophage” as a general descriptor of the viruses of domain *Bacteria*.

## Supplementary Material

Provided are annotated summaries of the archaeal virus literature, including 694 references as available before January 1 of 2013. Highlighted per reference is the use of either “Phage” or “Virus” within the context of archaeal viruses. Shown also is a tabular time line of the use and introduction of additional terms employed as synonyms for “Archaeal virus”.
