# Supplementary material for: Archaeal Viruses, Not Archaeal Phages: An Archaeological Dig
Source: Archaea. 2013 Apr 7;2013:251245. doi: 10.1155/2013/251245 (PMC3638648; doi:10.1155/2013/251245)
Supplement: Supplementary file 1 — Provided are annotated summaries of the archaeal virus literature, including 694 references as available before January 1 of 2013. Highlighted per reference is the use of either “Phage” or “Virus” within the context of archaeal viruses. Shown also is a tabular time line of the use and introduction of additional terms employed as synonyms for “Archaeal virus”. [file 251245.f1.docx]

**Archaeal *Viruses*, not Archaeal *Phages*:**

**An *Archae*ological Dig**

**Stephen T. Abedon^1^^[[1]](#footnote-1)^* and Kelly L. Murray^1^**

## ^1^Department of Microbiology, The Ohio State University; Mansfield, Ohio USA

**Supplemental Materials**

[Table 1](#Table_1): Use of “Phage” and “Virus” in the Archaeal Virus Literature (pp. 20-43)

[Table 2](#Table_2): Various Synonyms or Approximate Synonyms to “Archaeal Virus”

and their Appearance in the Literature. (pp. 44-54)

[References](#References): A Total of 694 (pp. 55-115)

**Table 1: Use of “Phage” and “Virus” in the Archaeal Virus Literature**

|  | **Author(s)** | **Year** | **…phage^[[2]](#footnote-2)^** | **Phage or bacteriophage^[[3]](#footnote-3)^** | **Halophage^[[4]](#footnote-4)^** | **Prophage** | **Virus or viral?^[[5]](#footnote-5)^** |
| --- | --- | --- | --- | --- | --- | --- | --- |
| [1] | Torsvik & Dundas | 1974 | Yes | Yes | No | No | No |
| [2] | Wais *et al.* | 1975 | Yes | Yes | Yes | No | No |
| [3] | Stube *et al.* | 1976 | Yes | Yes/No^[[6]](#footnote-6)^ | Yes | No | Yes |
| [4] | Torsvik & Dundas | 1978 | Yes | Yes | No^[[7]](#footnote-7)^ | No | No |
| [5] | Torsvik & Dundas | 1980 | Yes | Yes | No | No | No |
| [6] | Pauling | 1982 | Yes | Yes | Yes | No | No |
| [7] | Reanney & Ackermann | 1982 | Yes | Yes | No | No | No |
| [8] | Schnabel *et al.* | 1982 | Yes | Yes | No | No | Yes |
| [9] | Schnabel *et al.* | 1982 | Yes | Yes | No | No | No |
| [10] | Yeats *et al.* | 1982 | Yes | Yes | No | Yes | Yes |
| [11] | Janekovic *et al.* | 1983 | Yes | Yes | No | No | Yes |
| [12] | Rohrmann *et al.* | 1983 | Yes | Yes | Yes | No | Yes |
| [13] | Barbeyron *et al.* | 1984 |  | No | No | No | Yes |
| [14] | Daniels & Wais | 1984 | Yes | Yes | Yes | No | No |
| [15] | Jarrell & Sprott | 1984 | Yes | No | Yes | No | No |
| [16] | Martin *et al.* | 1984 | Yes | Yes | No | No | Yes |
| [17] | Schnabel | 1984 | Yes | Yes | No | Yes | No |
| [18] | Schnabel | 1984 | Yes | Yes | No | Yes | No |
| [19] | Schnabel & Zillig | 1984 | Yes | Yes | No | Yes | No |
| [20] | Schnabel *et al.* | 1984 | Yes | Yes | No | Yes | Yes |
| [21] | Patterson & Pauling | 1985 | Yes | Yes | Yes | No | Yes |
| [22] | Prangishvili *et al.* | 1985 | Yes | No | Yes | No | No |
| [23] | Wais & Daniels | 1985 | Yes | Yes | Yes | No | No |
| [24] | Zillig *et al.* | 1985 | Yes | Yes | No | No | Yes |
| [25] | Bertani & Baresi | 1986 | Yes | Yes | No | No | No |
| [26] | Nadal *et al.* | 1986 |  | No | No | No | Yes |
| [27] | Vogelsang-Wenke & Oesterhelt | 1986 | Yes | Yes | Yes | No | No |
| [28] | Zillig *et al.* | 1986 | Yes | Yes | No | Yes | Yes |
| [29] | Zillig *et al.* | 1986 |  | No | No | No | Yes |
| [30] | Cline & Doolittle | 1987 | Yes | Yes | Yes | No | No |
| [31] | Ebert *et al.* | 1987 | Yes | Yes | Yes | No | No |
| [32] | Reiter *et al.* | 1987 |  | No | No | No | Yes |
| [33] | Reiter *et al.* | 1987 | Yes | Yes | No | No | Yes |
| [34] | Reiter *et al.* | 1987 | Yes | Yes | No | No | Yes |
| [35] | Sonea | 1987 | Yes | No | No | Yes | No |
| [36] | Woese | 1987 |  | No | No | No | Yes |
| [37] | Zillig *et al* | 1987 |  | No | No | No | Yes |
| [38] | Reiter *et al.* | 1988 | Yes | Yes | Yes | Yes | Yes |
| [39] | Vogelsang-Wenke & Oesterhelt | 1988 | Yes | Yes | Yes | Yes | No |
| [40] | Zillig *et al.* | 1988 | Yes | Yes | Yes | Yes | Yes |
| [41] | Brown *et al.* | 1989 | Yes | Yes | No | Yes | Yes |
| [42] | Cline *et al.* | 1989 | Yes | Yes | No | No | No |
| [43] | Gropp & Oesterhelt | 1989 | Yes | Yes | Yes | No | No |
| [44] | Gropp *et al.* | 1989 | Yes | Yes | No | Yes | Yes |
| [45] | Hackett & DasSarma | 1989 | Yes | Yes | Yes | No | No |
| [46] | Jordan *et al.* | 1989 | Yes | Yes | No | No | No |
| [47] | Meile *et al.* | 1989 | Yes | Yes | No | No | Yes |
| [48] | Reiter *et al.* | 1989 |  | No | No | No | Yes |
| [49] | Schauer & Whitman | 1989 |  | Yes/No^[[8]](#footnote-8)^ | No | No | Yes |
| [50] | Wood *et al.* | 1989 | Yes | Yes | No | No | Yes |
| [51] | Wünsche | 1989 | Yes | Yes | No | Yes/No | Yes |
| [52] | Daniels & Wais | 1990 | Yes | Yes | Yes | No | No |
| [53] | Juez *et al.* | 1990 | Yes | Yes | No | No | No |
| [54] | Meile *et al.* | 1990 | Yes | Yes | No | No | No |
| [55] | Hüdepohl *et al.* | 1991 | Yes | Yes | No | No | No |
| [56] | Ken & Hackett | 1991 | Yes | Yes | Yes | No | Yes |
| [57] | Krebs *et al.* | 1991 | Yes | Yes | Yes | No | No |
| [58] | Nölling *et al.* | 1991 | Yes | Yes | No | No | Yes |
| [59] | Palm *et al.* | 1991 | Yes | Yes | No | No | Yes |
| [60] | Campbell | 1992 |  | No | No | No | Yes |
| [61] | Charbonnier *et al.* | 1992 | Yes | Yes | No | No | Yes |
| [62] | Gropp *et al.* | 1992 | Yes | Yes | No | Yes | Yes |
| [63] | Koonin *et al.* | 1992 |  | No | No | No | Yes |
| [64] | Reeve | 1992 | Yes | Yes | No | No | Yes |
| [65] | Schleper *et al.* | 1992 | Yes | Yes | No | Yes | Yes |
| [66] | Stolt & Zillig | 1992 | Yes | Yes | Yes | No | Yes |
| [67] | Koonin & Ilyina | 1993 |  | No | No | No | Yes |
| [68] | Krebs *et al.* | 1993 | Yes | No | Yes | No | No |
| [69] | Nölling *et al.* | 1993 | Yes | Yes | No | Yes | Yes |
| [70] | Nuttall & Dyall-Smith | 1993 | Yes | Yes | Yes | No | No |
| [71] | Nuttall & Dyall-Smith | 1993 | Yes | Yes | Yes | No | No |
| [72] | Stolt & Zillig | 1993 | Yes | Yes | No | No | No |
| [73] | Stolt & Zillig | 1993 | Yes | Yes | Yes | No | No |
| [74] | Stolt & Zillig | 1993 | Yes | Yes | Yes | No | No |
| [75] | Charbonnier & Forterre | 1994 | Yes | Yes | No | No | Yes |
| [76] | Lehmacher & Klenk | 1994 |  | No | No | No | Yes |
| [77] | Oren | 1994 | Yes | Yes | Yes | No | No |
| [78] | Stolt & Zillig | 1994 | Yes | Yes | Yes | No | No |
| [79] | Stolt *et al.* | 1994 | Yes | Yes | Yes | Yes | Yes/No |
| [80] | Zillig *et al.* | 1994 | Yes | Yes | Yes | No | Yes |
| [81] | Bouyoub *et al.* | 1995 | Yes | Yes | No | No | No |
| [82] | Ciaramella *et al.* | 1995 |  | No | No | No | Yes |
| [83] | Holmes *et al.* | 1995 | Yes | No | Yes | No | No |
| [84] | Nuttall & Dyall-Smith | 1995 | Yes | Yes | Yes | No | Yes/No |
| [85] | Stettler *et al.* | 1995 | Yes | Yes | No | Yes | No |
| [86] | Aagaard *et al.* | 1996 |  | No | No | No | Yes |
| [87] | Ackermann | 1996 | Yes | Yes | No | Yes | Yes |
| [88] | Erauso *et al.* | 1996 |  | No | No | No | Yes |
| [89] | Goel *et al.* | 1996 | Yes | Yes | No | No | No |
| [90] | Guixa-Boixareu *et al.* | 1996 | Yes | Yes | Yes | No | Yes |
| [91] | Newbold *et al.* | 1996 | Yes | Yes | No | No | No |
| [92] | Zillig *et al.* | 1996 | Yes | No | Yes | Yes | Yes |
| [93] | Chiura *et al.* | 1997 |  | Yes/No^[[9]](#footnote-9)^ | No | No | Yes^[[10]](#footnote-10)^ |
| [94] | Esposito & Scocca | 1997 |  | No | No | No | Yes |
| [95] | Oren *et al.* | 1997 | Yes | Yes | No | No | Yes |
| [96] | Tumbula *et al.* | 1997 |  | No | No | No | Yes |
| [97] | Witte *et al.* | 1997 | Yes | Yes | Yes | Yes | Yes |
| [98] | Bath *et al.* | 1998 | Yes | No | Yes^[[11]](#footnote-11)^ | No | Yes |
| [99] | Bernander | 1998 |  | No | No | No | Yes |
| [100] | Cannio *et al.* | 1998 |  | No | No | No | Yes |
| [101] | Charlebois *et al.* | 1998 |  | No | No | No | Yes |
| [102] | Daniels & Wais | 1998 | Yes | Yes | Yes | No | No |
| [103] | Höök-Nikanne *et al.* | 1998 | Yes | Yes | No | No | No |
| [104] | Maniloff & Ackermann | 1998 | Yes | Yes | No | No | Yes |
| [105] | Pfister *et al.* | 1998 | Yes | Yes | No | Yes | Yes |
| [106] | Prangishvili *et al.* | 1998 |  | No | No | No | Yes |
| [107] | Salmi *et al.* | 1998 |  | No | No | No | Yes |
| [108] | Van der Oost *et al.* | 1998 | Yes | Yes | No | No | Yes |
| [109] | Ventosa *et al.* | 1998 |  | No | No | No | Yes |
| [110] | Zillig *et al.* | 1998 |  | No | No | No | Yes |
| [111] | Ackermann | 1999 | Yes | Yes | No | Yes | Yes |
| [112] | Andrade *et al.* | 1999 |  | No | No | No | Yes |
| [113] | Arnold *et al.* | 1999 |  | No | No | No | Yes |
| [114] | Arnold *et al.* | 1999 | Yes | Yes^[[12]](#footnote-12)^ | Yes | Yes | Yes |
| [115] | Baldo & McClure | 1999 |  | No | No | No | Yes |
| [116] | Bertani | 1999 | Yes | Yes | No | Yes | Yes |
| [117] | Conrad *et al.* | 1999 |  | No | No | No | Yes |
| [118] | Eiserling *et al.* | 1999 | Yes | Yes | No | No | Yes |
| [119] | Forterre | 1999 |  | No | No | No | Yes |
| [120] | Hendrix | 1999 |  | No | No | No | Yes |
| [121] | Hochheimer *et al.* | 1999 | Yes | No | No | Yes | No |
| [122] | Jarrell *et al.* | 1999 | Yes | Yes | No | No | Yes |
| [123] | Klieve & Hegarty | 1999 | Yes | Yes | No | No | Yes |
| [124] | Leigh | 1999 | Yes | Yes | Yes | No | No |
| [125] | Makarova *et al.* | 1999 | Yes | Yes | No | No | No |
| [126] | Makino *et al.* | 1999 | Yes | Yes | No | Yes | No |
| [127] | Noll *et al.* | 1999 |  | No | No | No | Yes |
| [128] | Prangishvili *et al.* | 1999 | Yes | Yes^[[13]](#footnote-13)^ | No | No | Yes |
| [129] | Stedman *et al.* | 1999 |  | No | No | No | Yes |
| [130] | Tumbula & Whitman | 1999 | Yes | Yes | No | No | No |
| [131] | Whitman *et al.* | 1999 |  | No | No | No | Yes |
| [132] | Arnold *et al.* | 2000 | Yes | No | Yes | No | Yes |
| [133] | Arnold *et al.* | 2000 | Yes | Yes | Yes | Yes | Yes |
| [134] | Baranyi *et al.* | 2000 | Yes | Yes | No | Yes | Yes |
| [135] | Klein *et al.* | 2000 | Yes | Yes | No | Yes | Yes |
| [136] | Martusewitsch *et al.* | 2000 |  | No | No | No | Yes |
| [137] | Peng *et al.* | 2000 |  | No | No | No | Yes |
| [138] | Wang *et al.* | 2000 | Yes | Yes | No | No | No |
| [139] | Wasserfallen *et al.* | 2000 | Yes | Yes | No | Yes | No |
| [140] | Wommack & Colwell | 2000 | Yes | Yes | No | No | Yes |
| [141] | Ackermann | 2001 | Yes | Yes | No | Yes/No^[[14]](#footnote-14)^ | Yes |
| [142] | Bell | 2001 |  | No | No | No | Yes |
| [143] | Birkenbihl *et al.* | 2001 |  | No | No | No | Yes |
| [144] | Blum *et al.* | 2001 |  | No | No | No | Yes |
| [145] | Brügger *et al.* | 2001 |  | No | No | No | Yes |
| [146] | Cannio *et al.* | 2001 |  | No | No | No | Yes |
| [147] | Harty *et al.* | 2001 |  | No | No | No | Yes |
| [148] | Hofer & Sommaruga | 2001 | Yes | Yes | No | No | Yes/No |
| [149] | Iyer *et al.* | 2001 |  | No | No | No | Yes |
| [150] | Kvaratskhelia *et al.* | 2001 |  | No | No | No | Yes |
| [151] | Lange & Ahring | 2001 | Yes | Yes | No | No | Yes |
| [152] | Luo & Wasserfallen | 2001 | Yes | Yes | No | No | Yes |
| [153] | Luo *et al.* | 2001 | Yes | Yes | No | Yes | No |
| [154] | Luo *et al.* | 2001 | Yes | Yes | No | No | Yes |
| [155] | McClure | 2001 |  | No | No | No | Yes |
| [156] | Peng *et al.* | 2001 | Yes | Yes | No | No | Yes |
| [157] | Prangishvili *et al.* | 2001 | Yes | Yes^[[15]](#footnote-15)^ | No | No | Yes |
| [158] | Rice *et al.* | 2001 |  | No | No | No | Yes |
| [159] | Sinkovics | 2001 | Yes | Yes | No | No | Yes |
| [160] | Williamson *et al.* | 2001 | Yes | Yes | No | No | No |
| [161] | Ackermann | 2002 | Yes | Yes | No | Yes | Yes |
| [162] | Bettstetter *et al.* | 2002 |  | No^[[16]](#footnote-16)^ | No | No | Yes |
| [163] | Ciaramella *et al.* | 2002 |  | No | No | No | Yes |
| [164] | Desplats & Krisch | 2002 | Yes | Yes | No | No | Yes |
| [165] | Filée *et al.* | 2002 | Yes | Yes | No | No | Yes |
| [166] | Hendrix | 2002 | Yes | Yes^[[17]](#footnote-17)^ | No | No | Yes |
| [167] | Klein *et al.* | 2002 | Yes | Yes^[[18]](#footnote-18)^ | No | Yes | Yes |
| [168] | Kvaratskhelia *et al.* | 2002 |  | No | No | No | Yes |
| [169] | Lang *et al.* | 2002 | Yes | Yes | No | No | No |
| [170] | Lawrence *et al.* | 2002 | Yes | Yes | No | No | Yes |
| [171] | Lucas *et al.* | 2002 |  | No | No | No | Yes |
| [172] | Luo *et al.* | 2002 | Yes | Yes | No | Yes | No |
| [173] | Mitchell *et al.* | 2002 | Yes | No | Yes | No | No |
| [174] | Neef *et al.* | 2002 |  | No | No | No | Yes |
| [175] | Paranen *et al.* | 2002 |  | No | No | No | Yes |
| [176] | Rachel *et al.* | 2002 | Yes | Yes^[[19]](#footnote-19)^ | No | No | Yes |
| [177] | Schiraldi *et al.* | 2002 |  | No | No | No | Yes |
| [178] | Serre *et al.* | 2002 | Yes | Yes^[[20]](#footnote-20)^ | No | Yes^[[21]](#footnote-21)^ | Yes |
| [179] | She *et al.* | 2002 | Yes | Yes | No | No | Yes |
| [180] | Tang *et al.* | 2002 |  | Yes/No^[[22]](#footnote-22)^ | No | No | Yes |
| [181] | Bamford | 2003 |  | No | No | No | Yes |
| [182] | Bartolucci *et al.* | 2003 |  | No | No | No | Yes |
| [183] | Casjens | 2003 | Yes | Yes | No | No | No |
| [184] | Cohen *et al.* | 2003 |  | No | No | No | Yes |
| [185] | Contursi *et al.* | 2003 |  | No | No | No | Yes |
| [186] | Dyall-Smith *et al.* | 2003 | Yes | Yes | Yes | No | Yes |
| [187] | Faguy | 2003 | Yes | Yes | No | No | No |
| [188] | Filée *et al.* | 2003 | Yes | Yes | No | Yes/No^[[23]](#footnote-23)^ | Yes |
| [189] | Geslin *et al.* | 2003 |  | No | No | No | Yes |
| [190] | Geslin *et al.* | 2003 | Yes | Yes | No | Yes | Yes |
| [191] | Hendrix | 2003 | Yes | Yes | No | Yes | Yes |
| [192] | Jonuscheit *et al.* | 2003 | Yes | Yes^[[24]](#footnote-24)^ | No | No | Yes |
| [193] | Li *et al.* | 2003 |  | No | No | No | Yes |
| [194] | Prangishvili | 2003 | Yes | Yes^[[25]](#footnote-25)^ | No | No | Yes |
| [195] | Roberts *et al.* | 2003 |  | No | No | No | Yes |
| [196] | Rossi *et al.* | 2003 |  | No | No | No | Yes |
| [197] | Sato *et al.* | 2003 |  | No | No | No | Yes |
| [198] | Serre and Duguet | 2003 |  | Yes/No^^[[26]](#footnote-26)^^ | No | No | Yes |
| [199] | Snyder *et al.* | 2003 |  | No | No | No | Yes |
| [200] | Stedman *et al.* | 2003 |  | No | No | No | Yes |
| [201] | Benson *et al.* | 2004 |  | No | No | No | Yes |
| [202] | Boadi *et al.* | 2004 | Yes | Yes | No | No | Yes |
| [203] | Bonneau *et al.* | 2004 | Yes | Yes | No | Yes | Yes |
| [204] | Breitbart *et al.* | 2004 | Yes | Yes | No | Yes/No | Yes/No |
| [205] | Cheng *et al.* | 2004 | Yes | Yes | No | Yes | Yes/No |
| [206] | Chiura | 2004 |  | No | No | No | Yes |
| [207] | Häring *et al.* | 2004 | Yes | Yes^[[27]](#footnote-27)^ | No | No | Yes |
| [208] | Hendrix | 2004 | Yes | Yes^[[28]](#footnote-28)^ | No | No | Yes |
| [209] | Iyer *et al.* | 2004 |  | No | No | No | Yes |
| [210] | Jones | 2004 |  | No | No | No | Yes |
| [211] | Kessler *et al.* | 2004 |  | No | No | No | Yes |
| [212] | Kraft *et al.* | 2004 |  | No | No | No | Yes |
| [213] | Kraft *et al.* | 2004 |  | No | No | No | Yes |
| [214] | Lipps | 2004 |  | No | No | No | Yes |
| [215] | Liu *et al.* | 2004 | Yes | Yes | No | No | Yes |
| [216] | Mitchell & Rao | 2004 | Yes | Yes | Yes | No | No |
| [217] | Peng *et al.* | 2004 |  | No^[[29]](#footnote-29)^ | No | No | Yes |
| [218] | Prangishvili & Garrett | 2004 |  | No | No | No | Yes |
| [219] | Prieur *et al.* | 2004 | Yes | No | No | Yes | Yes |
| [220] | Rice *et al.* | 2004 |  | No | No | No | Yes |
| [221] | Rössler *et al.* | 2004 |  | No | No | No | Yes |
| [222] | Snyder *et al.* | 2004 |  | No | No | No | Yes |
| [223] | Tang *et al.* | 2004 | Yes | No | No | Yes | Yes |
| [224] | Venter *et al.* | 2004 | Yes | Yes | No | No | No |
| [225] | Ventura *et al.* | 2004 | Yes | Yes | No | No | Yes |
| [226] | Weinbaur | 2004 | Yes | Yes | No | No | Yes |
| [227] | Wiedenheft *et al.* | 2004 |  | No | No | No | Yes |
| [228] | Winter *et al.* | 2004 |  | No | No | Yes/No | Yes |
| [229] | Allers & Mevarech | 2005 | Yes | Yes | No | No | Yes |
| [230] | Bamford *et al.* | 2005 |  | No | No | No | Yes |
| [231] | Breitbart & Rohwer | 2005 |  | No | No | No | Yes |
| [232] | Carlson | 2005 | Yes | Yes | No | No | No |
| [233] | Desselberger | 2005 |  | No | No | No | Yes |
| [234] | Ennifar *et al.* | 2005 |  | No | No | No | Yes |
| [235] | Fernández *et al.* | 2005 | Yes | Yes | No | No | Yes |
| [236] | Fukui *et al.* | 2005 |  | No | No | No | Yes |
| [237] | Häring *et al.* | 2005 |  | No | No | No | Yes |
| [238] | Häring *et al.* | 2005 |  | No | No | No | Yes |
| [239] | Häring *et al.* | 2005 |  | No | No | No | Yes |
| [240] | Khayat *et al.* | 2005 |  | No | No | No | Yes |
| [241] | Koike *et al.* | 2005 | Yes | Yes | No | No | No |
| [242] | Laurinmäki *et al.* | 2005 |  | No | No | No | Yes |
| [243] | Liu & Yang | 2005 |  | No | No | No | Yes |
| [244] | Martiny & Field | 2005 | Yes | Yes | No | No | Yes/No |
| [245] | Mojica *et al.* | 2005 | Yes | Yes | No | Yes | Yes |
| [246] | Namba *et al.* | 2005 | Yes | Yes | No | No | Yes |
| [247] | Parker & White | 2005 |  | No | No | No | Yes |
| [248] | Paukner *et al.* | 2005 | Yes | Yes | No | No | No |
| [249] | Porter *et al.* | 2005 |  | No | No | No | Yes |
| [250] | Prangishvili & Garrett | 2005 |  | No | No | No | Yes |
| [251] | Randau *et al.* | 2005 |  | No | No | No | Yes |
| [252] | Saren *et al.* | 2005 |  | No | No | No | Yes |
| [253] | Strömsten *et al.* | 2005 |  | No | No | No | Yes |
| [254] | Suhre *et al.* | 2005 |  | No | No | No | Yes |
| [255] | Teixeira & Gilson | 2005 |  | No | No | No | Yes |
| [256] | Umadevi *et al.* | 2005 |  | No | No | No | Yes |
| [257] | Vestergaard *et al.* | 2005 |  | No | No | No | Yes |
| [258] | Villarreal | 2005 | Yes | Yes | No | No | Yes |
| [259] | Xiang *et al.* | 2005 | Yes | Yes | No | No | Yes |
| [260] | Young *et al.* | 2005 |  | No | No | No | Yes |
| [261] | Ahn *et al.* | 2006 | Yes | Yes | No | No | Yes |
| [262] | Albers *et al.* | 2006 |  | No | No | No | Yes |
| [263] | Aucelli *et al.* | 2006 |  | No | No | No | Yes |
| [264] | Bath *et al.* | 2006 | Yes | No | No | Yes | Yes |
| [265] | Bose *et al.* | 2006 | Yes | Yes | No | Yes | No |
| [266] | Burnett | 2006 |  | No | No | No | Yes |
| [267] | Contursi *et al.* | 2006 |  | No | No | No | Yes |
| [268] | Dorazi *et al.* | 2006 |  | No | No | No | Yes |
| [269] | Durand *et al.* | 2006 |  | No | No | No | Yes |
| [270] | Erauso *et al.* | 2006 |  | No | No | No | Yes |
| [271] | Forterre | 2006 |  | No | No | No | Yes |
| [272] | Forterre | 2006 |  | No | No | No | Yes^[[30]](#footnote-30)^ |
| [273] | Goulet *et al.* | 2006 |  | No | No | No | Yes |
| [274] | Kessler *et al.* | 2006 |  | No | No | No | Yes |
| [275] | Kivelä *et al.* | 2006 |  | No | No | No | Yes |
| [276] | Koonin | 2006 | Yes | Yes^[[31]](#footnote-31)^ | No | No | Yes |
| [277] | Koonin & Dolja | 2006 |  | Yes/No^[[32]](#footnote-32)^ | No | No | Yes |
| [278] | Koonin *et al.* | 2006 | Yes | Yes^[[33]](#footnote-33)^ | No | No | Yes |
| [279] | Larson *et al.* | 2006 |  | No | No | No | Yes |
| [280] | Legault *et al.* | 2006 | Yes | Yes | No | No | No |
| [281] | Leplae iet al. | 2006 |  | No | No | No | Yes |
| [282] | Lillestøl *et al.* | 2006 | Yes | Yes | No | Yes | Yes |
| [283] | Lipps | 2006 |  | No | No | No | Yes |
| [284] | Maaty *et al.* | 2006 |  | No | No | No | Yes |
| [285] | Mayo & Ball | 2006 |  | No | No | No | Yes |
| [286] | Molineux | 2006 | Yes | Yes | No | No | Yes/No^[[34]](#footnote-34)^ |
| [287] | Nakamura *et al.* | 2006 | Yes | Yes | No | Yes | No |
| [288] | Naryshkina *et al.* | 2006 | Yes | Yes | No | No | Yes/No^[[35]](#footnote-35)^ |
| [289] | Onimatsu *et al.* | 2006 |  | No | No | No | Yes |
| [290] | Ortmann *et al.* | 2006 |  | No | No | No | Yes |
| [291] | Prangishvili *et al.* | 2006 |  | No | No | No | Yes |
| [292] | Prangishvili *et al.* | 2006 | Yes | Yes^[[36]](#footnote-36)^ | No | No | Yes |
| [293] | Prangishvili *et al.* | 2006 | Yes | Yes | No | Yes | Yes |
| [294] | Rass & West | 2006 |  | No | No | No | Yes |
| [295] | Ratel *et al.* | 2006 |  | No | No | No | Yes |
| [296] | Skurnik & Strauch | 2006 | Yes | Yes | No | No | Yes |
| [297] | Stedman *et al.* | 2006 |  | No | No | No | Yes |
| [298] | Stedman *et al.* | 2006 | Yes | Yes^[[37]](#footnote-37)^ | Yes | Yes | Yes |
| [299] | Steenbakkers *et al.* | 2006 | Yes | Yes | No | Yes | No |
| [300] | Witzany | 2006 | Yes | Yes | No | No | Yes^[[38]](#footnote-38)^ |
| [301] | Worning *et al.* | 2006 | Yes | Yes | No | No | No |
| [302] | Ackermann | 2007 | Yes | Yes | No | No^[[39]](#footnote-39)^ | Yes |
| [303] | Ackermann & Kropinski | 2007 | Yes | Yes^[[40]](#footnote-40)^ | No | No | Yes |
| [304] | Akita *et al.* | 2007 | Yes | Yes^[[41]](#footnote-41)^ | No | Yes | Yes |
| [305] | Allen *et al.* | 2007 | Yes | Yes | No | Yes | No |
| [306] | Alzhanova *et al.* | 2007 |  | No | No | No | Yes |
| [307] | Barrangou *et al.* | 2007 | Yes | Yes^[[42]](#footnote-42)^ | No | No | Yes/No^[[43]](#footnote-43)^ |
| [308] | Brügger *et al.* | 2007 |  | No | No | No | Yes |
| [309] | Clore & Stedman | 2007 |  | No | No | No | Yes |
| [310] | Contursi *et al.* | 2007 |  | No | No | No | Yes |
| [311] | Cuadros-Orellana *et al.* | 2007 | Yes | Yes | No | No | Yes/No^[[44]](#footnote-44)^ |
| [312] | Filée *et al.* | 2007 |  | No | No | No | Yes/No |
| [313] | Fröls *et al.* | 2007 |  | No | No | No | Yes |
| [314] | Fröls *et al.* | 2007 |  | No | No | No | Yes |
| [315] | Geslin *et al.* | 2007 | Yes | Yes^[[45]](#footnote-45)^ | No | No | Yes |
| [316] | Huiskonen & Butcher | 2007 |  | No | No | No | Yes |
| [317] | Iro *et al.* | 2007 | Yes | Yes | No | No | Yes |
| [318] | Keller *et al.* | 2007 |  | No | No | No | Yes |
| [319] | King *et al.* | 2007 |  | No | No | No | Yes |
| [320] | Larson *et al.* | 2007 |  | No | No | No | Yes |
| [321] | Larson *et al.* | 2007 |  | No | No | No | Yes |
| [322] | Lima-Mendez *et al.* | 2007 | Yes | Yes | No | Yes/No^[[46]](#footnote-46)^ | Yes |
| [323] | Mei *et al.* | 2007 | Yes | Yes | Yes | No | No |
| [324] | Merabishvili *et al.* | 2007 |  | No | No | No | Yes |
| [325] | Pagaling *et al.* | 2007 | Yes | Yes | No | No | Yes |
| [326] | Park *et al.* | 2007 | Yes | Yes | No | No | Yes/No |
| [327] | Peng *et al.* | 2007 |  | No | No | No | Yes |
| [328] | Poole & Willerslev | 2007 |  | No | No | No | Yes |
| [329] | Porter *et al.* | 2007 |  | No | No | Yes | Yes |
| [330] | Prangishvili | 2007 |  | No | No | No | Yes |
| [331] | Qureshi *et al.* | 2007 |  | No | No | No | Yes |
| [332] | Russel *et al.* | 2007 |  | No | No | No | Yes |
| [333] | Samuel *et al.* | 2007 |  | No | No | Yes | No |
| [334] | Santos *et al.* | 2007 | Yes | Yes | Yes | No | Yes |
| [335] | Serwer | 2007 |  | No | No | No | Yes |
| [336] | Serwer *et al.* | 2007 |  | No | No | No | Yes |
| [337] | Shinkai *et al.* | 2007 |  | No | No | No | Yes |
| [338] | Snyder *et al.* | 2007 |  | No | No | No | Yes |
| [339] | Stanton | 2007 |  | No | No | Yes/No^[[47]](#footnote-47)^ | Yes/No |
| [340] | Toussaint *et al.* | 2007 |  | Yes/No^[[48]](#footnote-48)^ | No | Yes/No | Yes |
| [341] | Wang *et al.* | 2007 |  | No | No | No | Yes |
| [342] | Zeldovich *et al.* | 2007 | Yes | Yes | No | No | Yes |
| [343] | Zhao *et al.* | 2007 |  | No | No | No | Yes |
| [344] | Abrescia *et al.* | 2008 |  | No | No | No | Yes |
| [345] | Albers & Driessen | 2008 |  | No | No | No | Yes |
| [346] | Anderson *et al.* | 2008 |  | No | No | No | Yes |
| [347] | Andersson | 2008 |  | No | No | No | Yes |
| [348] | Andersson | 2008 |  | No | No | No | Yes |
| [349] | Andersson & Banfield | 2008 |  | No | No | No | Yes |
| [350] | Ash | 2008 |  | No | No | No | Yes^[[49]](#footnote-49)^ |
| [351] | Berkner & Lipps | 2008 |  | No | No | No | Yes |
| [352] | Bize *et al.* | 2008 |  | No | No | No | Yes |
| [353] | Caetano-Anolles *et al.* | 2008 |  | No | No | No | Yes |
| [354] | Comeau *et al.* | 2008 |  | Yes/No^[[50]](#footnote-50)^ | Yes | No | Yes |
| [355] | Donovaro *et al.* | 2008 |  | Yes/No^[[51]](#footnote-51)^ | No | Yes/No^[[52]](#footnote-52)^ | Yes |
| [356] | Filée *et al.* | 2008 |  | No | No | No | Yes |
| [357] | Gutiérrez *et al.* | 2008 |  | No | No | No | Yes |
| [358] | Hatfull | 2008 |  | Yes/No^[[53]](#footnote-53)^ | No | No | Yes |
| [359] | Jäälinoja *et al.* | 2008 |  | No | No | No | Yes |
| [360] | Jaatinen *et al.* | 2008 |  | No | No | No | Yes |
| [361] | Jackson & Jackson | 2008 |  | No | No | No | Yes |
| [362] | Kato *et al.* | 2008 | Yes | No | No | Yes | No |
| [363] | Kivelä et al. | 2008 |  | No | No | No | Yes |
| [364] | Koonin *et al.* | 2008 |  | No | No | No | Yes |
| [365] | Koti *et al.* | 2008 |  | Yes | No | No | No^[[54]](#footnote-54)^ |
| [366] | Krisch & Comeau | 2008 |  | No | No | No | Yes |
| [367] | Krupovič & Bamford | 2008 | Yes | No | No | Yes^[[55]](#footnote-55)^ | Yes |
| [368] | Krupovič & Bamford | 2008 |  | No | No | No | Yes |
| [369] | Kudela *et al.* | 2008 | Yes | Yes | No | No | No |
| [370] | Kyle *et al.* | 2008 | Yes | Yes | No | No | Yes |
| [371] | Kyle *et al.* | 2008 | Yes | Yes | No | No | No |
| [372] | La Scola *et al.* | 2008 |  | No | No | No | Yes |
| [373] | Lindås *et al.* | 2008 |  | No | No | No | Yes |
| [374] | Liu & Zhang | 2008 |  | No | No | No | Yes |
| [375] | Marraffini & Sontheimer | 2008 |  | Yes/No^[[56]](#footnote-56)^ | No | No | No |
| [376] | Martin *et al.* | 2008 | Yes | Yes | No | No | Yes |
| [377] | McGeoch & Bell | 2008 |  | No | No | No | Yes |
| [378] | Menon *et al.* | 2008 |  | No | No | No | Yes |
| [379] | Morier iet al. | 2008 |  | No | No | No | Yes |
| [380] | Ortmann *et al.* | 2008 |  | No | No | No | Yes |
| [381] | Peng | 2008 |  | No | No | No | Yes |
| [382] | Porter & Dyall-Smith | 2008 |  | No | No | No | Yes |
| [383] | Porter *et al.* | 2008 |  | No | No | No | Yes |
| [384] | Prestel *et al.* | 2008 | Yes | Yes | No | No | Yes |
| [385] | Pride & Schoenfeld | 2008 |  | No | No | No | Yes |
| [386] | Randau & Söll | 2008 |  | No | No | No | Yes |
| [387] | Raoult & Forterre | 2008 |  | No | No | No | Yes |
| [388] | Säwström *et al.* | 2008 |  | No | No | No | Yes |
| [389] | Schoenfeld *et al.* | 2008 | Yes | No | No | Yes^[[57]](#footnote-57)^ | Yes |
| [390] | Sinkovics & Horvath | 2008 |  | No | No | No | Yes |
| [391] | Smallridge | 2008 |  | No | No | No | Yes |
| [392] | Soler *et al.* | 2008 |  | No | No | No | Yes |
| [393] | Steinmetz *et al.* | 2008 |  | No | No | No | Yes |
| [394] | Sun & Caetano-Anollés | 2008 |  | No^^[[58]](#footnote-58)^^ | No | No | Yes |
| [395] | Sun & Caetano-Anollés | 2008 |  | No | No | No | Yes |
| [396] | Thurber *et al.* | 2008 |  | No | No | No | Yes |
| [397] | Vestergaard *et al.* | 2008 |  | No | No | No | Yes |
| [398] | Vestergaard *et al.* | 2008 |  | No | No | No | Yes |
| [399] | Winter *et al.* | 2008 |  | No | No | No | Yes |
| [400] | Witzany | 2008 | Yes | Yes | No | No | Yes |
| [401] | Witzany | 2008 |  | Yes | No | No | Yes |
| [402] | Ackermann | 2009 | Yes | Yes^[[59]](#footnote-59)^ | No | No | Yes |
| [403] | Baker *et al.* | 2009 |  | No^[[60]](#footnote-60)^ | No | No | Yes |
| [404] | Banfield & Young | 2009 |  | Yes/No^[[61]](#footnote-61)^ | No | No | Yes |
| [405] | Basta *et al.* | 2009 |  | No | No | No | Yes |
| [406] | Bell | 2009 |  | No | No | No | Yes |
| [407] | Bize | 2009 |  | No | No | No | Yes |
| [408] | Bochman & Schwacha | 2009 | Yes | Yes | No | No | Yes |
| [409] | Brumfield *et al.* | 2009 | Yes | Yes | No | Yes | Yes |
| [410] | Brüssow | 2009 | Yes | Yes | No | No | Yes |
| [411] | Buée *et al.* | 2009 |  | No | No | No | Yes |
| [412] | Byrne *et al.* | 2009 |  | No | No | No | Yes |
| [413] | Cerdeño-Tárraga | 2009 |  | Yes/No^[[62]](#footnote-62)^ | No | No | Yes |
| [414] | Comolli *et al.* | 2009 |  | No | No | No | Yes |
| [415] | Cortez *et al.* | 2009 |  | Yes/No^[[63]](#footnote-63)^ | No | No | Yes |
| [416] | DasSarma *et al.* | 2009 |  | No | No | No | Yes |
| [417] | Dick *et al.* | 2009 | Yes | No | No | Yes^[[64]](#footnote-64)^ | Yes |
| [418] | Díez-Villaseñor *et al.* | 2009 |  | No | No | No | Yes |
| [419] | Ettema & Bernander | 2009 |  | No | No | No | Yes |
| [420] | Evans | 2009 | Yes | No | No | Yes | Yes |
| [421] | Forterre & Prangishvili | 2009 |  | No | No | No | Yes |
| [422] | Forterre & Prangishvili | 2009 |  | No | No | No | Yes |
| [423] | Fujishima *et al.* | 2009 |  | No | No | No | Yes |
| [424] | Fulton *et al.* | 2009 |  | No | No | No | Yes |
| [425] | Goulet *et al.* | 2009 |  | No | No | No | Yes |
| [426] | Goulet *et al.* | 2009 |  | No | No | No | Yes |
| [427] | Goulet *et al.* | 2009 |  | No | No | No | Yes |
| [428] | Guillière et al. | 2009 |  | No | No | No | Yes |
| [429] | Held & Whitaker | 2009 |  | No | No | No | Yes |
| [430] | Hyman & Abedon | 2009 | Yes | Yes | No | No | Yes |
| [431] | Jalasvuori & Bamford | 2009 |  | No^[[65]](#footnote-65)^ | No | No | Yes |
| [432] | Jalasvuori *et al.* | 2009 | Yes | Yes | No | No | Yes |
| [433] | Jalasvuori *et al.* | 2009 |  | No | No | No | Yes |
| [434] | Keller *et al.* | 2009 |  | No | No | No | Yes |
| [435] | Keller *et al.* | 2009 |  | No | No | No | Yes |
| [436] | Koonin | 2009 |  | No^[[66]](#footnote-66)^ | No | No | Yes |
| [437] | Kropinski *et al.* | 2009 | Yes | Yes^[[67]](#footnote-67)^ | No | Yes | Yes |
| [438] | Kukkaro & Bamford | 2009 |  | No | No | No | Yes |
| [439] | Lang *et al.* | 2009 | Yes | Yes | No | No | No |
| [440] | Lavigne *et al.* | 2009 | Yes | Yes | No | No | Yes |
| [441] | Lawrence *et al.* | 2009 |  | No | No | No | Yes |
| [442] | Liu *et al.* | 2009 |  | No | No | Yes/No^[[68]](#footnote-68)^ | Yes |
| [443] | Moreira & López-García | 2009 | Yes | Yes | No | No | Yes |
| [444] | Papke | 2009 | Yes | Yes | No | No | No |
| [445] | Pietilä *et al.* | 2009 |  | No | No | No | Yes |
| [446] | Ravin *et al.* | 2009 |  | No | No | No | Yes |
| [447] | Redder *et al.* | 2009 |  | No | No | No | Yes |
| [448] | Rohwer & Thurber | 2009 | Yes | Yes^[[69]](#footnote-69)^ | No | Yes/No | Yes |
| [449] | Rohwer *et al.* | 2009 |  | No^[[70]](#footnote-70)^ | No | No | Yes |
| [450] | Rosario *et al.* | 2009 |  | No | No | No | Yes |
| [451] | Roucourt & Lavigne | 2009 |  | No | No | No | Yes |
| [452] | Sabet *et al.* | 2009 |  | No | Yes^[[71]](#footnote-71)^ | No | Yes |
| [453] | Sanchez *et al.* | 2009 |  | No | No | No | Yes |
| [454] | Schlenker *et al.* | 2009 |  | No | No | No | Yes |
| [455] | Sevastsyanovich *et al.* | 2009 |  | No | No | No | Yes |
| [456] | Shah *et al.* | 2009 |  | No | No | No | Yes |
| [457] | Sulkowska *et al.* | 2009 |  | No | No | No | Yes |
| [458] | Szymczyna *et al.* | 2009 |  | No | No | No | Yes |
| [459] | Thurber *et al.* | 2009 |  | No | No | No | Yes |
| [460] | Valdivia-Granda & Larson | 2009 |  | No | No | No | Yes |
| [461] | Waddell *et al.* | 2009 | Yes | Yes | No | No | Yes/No |
| [462] | Wilmes *et al.* | 2009 |  | No | No | No | Yes |
| [463] | Witzany | 2009 | Yes | Yes | No | No | Yes |
| [464] | Wu *et al.* | 2009 |  | No | No | No | Yes |
| [465] | Xiao *et al.* | 2009 |  | No | No | No | Yes |
| [466] | Yan *et al.* | 2009 |  | No | No | No | Yes |
| [467] | Yokobori | 2009 |  | No | No | No | Yes |
| [468] | Yutin & Koonin | 2009 |  | No | No | No | Yes |
| [469] | Zivanovic *et al.* | 2009 |  | No | No | No | Yes |
| [470] | Agol *et al.* | 2010 |  | No^[[72]](#footnote-72)^ | No | No | Yes |
| [471] | Bernander & Ettema | 2010 |  | No | No | No | Yes |
| [472] | Bettarel *et al.* | 2010 |  | Yes/No | No | No | Yes |
| [473] | Claverie & Abergel | 2010 | Yes | Yes | No | No | Yes |
| [474] | Contursi *et al.* | 2010 |  | No | No | No | Yes |
| [475] | de Souza *et al.* | 2010 |  | No | No | No | Yes |
| [476] | Deschavanne *et al.* | 2010 |  | No | No | No | Yes |
| [477] | Desnues & Raoult | 2010 |  | No | No | No | Yes |
| [478] | Deveau *et al.* | 2010 |  | No | No | No | Yes |
| [479] | Deza | 2010 |  | No | No | No | Yes |
| [480] | Fard *et al.* | 2010 | Yes | Yes | No | No | No |
| [481] | Fisher *et al.* | 2010 |  | No | No | No | Yes |
| [482] | Flügel | 2010 | Yes | Yes | No | No | Yes |
| [483] | Forterre | 2010 |  | No | No | No | Yes |
| [484] | Forterre | 2010 |  | No | No | No | Yes |
| [485] | Fu *et al.* | 2010 |  | No | No | No | Yes |
| [486] | Garrett *et al.* | 2010 |  | No | No | No | Yes |
| [487] | Goulet *et al.* | 2010 |  | No | No | No | Yes |
| [488] | Goulet *et al.* | 2010 |  | No | No | No | Yes |
| [489] | Grant & Heaphy | 2010 |  | Yes/No^[[73]](#footnote-73)^ | No | No | Yes |
| [490] | Groisillier *et al.* | 2010 |  | No | No | No | Yes |
| [491] | Happonen *et al.* | 2010 |  | No | No | No | Yes |
| [492] | Heinemann *et al.* | 2010 |  | No | No | No | Yes |
| [493] | Inskeep *et al.* | 2010 |  | No | No | No | Yes |
| [494] | Jacquet *et al* | 2010 |  | Yes/No^[[74]](#footnote-74)^ | No | No | Yes |
| [495] | Jalasvuori *et al.* | 2010 |  | Yes/No^[[75]](#footnote-75)^ | No | No | Yes |
| [496] | Karginov & Hannon | 2010 | Yes | Yes | No | Yes^[[76]](#footnote-76)^ | Yes |
| [497] | Khayat *et al.* | 2010 |  | No | No | No | Yes |
| [498] | Klieve | 2010 | Yes | Yes | No | No | Yes |
| [499] | Koonin | 2010 |  | No | No | No | Yes |
| [500] | Koudelka & Manchester | 2010 |  | No | No | No | Yes |
| [501] | Kristensen *et al.* | 2010 |  | Yes/No^[[77]](#footnote-77)^ | No | No | Yes |
| [502] | Krupovič & Bamford | 2010 |  | No | No | No | Yes^[[78]](#footnote-78)^ |
| [503] | Krupovič & Bamford | 2010 |  | No | No | No | Yes |
| [504] | Krupovič *et al.* | 2010 |  | No | No | No | Yes |
| [505] | Krupovič *et al.* | 2010 |  | No | No | No | Yes |
| [506] | Liesegang *et al.* | 2010 | Yes | Yes | No | No | No |
| [507] | Lundin *et al.* | 2010 | Yes | No | Yes | No | Yes |
| [508] | Ma *et al.* | 2010 | Yes | Yes | No | No | Yes |
| [509] | Marraffini & Sontheimer | 2010 | Yes | Yes | No | No | Yes^[[79]](#footnote-79)^ |
| [510] | Marraffini & Sontheimer | 2010 | Yes | Yes | No | No | Yes |
| [511] | Menon *et al.* | 2010 |  | No | No | No | Yes |
| [512] | Mochizuki *et al.* | 2010 |  | No | No | No | Yes |
| [513] | Pietilä *et al.* | 2010 |  | No | No | No | Yes |
| [514] | Quax *et al.* | 2010 |  | No | No | No | Yes |
| [515] | Raoult | 2010 |  | No | No | No | Yes |
| [516] | Raoult & Boyer | 2010 |  | No | No | No | Yes |
| [517] | Roine *et al.* | 2010 |  | No | No | No | Yes |
| [518] | Ruprich-Robert & Thuriaux | 2010 |  | No | No | No | Yes |
| [519] | Santos *et al.* | 2010 | Yes | Yes | Yes | No | Yes |
| [520] | Snyder *et al.* | 2010 |  | No | No | No | Yes |
| [521] | Soler *et al.* | 2010 |  | No | No | No | Yes |
| [522] | Sorokin *et al.* | 2010 |  | No | No | No | Yes |
| [523] | Steinmetz *et al.* | 2010 |  | No | No | No | Yes |
| [524] | Sun *et al.* | 2010 |  | No | No | No | Yes^[[80]](#footnote-80)^ |
| [525] | Tie *et al.* | 2010 |  | No | No | No | Yes |
| [526] | Tremberger *et al.* | 2010 |  | No | No | No | Yes |
| [527] | van Etten *et al.* | 2010 |  | No | No | No | Yes |
| [528] | Villarreal & Witzany | 2010 | Yes | Yes | No | Yes/No^[[81]](#footnote-81)^ | Yes^[[82]](#footnote-82)^ |
| [529] | Waligora *et al.* | 2010 |  | No | No | No | Yes |
| [530] | Walters & Chong | 2010 | Yes | Yes | No | No | No |
| [531] | Walther *et al.* | 2010 |  | Yes/No | No | No | Yes |
| [532] | Wang & Goldenfeld | 2010 |  | No | No | No | Yes |
| [533] | Wei & Zhnag | 2010 |  | No | No | No | Yes |
| [534] | Wilson & Schroeder | 2010 |  | No | No | No | Yes |
| [535] | Winter *et al.* | 2010 |  | No | No | No | Yes |
| [536] | Zaparty *et al.* | 2010 |  | No | No | No | Yes |
| [537] | Zhang *et al.* | 2010 |  | No | No | No | Yes^[[83]](#footnote-83)^ |
| [538] | Zourob & Ripp | 2010 | Yes | Yes | No | No | No |
| [539] | Abroi & Gough | 2011 |  | No | No | No | Yes |
| [540] | Albers & Meyer | 2011 |  | No | No | No | Yes |
| [541] | Anderson *et al.* | 2011 |  | No | No | No | Yes |
| [542] | Bertin *et al.* | 2011 |  | No | No | No | Yes^[[84]](#footnote-84)^ |
| [543] | Bettarel *et al.* | 2011 | Yes | Yes | Yes | Yes | Yes |
| [544] | Blumberg | 2011 |  | No | No | No | Yes |
| [545] | Breitbart | 2011 |  | No | No | No | Yes |
| [546] | Brodt *et al.* | 2011 | Yes | Yes | No | No | Yes |
| [547] | Cavicchioli | 2011 |  | Yes/No^[[85]](#footnote-85)^ | No | No | Yes |
| [548] | Clokie *et al.* | 2011 | Yes | Yes | No | No | Yes |
| [549] | Contursi *et al.* | 2011 |  | No | No | No | Yes |
| [550] | DeYoung *et al.* | 2011 |  | No | No | No | Yes |
| [551] | Dyall-Smith *et al.* | 2011 | Yes | Yes/No | Yes | Yes | Yes |
| [552] | Erdmann *et al.* | 2011 |  | No | No | No | Yes |
| [553] | Forterre | 2011 |  | No | No | No | Yes |
| [554] | Forterre | 2011 |  | No | No | No | Yes |
| [555] | Fu & Johnson | 2011 |  | No | No | No | Yes |
| [556] | Gardner *et al.* | 2011 |  | No | No | No | Yes |
| [557] | Gardner *et al.* | 2011 | Yes | Yes^[[86]](#footnote-86)^ | No | No | Yes |
| [558] | Gill & Brinkman | 2011 |  | No | No | No | Yes |
| [559] | Gonnet *et al.* | 2011 | Yes | Yes | No | No | Yes |
| [560] | Goulet *et al.* | 2011 |  | No | No | No | Yes |
| [561] | Gudbergsdottir *et al.* | 2011 |  | No | No | No | Yes |
| [562] | Heinemann *et al.* | 2011 | Yes | No | No | Yes | Yes |
| [563] | Herrero-Uribe | 2011 |  | No | No | No | Yes |
| [564] | Hirata *et al.* | 2011 |  | No | No | No | Yes |
| [565] | Ishino *et al.* | 2011 |  | No | No | No | Yes |
| [566] | Jarrell *et al.* | 2011 |  | Yes/No^[[87]](#footnote-87)^ | No | No | Yes |
| [567] | Jorda & Yeates | 2011 |  | No | No | No | Yes |
| [568] | Kan *et al.* | 2011 |  | No | No | No | Yes |
| [569] | Kaster *et al.* | 2011 | Yes | Yes | No | Yes | No |
| [570] | Kazlauskas & Venclovas | 2011 |  | No | No | No | Yes |
| [571] | Kristensen *et al.* | 2011 | Yes | Yes | No | Yes/No^[[88]](#footnote-88)^ | Yes |
| [572] | Krupovic & Bamford | 2011 |  | No | No | No | Yes |
| [573] | Krupovic & Cvirkaite-Krupovic | 2011 |  | No | No | No | Yes |
| [574] | Krupovic & Forterre | 2011 |  | No | No | No | Yes |
| [575] | Krupovic *et al.* | 2011 |  | No | No | No | Yes |
| [576] | Krupovic *et al.* | 2011 |  | No | No | No | Yes |
| [577] | Laganeckas *et al.* | 2011 |  | No | No | No | Yes |
| [578] | Litchfield | 2011 | Yes | No | Yes | No | No |
| [579] | Liu *et al.* | 2011 |  | No | No | No | Yes |
| [580] | Makarova *et al.* | 2011 | Yes | Yes^[[89]](#footnote-89)^ | No | Yes | Yes^[[90]](#footnote-90)^ |
| [581] | Manica *et al.* | 2011 |  | No | No | No | Yes |
| [582] | Mochizuki *et al.* | 2011 |  | No | No | No | Yes |
| [583] | Oke *et al.* | 2011 |  | No | No | No | Yes |
| [584] | Pan *et al.* | 2011 |  | No | No | No | Yes |
| [585] | Pina *et al.* | 2011 |  | No | No | No | Yes |
| [586] | Prangishvili | 2011 |  | No | No | No | Yes |
| [587] | Prangishvili & Quax | 2011 |  | No | No | No | Yes |
| [588] | Quax *et al.* | 2011 |  | No | No | No | Yes |
| [589] | Rohwer & Merry | 2011 |  | No | No | No | Yes |
| [590] | Sanmukh *et al.* | 2011 | Yes | Yes | Yes | No | Yes |
| [591] | Santos *et al.* | 2011 | Yes | Yes | Yes | No | Yes |
| [592] | Scheele *et al.* | 2011 |  | No | No | No | Yes |
| [593] | Sejian *et al.* | 2011 | Yes | Yes | No | No | Yes |
| [594] | Serwer | 2011 |  | No | No | No | Yes |
| [595] | Shah & Garrett | 2011 |  | No | No | No | Yes |
| [596] | Shah & Garrett | 2011 |  | No | No | No | Yes |
| [597] | Siebers *et al.* | 2011 |  | No | No | No | Yes |
| [598] | Siering *et al.* | 2011 |  | No | No | No | Yes |
| [599] | Sime-Ngando *et al.* | 2011 | Yes | Yes^[[91]](#footnote-91)^ | Yes/No^[[92]](#footnote-92)^ | No | Yes |
| [600] | Snyder & Young | 2011 |  | No | No | No | Yes |
| [601] | Snyder & Young | 2011 |  | No | No | No | Yes |
| [602] | Snyder *et al.* | 2011 |  | No | No | No | Yes |
| [603] | Soler *et al.* | 2011 |  | No | No | No | Yes |
| [604] | Synder *et al.* | 2011 |  | No | No | No | Yes |
| [605] | Tamakoshi *et al.* | 2011 | Yes | Yes^[[93]](#footnote-93)^ | No | No | Yes |
| [606] | Terns & Terns | 2011 |  | No | No | No | Yes^[[94]](#footnote-94)^ |
| [607] | Thurber & Correa | 2011 |  | No | No | No | Yes |
| [608] | Trojet *et al.* | 2011 |  | No | No | No | Yes |
| [609] | Vesteg & Krajčovič | 2011 |  | No | No | No | Yes |
| [610] | Villarreal | 2011 | Yes | No | No | Yes | Yes |
| [611] | Visweswaran *et al.* | 2011 | Yes | No | No | Yes | No |
| [612] | Visweswaran *et al.* | 2011 | Yes | No | No | Yes | Yes |
| [613] | Wirth *et al.* | 2011 |  | No | No | No | Yes |
| [614] | You *et al.* | 2011 |  | No | No | No | Yes |
| [615] | Aalto *et al.* | 2012 |  | No | No | No | Yes |
| [616] | Abrescia *et al.* | 2012 |  | No | No | No | Yes |
| [617] | Ackermann | 2012 |  | No | No | No | Yes |
| [618] | Ackermann | 2012 |  | No | No | No | Yes |
| [619] | Ackermann & Prangishvili | 2012 | Yes | Yes^[[95]](#footnote-95)^ | No | No | Yes |
| [620] | Atanasova *et al.* | 2012 |  | No | No^[[96]](#footnote-96)^ | No | Yes |
| [621] | Blackwood *et al.* | 2012 |  | No | No | No | Yes^[[97]](#footnote-97)^ |
| [622] | Bolduc *et al.* | 2012 |  | No | No | No | Yes |
| [623] | Borrel *et al.* | 2012 |  | No | No | No | Yes |
| [624] | Boujelben *et al.* | 2012 | Yes | Yes | No | No | Yes |
| [625] | Ceballos *et al.* | 2012 |  | No | No | No | Yes |
| [626] | Chan *et al.* | 2012 |  | No | No | No | Yes |
| [627] | Chen *et al.* | 2012 |  | No | No | No | Yes |
| [628] | Dearborn *et al.* | 2012 |  | No | No | No | Yes |
| [629] | Deng *et al.* | 2012 |  | No | No | No | Yes |
| [630] | Desnues *et al.* | 2012 |  | No | No | No | Yes |
| [631] | Dwivedi *et al.* | 2012 |  | No | No | No | Yes |
| [632] | Eilers *et al.* | 2012 |  | No | No | No | Yes |
| [633] | Emerson | 2012 |  | No | No | No | Yes |
| [634] | Erdmann & Garrett | 2012 |  | No | No | No | Yes |
| [635] | Felisberto-Rodrigues *et al.* | 2012 |  | No | No | No | Yes |
| [636] | Filèe & Chandler | 2012 | Yes | Yes | No | No | Yes |
| [637] | Forterre | 2012 |  | No | No | No | Yes |
| [638] | Forterre | 2012 |  | No | No | No | Yes |
| [639] | Fu & Johnsom | 2012 |  | No | No | No | Yes |
| [640] | Garcia-Heredia | 2012 | Yes | Yes | Yes | No | Yes |
| [641] | Gaudin *et al.* | 2012 |  | No | No | No | Yes |
| [642] | Georgiades & Raoult | 2012 | Yes | Yes | No | No | No |
| [643] | Gophna & Brodt | 2012 |  | No | No | No | Yes |
| [644] | Gorlas *et al.* | 2012 |  | No | No | No | Yes |
| [645] | Hedlund *et al.* | 2012 |  | No | No | No | Yes |
| [646] | Henry & Debarbieux | 2012 |  | No | No | No | Yes |
| [647] | Huang | 2012 |  | No | No | No | Yes |
| [648] | Hyman & Abedon | 2012 | Yes | Yes | No | No | Yes |
| [649] | Iverson & Stedman | 2012 |  | No | No | No | Yes |
| [650] | Jaakkola *et al.* | 2012 |  | No | No | No | Yes |
| [651] | Kandiba *et al.* | 2012 |  | No | No | No | Yes |
| [652] | Klein *et al.* | 2012 |  | No | No | No | Yes |
| [653] | Koonin *et al.* | 2012 |  | No | No | No | Yes |
| [654] | Kristensen *et al.* | 2012 |  | Yes/No^[[98]](#footnote-98)^ | No | No | Yes |
| [655] | Krupovic *et al.* | 2012 |  | No | No | No | Yes |
| [656] | Krupovic *et al.* | 2012 | Yes | Yes | No | No | Yes |
| [657] | Lang *et al.* | 2012 | Yes | Yes^[[99]](#footnote-99)^ | No | No | No |
| [658] | Lim *et al.* | 2012 |  | No | No | No | Yes |
| [659] | Lurie-Weinberger *et al.* | 2012 | Yes | Yes | No | No | Yes |
| [660] | Maaty *et al.* | 2012 |  | No | No | No | Yes |
| [661] | Maaty *et al.* | 2012 |  | No | No | No | Yes |
| [662] | Marchfelder *et al.* | 2012 |  | No | No | No | Yes |
| [663] | Mardanov & Ravin | 2012 |  | No | No | No | Yes |
| [664] | Mentasti *et al.* | 2012 |  | No | No | No | Yes |
| [665] | Mochizuki *et al.* | 2012 |  | No | No | No | Yes |
| [666] | Nasir *et al.* | 2012 |  | No | No | No | Yes |
| [667] | Ng *et al.* | 2012 |  | No | No | No | No |
| [668] | Peixeiro *et al.* | 2012 |  | No | No | No | Yes |
| [669] | Pietilä *et al.* | 2012 |  | No | No | No | Yes |
| [670] | Pietilä *et al.* | 2012 |  | No | No | No | Yes |
| [671] | Plagens *et al.* | 2012 |  | Yes/No^[[100]](#footnote-100)^ | No | No | Yes |
| [672] | Poranen & Bamford | 2012 |  | No | No | No | Yes |
| [673] | Prangishvili & Krupovic | 2012 |  | No | No | No | Yes |
| [674] | Redrejo-Rodríguez *et al.* | 2012 |  | No | No | No | Yes |
| [675] | Rissanen | 2012 |  | No | No | No | Yes |
| [676] | Rohwer & Barott | 2012 |  | No | No | No | Yes |
| [677] | Roine & Bamford | 2012 |  | No | No | No | Yes |
| [678] | Roossinck | 2012 |  | No | No | No | Yes |
| [679] | Rosario *et al.* | 2012 |  | No | No | No | Yes |
| [680] | Santos *et al.* | 2012 | Yes | No | Yes^[[101]](#footnote-101)^ | No | Yes |
| [681] | Schlenker *et al.* | 2012 | Yes | Yes^[[102]](#footnote-102)^ | No | No | Yes |
| [682] | Seguritan *et al.* | 2012 |  | Yes/No^[[103]](#footnote-103)^ | No | No | Yes |
| [683] | Senčilo *et al.* | 2012 |  | No | No | No | Yes |
| [684] | Shen *et al.* | 2012 | Yes | No | Yes | No | Yes |
| [685] | Siddaramappa *et al.* | 2012 | Yes | No | No | Yes | Yes |
| [686] | Snyder *et al.* | 2012 |  | No | No | No | Yes |
| [687] | Sugahara *et al.* | 2012 |  | No | No | No | Yes |
| [688] | Veesler *et al.* | 2012 |  | No | No | No | Yes |
| [689] | Visweswaran *et al.* | 2012 |  | No | No | No | Yes^[[104]](#footnote-104)^ |
| [690] | Weinberger *et al.* | 2012 |  | No | No | No | Yes |
| [691] | Xu *et al.* | 2012 |  | No | No | No | Yes |
| [692] | Zhan *et al.* | 2012 | Yes | No | No | Yes | Yes^[[105]](#footnote-105)^ |
| [693] | Zhang *et al.* | 2012 |  | No | No | No | Yes |
| [694] | Zhang *et al.* | 2012 |  | No | No | No | Yes |

**Table 2: Various Synonyms or Approximate Synonyms to “Archaeal Virus” and their Appearance in the Literature.^[[106]](#footnote-106)^**

| **Year** | **Archaebacterial phage** | **Archaebacterial virus** | **Archaebacteriophage** | **Archaeobacteriophage** | **Archaeal phage** | **Archaeophage** | **Archaeal virus** | **Archaebacterial bacteriophage** | **Euryarchae(ot)al virus** | **Crenarchae(ot)al virus** | **Archaea virus** | **Archaevirus** | **Haloarchaeophage** | **Archaeal halophage** | **Euryarchaeotal phage ^[[107]](#footnote-107)^** | **Haloarchaeal virus** | **Archaephage** | **Archaeavirus** | **Archaeabacteriophage** | **Archaea phage** | **Archeophage** | **Archael virus** | **Archeal virus** | **Archaeovirus** | **Archeovirus** | **Archaeon virus** | **Archae virus** | **Archaean virus** |
| --- | --- | --- | --- | --- | --- | --- | --- | --- | --- | --- | --- | --- | --- | --- | --- | --- | --- | --- | --- | --- | --- | --- | --- | --- | --- | --- | --- | --- |
| **1982** | [8] |  |  |  |  |  |  |  |  |  |  |  |  |  |  |  |  |  |  |  |  |  |  |  |  |  |  |  |
| **1984** | [18], [19] | [13] |  |  |  |  |  |  |  |  |  |  |  |  |  |  |  |  |  |  |  |  |  |  |  |  |  |  |
| **1986** | [27] | [28] |  |  |  |  |  |  |  |  |  |  |  |  |  |  |  |  |  |  |  |  |  |  |  |  |  |  |
| **1988** | [38], [39] | [38], [40] |  |  |  |  |  |  |  |  |  |  |  |  |  |  |  |  |  |  |  |  |  |  |  |  |  |  |
| **1989** |  | [41], [44] | [51] |  |  |  |  |  |  |  |  |  |  |  |  |  |  |  |  |  |  |  |  |  |  |  |  |  |
| **1992** |  | [60], [63] |  | [61] | [62] | [64] |  |  |  |  |  |  |  |  |  |  |  |  |  |  |  |  |  |  |  |  |  |  |
| **1993** |  | [67] |  |  | [69] |  | [69] | [70] |  |  |  |  |  |  |  |  |  |  |  |  |  |  |  |  |  |  |  |  |
| **1994** |  |  |  |  |  | [75] | [76], [80] |  |  |  |  |  |  |  |  |  |  |  |  |  |  |  |  |  |  |  |  |  |
| **1995** |  |  |  |  |  | [81], [85] | [82] |  |  |  |  |  |  |  |  |  |  |  |  |  |  |  |  |  |  |  |  |  |
| **1996** | [87], [89] | [87], [88] |  |  |  |  | [92] |  | [92] |  |  |  |  |  |  |  |  |  |  |  |  |  |  |  |  |  |  |  |
| **1997** |  | [94] |  |  | [97] |  | [97] |  |  |  |  |  |  |  |  |  |  |  |  |  |  |  |  |  |  |  |  |  |
| **1998** | [103] |  |  |  | [105] | [105], [108] | [98], [99], [105], [106], [107], [110] |  |  |  | [104] |  |  |  |  |  |  |  |  |  |  |  |  |  |  |  |  |  |
| **1999** | [126] |  |  |  | [111], [114] |  | [111], [113], [114], [112], [115], [117], [119], [122], [123], [127], [128], [129] |  | [114] | [128] |  | [120] |  |  |  |  |  |  |  |  |  |  |  |  |  |  |  |  |
| **2000** |  |  |  |  | [138] |  | [132], [134], [135], [140] |  |  | [133] |  |  | [133] | [133] | [133] |  |  |  |  |  |  |  |  |  |  |  |  |  |
| **2001** | [141], [148] |  |  |  | [154], [156] | [152] | [142], [143], [144], [147], [149], [150], [155], [156], [157], [158] |  | [156] | [156] |  |  |  |  | [156] |  |  |  |  |  |  |  |  |  |  |  |  |  |
| **2002** |  |  |  |  | [165], [179] |  | [161], [162], [165], [167], [168], [170], [174], [175], [180] |  |  | [176] |  |  | [165] |  |  | [180] | [169] |  |  |  |  |  |  |  |  |  |  |  |
| **2003** |  |  |  |  | [187], [188], [191] |  | [186], [188], [189], [190], [193], [194], [195], [199], [200] |  | [190], [199] | [189], [190] |  |  |  |  |  | [186] |  |  |  |  |  |  |  |  |  |  |  |  |
| **2004** |  |  |  |  | [205], [224] | [205], [215] | [201], [202], [203], [207], [208], [209], [210], [212], [213], [215], [220], [221], [223], [226], [227] |  |  | [211], [212], [213], [217], [218] |  | [208] |  |  |  | [223] |  | [225] | [216] |  |  |  |  |  |  |  |  |  |
| **2005** | [248] |  |  |  | [232], [241], [258], [248] | [244] | [229], [230], [231], [234], [237], [239], [240], [242], [243], [246], [247], [249], [250], [251], [252], [253], [255], [256], [257], [258], [259], [260] |  | [249], [250] | [238], [250], [257], [259], [260] | [254] |  |  |  |  | [249], [250] |  |  |  | [248] |  |  |  |  |  |  |  |  |
| **2006** |  |  |  |  | [265], [286], [300], [301] | [288] | [264], [266], [267], [268], [271], [273], [274], [275], [276], [278], [279], [281], [282], [284], [285], [289], [290], [291], [292], [293], [297] |  | [275], [293] | [274], [275], [277], [279], [290], [292], [293] | [285] |  |  |  |  | [282], [298] |  |  |  |  | [296] | [294] | [295] |  |  |  |  |  |
| **2007** |  |  |  |  | [302], [323] | [326] | [302], [303], [304], [308], [309], [310], [314], [315], [316], [317], [318], [320], [321], [322], [324], [325], [329], [332], [335], [336], [338], [343] |  | [315], [327] | [308], [310], [315], [318], [320], [321], [327], [337], [342] |  | [303], [340] |  |  |  | [325], [327] |  |  |  |  |  |  | [324] |  |  |  |  |  |
| **2008** | [369] | [390] |  |  | [365], [400], [401] | [384] | [344], [347], [349], [352], [353], [354], [355], [356], [358], [359], [360], [361], [363], [364], [366], [367], [368], [370], [372], [373], [376], [377], [378], [379], [380], [382], [383], [385], [386], [387], [388], [389], [391], [393], [394], [395], [394], [396], [398], [399] |  | [359], [368], [389] | [368], [378], [380], [381], [389], [397], [398] |  |  |  |  |  | [367], [382] |  |  |  |  |  |  |  | [387], [392] |  |  |  |  |
| **2009** |  |  |  |  | [430], [463], [439] | [461] | [402], [403], [405], [407], [408], [409], [410], [411], [412], [416], [417], [418], [419], [420], [421], [422], [423], [424], [425], [426], [427], [428], [430], [432], [434], [436], [437], [438], [441], [445], [446], [447], [448], [449], [450], [456], [458], [459], [460], [462], [464], [467], [468] |  | [409], [432], [433], [441], [466] | [405], [407], [409], [413], [423], [428], [431], [433], [441], [453], [454], [456], [457] | [410], [442] |  |  |  |  | [432] | [408] |  |  |  |  | [455] | [440], [451] | [422] | [422] | [442] |  |  |
| **2010** | [473] |  |  |  | [480], [528] | [498] | [471], [472], [473], [475], [476], [478], [481], [482], [483], [484], [485], [486], [487], [488], [490], [491], [492], [494], [497], [498], [499], [500], [503], [504], [505], [507], [508], [511], [512], [513], [514], [516], [517], [518], [520], [521], [522], [523], [526], [532], [533], [535] |  | [486], [491] | [486], [487], [491], [493], [511], [512], [517] | [525], [527] |  |  |  |  | [486], [505], [513], [517] | [538] |  |  |  |  |  | [479] | [483], [484] | [472] |  | [515], [477] | [470] |
| **2011** |  |  |  |  |  | [559], [571] | [539], [540], [541], [543], [545], [546], [547], [548], [552], [553], [554], [557], [558], [560], [562], [563], [565], [566], [570], [572], [574], [575], [576], [577], [582], [583], [584], [585], [586], [587], [588], [589], [590], [591], [593], [594], [595], [597], [599], [600], [601], [602], [603], [604], [605], [607], [608], [609], [610], [613], [614] |  | [575], [576], [582], [613] | [549], [559], [562], [576], [582], [587], [592] [595], [596], [599], [600], [601], [602], [613] |  |  | [543] |  |  | [543], [547], [585], [599] |  |  |  |  |  |  |  | [575], [585] | [585] |  | [544] |  |
| **2012** |  | [674] |  |  | [636] | [642] | [616], [617], [618], [619], [620], [622], [623], [625], [626], [628], [630], [631], [632], [633], [634], [636], [637], [639], [641], [643], [644], [645], [646], [647], [648], [649], [650], [652], [653], [654], [655], [656], [658], [660], [661], [662], [663], [664], [665], [668], [669], [670], [671], [673], [675], [676], [677], [678], [679], [680], [681], [682], [683], [684], [686], [687], [691], [693], [694] |  | [620], [644], [668], [669], [677], [683], [691] | [619], [620], [625], [629], [632], [634], [635], [644], [660], [661], [668], [669], [670], [677], [681], [686], [691] | [652], [676] |  |  |  |  | [620], [644], [650], [651], [652], [665], [669], [670], [677], [680], [683], [691] |  |  |  |  |  |  | [672], [667], [688] | [638]. [656], [659] | [619], [640], [644], [665] |  |  |  |
| Totals | 15 | 14 | 1 | 1 | 35 | 18 | 371 | 1 | 32 | 90 | 9 | 4 | 3 | 1 | 2 | 32 | 3 | 1 | 1 | 1 | 1 | 2 | 8 | 11 | 7 | 1 | 3 | 1 |

References

[1] T. Torsvik and I. D. Dundas, "Bacteriophage of *Halobacterium salinarium*," *Nature*, vol. 248, no. 5450, pp. 680-681, 1974.

[2] A. C. Wais, M. Kon, R. E. MacDonald, and B. D. Stollar, "Salt-dependent bacteriophage infecting *Halobacterium cutirubrum* and *H. halobium*," *Nature*, vol. 256, no. 5515, pp. 314-315, 1975.

[3] J. C. Stube, F. J. Post, and D. B. Porcella, *Nitrogen cycling in microcosms and application to the biology of the northern arm of the Great Salt Lake*, Utah Water Research Laboratory, 1976.

[4] T. Torsvik and I. D. Dundas, "Halophilic phage specific for *Halobacterium salinarium* str. 1," in *Energetics and Structure of Halophilic Microorganisms*, S. R. Caplan and M. Ginzburg, Eds. pp. 609-614, Elsevier/North Holland, 1978.

[5] T. Torsvik and I. D. Dundas, "Persisting phage infection in *Halobacterium salinarium* str. 1," *Journal of General Virology*, vol. 47, no. 1, pp. 29-36, 1980.

[6] C. Pauling, "Bacteriophages of *Halobacterium* *halobium*: isolated from fermented fish sauce and primary characterization," *Canadian Journal of Microbiology*, vol. 28, no. 8, pp. 916-921, 1982.

[7] D. C. Reanney and H.-W. Ackermann, "Comparative biology and evolution of bacteriophages," *Advances in Virus Research*, vol. 27, pp. 205-280, 1982.

[8] H. Schnabel, W. Zillig, M. Pfaffle, R. Schnabel, H. Michel, and H. Delius, "*Halobacterium halobium* phage *φ*H," *EMBO Journal*, vol. 1, no. 1, pp. 87-92, 1982.

[9] H. Schnabel, E. Schramm, R. Schnabel, and W. Zillig, "Structrual variability in the genome of phage φH of *Halobacterium halobium*," *Molecular and General Genetics*, vol. 188, no. 3, pp. 370-377, 1982.

[10] S. Yeats, P. McWilliam, and W. Zillig, "A plasmid in the archaebacterium *Sulfolobus acidocaldarius*," *EMBO Journal*, vol. 1, no. 9, pp. 1035-1038, 1982.

[11] D. Janekovic, S. Wunderl, I. Holz, W. Zillig, A. Gierl, and H. Neumann, "TTV1, TTV2 and TTV3, a family of viruses of the extremely thermophilic, anaerobic, sulfur reducing archaebacterium, *Thermoproteus tenax*," *Molecular and General Genetics*, vol. 192, no. 1-2, pp. 39-45, 1983.

[12] G. F. Rohrmann, R. Cheney, and C. Pauling, "Bacteriophages of *Halobacterium halobium*: virion DNAs and proteins," *Canadian Journal of Microbiology*, vol. 29, no. 5, pp. 627-629, 1983.

[13] T. Barbeyron, K. Kean, and P. Forterre, "DNA adenine methylation of GATC sequences appeared recently in the *Escherichia coli* lineage," *Journal of Bacteriology*, vol. 160, no. 2, pp. 586-590, 1984.

[14] L. L. Daniels and A. C. Wais, "Restriction and modification of halophage S45 in *Halobacterium*," *Current Microbiology*, vol. 10, no. 3, pp. 133-136, 1984.

[15] K. F. Jarrell and G. D. Sprott, "Formation and regeneration of *Halobacterium* Spheroplasts," *Current Microbiology*, vol. 10, no. 3, pp. 147-152, 1984.

[16] A. Martin, S. Yeats, D. Janekovic, W. D. Reiter, W. Aicher, and W. Zillig, "SAV 1, a temperate u.v.-inducible DNA virus-like particle from the archaebacterium *Sulfolobus acidocaldarius* isolate B12," *EMBO Journal*, vol. 3, no. 9, pp. 2165-2168, 1984.

[17] H. Schnabel, "Integration of plasmid pΦHL into phage genomes during infection of *Halobacterium halobium* R_1_-L with phage ΦHL1," *Molecular and General Genetics*, vol. 197, no. 1, pp. 19-23, 1984.

[18] H. Schnabel, "An immune strain of *Halobacterium halobium* carries the invertible L segment of phage ΦH as a plasmid," *Proceedings of the National Academy of Sciences, USA*, vol. 81, no. 4, pp. 1017-1020, 1984.

[19] H. Schnabel and W. Zillig, "Circular structure of the genome of phage φH in a lysogenic *Halobacterium halobium*," *Molecular and General Genetics*, vol. 193, no. 3, pp. 422-426, 1984.

[20] H. Schnabel, R. Schnabel, S. Yeats, J. Tu, A. Gierl, H. Neumann, and W. Zillig, "Genome organization and transcription in archaebacteria," *Folia Biologica (Praha)*, vol. 30 Spec. Pub., pp. 2-6, 1984.

[21] N. H. Patterson and C. Pauling, "Evidence for two restriction-modification systems in *Halobacterium cutirubrum*," *Journal of Bacteriology*, vol. 163, no. 2, pp. 783-784, 1985.

[22] D. A. Prangishvili, R. P. Vashakidze, M. G. Chelidze, and I. Y. Gabriadze, "A restriction endonuclease SuaI from the thermoacidophilic archaebacterium *Sulfolobus acidocaldarius*," *FEBS Letters*, vol. 192, no. 1, pp. 57-60, 1985.

[23] A. C. Wais and L. L. Daniels, "Populations of bacteriophage infecting *Halobacterium* in a transient brine pool," *FEMS Microbiology Ecology*, vol. 31, no. 6, pp. 323-326, 1985.

[24] W. Zillig, R. Schnabel, and K. O. Stetter, "Archaebacteria and the origin of the eukaryotic cytoplasm," *Current Topics in Microbiology and Immunology*, vol. 114, pp. 1-18, 1985.

[25] G. Bertani and L. Baresi, "Looking for gene transfer mechanism in methanogenic bacteria," in *Archaebacteria '85*, O. Kandler and W. Zillig, Eds. p. 398, Gustav Fischer Verlag, Stuttgart, 1986.

[26] M. Nadal, G. Mirambeau, P. Forterre, W.-D. Reiter, and M. Duguet, "Positively supercoiled DNA in a virus-like particle of an archaebacterium," *Nature*, vol. 321, no. 6067, pp. 256-258, 1986.

[27] H. Vogelsang-Wenke and D. Oesterhelt, "Halophage φ N," in *Archaebacteria '85*, O. Kandler and W. Zillig, Eds. pp. 403-405, Gustav Fischer Verlag, Stuttgart, 1986.

[28] W. Zillig, F. Gropp, A. Henschen, H. Neumann, P. Palm, W.-D. Reiter, M. Rettenberger, H. Schnabel, and S. Yeats, "Archaebacterial virus-host systems," *Systematic and Applied Microbiology*, vol. 7, no. 1, pp. 58-66, 1986.

[29] W. Zillig, Yeats.S., I. Holz, A. Böck, M. Rettenberger, F. Gropp, and G. Simon, "*DesuIfuroIabus ambivaIens,* gen. nov., sp. nov., an autotrophic archaebacterium facultatively oxidizing or reducing sulfur," *Systematic and Applied Microbiology*, vol. 8, no. 3, pp. 197-203, 1986.

[30] S. W. Cline and W. F. Doolittle, "Efficient transfection of the archaebacterium *Halobacterium halobium*," *Journal of Bacteriology*, vol. 169, no. 3, pp. 1341-1344, 1987.

[31] K. Ebert, C. Hanke, H. Delius, W. Goebel, and F. Pfeifer, "A new insertion element, ISH26, from *Halobacterium halobium*," *Molecular and General Genetics*, vol. 206, no. 1, pp. 81-87, 1987.

[32] W. D. Reiter, P. Palm, W. Voos, J. Kaniecki, B. Grampp, W. Schulz, and W. Zillig, "Putative promoter elements for the ribosomal RNA genes of the thermoacidophilic archaebacterium *Sulfolobus* sp. strain B12," *Nucleic Acids Research*, vol. 15, no. 14, pp. 5581-5595, 1987.

[33] W. D. Reiter, P. Palm, S. Yeats, and W. Zillig, "Gene expression in archaebacteria: physical mapping of constitutive and UV-inducible transcripts from the *Sulfolobus* virus-like particle SSV1," *Molecular and General Genetics*, vol. 209, no. 2, pp. 270-275, 1987.

[34] W.-D. Reiter, P. Palm, A. Henschen, F. Lottspeich, W. Zillig, and B. Grampp, "Identification and characterization of the genes encoding three structural proteins of the *Sulfolobus* virus-like particle SSV1," *Molecular and General Genetics*, vol. 206, no. 1, pp. 144-153, 1987.

[35] S. Sonea, "Bacterial viruses, prophages, and plasmids, reconsidered," *Annals of the New York Academy of Sciences*, vol. 503, pp. 251-260, 1987.

[36] C. R. Woese, "Bacterial evolution," *Microbiological Reviews*, vol. 51, no. 2, pp. 221-271, 1987.

[37] W. Zillig, I. Holz, H.-P. Klenk, J. Trent, S. Wunderl, D. Janekovic, E. Insel, and B. Haas, "*Pyrococcus woesei* sp. nov., an ultra-thermophilic marine archaebacterium, representing a novel order, *Thermococcales*," *Systematic and Applied Microbiology*, vol. 9, no. 1-2, pp. 62-70, 1987.

[38] W.-D. Reiter, W. Zillig, and P. Palm, "Archaebacterial viruses," *Advances in Virus Research*, vol. 34, pp. 143-188, 1988.

[39] H. Vogelsang-Wenke and D. Oesterhelt, "Isolation of a halobacterial phage with a fully cytosine-methylated genome," *Molecular and General Genetics*, vol. 211, no. 3, pp. 407-414, 1988.

[40] W. Zillig, W.-D. Reiter, P. Palm, F. Gropp, H. Neumann, and M. Rettenberger, "Viruses of archaebacteria," in *The Bacteriophages. Volume 1*, R. Calendar, Ed., pp. 517-558, Plenum Press, New York, 1988.

[41] J. W. Brown, C. J. Daniels, and J. N. Reeve, "Gene structure, organization, and expression in archaebacteria," *Critical Reviews in Microbiology*, vol. 16, no. 4, pp. 287-338, 1989.

[42] S. W. Cline, W. L. Lam, R. L. Charlebois, L. C. Schalkwyk, and W. F. Doolittle, "Transformation methods for halophilic archaebacteria," *Canadian Journal of Microbiology*, vol. 35, no. 1, pp. 148-152, 1989.

[43] R. Gropp and D. Oeterhelt, "In vitro translation of halobacterial mRNA," *FEBS Letters*, vol. 259, no. 1, pp. 5-9, 1989.

[44] F. Gropp, P. Palm, and W. Zillig, "Expression and regulation of *Halobacterium halobium* phage ΦH genes," *Canadian Journal of Microbiology*, vol. 35, no. 1, pp. 182-188, 1989.

[45] N. R. Hackett and S. DasSarma, "Characterization of the small endogenous plasmid of *Halobacterium* strain SB3 and its use in transformation of *H. halobium*," *Canadian Journal of Microbiology*, vol. 35, no. 1, pp. 86-91, 1989.

[46] M. Jordan, L. Meile, and T. Leisinger, "Organization of *Methanobacterium thermoautotrophicum* bacteriophage *Ψ* M1 DNA," *Molecular and General Genetics*, vol. 220, no. 1, pp. 161-164, 1989.

[47] L. Meile, U. Jenal, D. Studer, M. Jordan, and T. Leisinger, "Characterization of *Ψ*M1, a virulent phage of *Methanobacterium thermoautotrophicum* Marburg," *Archives of Microbiology*, vol. 152, no. 2, pp. 105-110, 1989.

[48] W. D. Reiter, P. Palm, and S. Yeats, "Transfer RNA genes frequently serve as integration sites for prokaryotic genetic elements," *Nucleic Acids Research*, vol. 17, no. 5, pp. 1907-1914, 1989.

[49] N. L. Schauer and W. B. Whiman, "Formate growth and pH control by volatile formic and acetic acids in batch cultures of methanococci," *Journal of Microbiological Methods*, vol. 10, no. 1, pp. 1-7, 1989.

[50] A. G. Wood, W. B. Whiman, and J. Konisky, "Isolation and characterization of an archaebacterial viruslike particle from *Methanococcus voltae* A3," *Journal of Bacteriology*, vol. 171, no. 1, pp. 93-98, 1989.

[51] L. Wünsche, "Importance of bacteriohphages in fermentation processes," *Acta Biotechnologica*, vol. 9, no. 5, pp. 395-419, 1989.

[52] L. L. Daniels and A. C. Wais, "Ecophysiology of bacteriophage S5100 infecting *Halobacterium cutirubrum*," *Applied and Environmental Microbiology*, vol. 56, no. 11, pp. 3605-3608, 1990.

[53] G. Juez, F. Rodriguez-Valera, N. Herrero, and F. J. Mojica, "Evidence for salt-associated restriction pattern modifications in the archaeobacterium *Haloferax mediterranei*," *Journal of Bacteriology*, vol. 172, no. 12, pp. 7278-7281, 1990.

[54] L. Meile, P. Abendschein, and T. Leisinger, "Transduction in the archaebacterium *Methanobacterium thermoautotrophicum* Marburg," *Journal of Bacteriology*, vol. 172, no. 6, pp. 3507-3508, 1990.

[55] U. Hudepohl, F. Gropp, M. Horne, and W. Zillig, "Heterologous in vitro transcription from two archaebacterial promoters," *FEBS Letters*, vol. 285, no. 2, pp. 257-259, 1991.

[56] R. Ken and N. R. Hackett, "*Halobacterium halobium* strains lysogenic for phage ΦH contain a protein resembling coliphage repressors," *Journal of Bacteriology*, vol. 173, no. 3, pp. 955-960, 1991.

[57] M. P. Krebs, T. Hauss, M. P. Heyn, U. L. RajBhandary, and H. G. Khorana, "Expression of the bacterioopsin gene in *Halobacterium halobium* using a multicopy plasmid," *Proceedings of the National Academy of Sciences, USA*, vol. 88, no. 3, pp. 859-863, 1991.

[58] J. Nölling, M. Frijlink, and W. M. de Vos, "Isolation and characterization of plasmids from different strains of *Methanobacterium thermoformicicum*," *Journal of General Microbiology*, vol. 137, no. 8, pp. 1981-1986, 1991.

[59] P. Palm, C. Schleper, B. Grampp, S. Yeats, P. McWilliam, W. D. Reiter, and W. Zillig, "Complete nucleotide sequence of the virus SSV1 of the archaebacterium *Sulfolobus shibatae*," *Virology*, vol. 185, no. 1, pp. 242-250, 1991.

[60] A. M. Campbell, "Chromosomal insertion sites for phages and plasmids," *Journal of Bacteriology*, vol. 174, no. 23, pp. 7495-7499, 1992.

[61] F. Charbonnier, G. Erauso, T. Barbeyron, D. Prieur, and P. Forterre, "Evidence that a plasmid from a hyperthermophilic archaebacterium is relaxed at physiological temperatures," *Journal of Bacteriology*, vol. 174, no. 19, pp. 6103-6108, 1992.

[62] F. Gropp, B. Grampp, P. Stolt, P. Palm, and W. Zillig, "The immunity-conferring plasmid p*φ*HL from the *Halobacterium salinarium* phage φH: nucleotide sequence and transcription," *Virology*, vol. 190, no. 1, pp. 45-54, 1992.

[63] E. V. Koonin, "Archaebacterial virus SSV1 encodes a putative DnaA-like protein," *Nucleic Acids Research*, vol. 20, no. 5, p. 1143, 1992.

[64] J. N. Reeve, "Molecular biology of methanogens," *Annual Review of Microbiology*, vol. 46, pp. 165-191, 1992.

[65] C. Schleper, K. Kubo, and W. Zillig, "The particle SSV1 from the extremely thermophilic archaeon *Sulfolobus* is a virus: demonstration of infectivity and of transfection with viral DNA," *Proceedings of the National Academy of Sciences, USA*, vol. 89, no. 16, pp. 7645-7649, 1992.

[66] P. Stolt and W. Zillig, "In vivo studies on the effects of immunity genes on early lytic transcription in the *Halobacterium salinarium* phage *φ* H," *Molecular and General Genetics*, vol. 235, no. 2-3, pp. 197-204, 1992.

[67] E. V. Koonin and T. V. Ilyina, "Computer-assisted dissection of rolling circle DNA replication," *Biosystems*, vol. 30, no. 1-3, pp. 241-268, 1993.

[68] M. P. Krebs, R. Mollaaghababa, and H. G. Khorana, "Gene replacement in *Halobacterium halobium* and expression of bacteriorhodopsin mutants," *Proceedings of the National Academy of Sciences, USA*, vol. 90, no. 5, pp. 1987-1991, 1993.

[69] J. Nölling, A. Groffen, and W. M. de Vos, "ΦF1 and ΦF3, two virulent, archaeal phages infecting different thermophilic strains of genus *Methanobacterium*," *Journal of General Microbiology*, vol. 139, no. 10, pp. 2511-2516, 1993.

[70] S. D. Nuttall and M. L. Dyall-Smith, "HF1 and HF2: Novel bacteriophages of halophilic archaea," *Virology*, vol. 197, no. 2, pp. 678-684, 1993.

[71] S. D. Nuttall and M. L. Dyall-Smith, "Ch2, a novel halophilic archaeon from an Australian solar saltern," *International Journal of Systematic Bacteriology*, vol. 43, no. 4, pp. 729-734, 1993.

[72] P. Stolt and W. Zillig, "Antisense RNA mediates transcriptional processing in an archaebacterium, indicating a novel kind of RNase activity," *Molecular Microbiology*, vol. 7, no. 6, pp. 875-882, 1993.

[73] P. Stolt and W. Zillig, "Structure specific ds/ss-RNase activity in the extreme halophile *Halobacterium salinarium*," *Nucleic Acids Research*, vol. 21, no. 24, pp. 5595-5599, 1993.

[74] P. Stolt and W. Zillig, "*In vivo* and *in vitro* analysis of transcription of the L region from the *Halobacterium salinarium* phage *φ*H: definition of a repressor-enhancing gene," *Virology*, vol. 195, no. 2, pp. 649-658, 1993.

[75] F. Charbonnier and P. Forterre, "Comparison of plasmid DNA topology among mesophilic and thermophilic eubacteria and archaebacteria," *Journal of Bacteriology*, vol. 176, no. 5, pp. 1251-1259, 1994.

[76] A. Lehmacher and H. P. Klenk, "Characterization and phylogeny of *mcr*II, a gene cluster encoding an isoenzyme of methyl coenzyme M reductase from hyperthermophilic *Methanothermus fervidus*," *Molecular and General Genetics*, vol. 243, no. 2, pp. 198-206, 1994.

[77] A. Oren, "The ecology of the extremely halophilic archaea," *FEMS Microbiology Reviews*, vol. 13, pp. 415-440, 1994.

[78] P. Stolt and W. Zillig, "Transcription of the halophage *Φ*H repressor gene is abolished by transcription from an inversely oriented lytic promoter," *FEBS Letters*, vol. 344, no. 2-3, pp. 125-128, 1994.

[79] P. Stolt, B. Grampp, and W. Zillig, "Genes for DNA cytosine methyltransferases and structural proteins, expressed during lytic growth by the phage ΦH of the archaebacterium *Halobacterium salinarium*," *Biological Chemistry Hoppe Seyler*, vol. 375, no. 11, pp. 747-757, 1994.

[80] W. Zillig, A. Kletzin, C. Schleper, I. Holz, D. Janekovic, J. Hain, M. Lanzendoerfer, and J. K. Kristjansson, "Screening for *Sulfolobales*, their plasmids and their viruses in Icelandic solfataras," *Systematic and Applied Microbiology*, vol. 16, no. 4, pp. 609-628, 1994.

[81] A. Bouyoub, G. Barbier, J. Querellou, and P. Forterre, "A putative SOS repair gene (*dinF*-like) in a hyperthermophilic archaeon," *Gene*, vol. 167, no. 1-2, pp. 147-149, 1995.

[82] M. V. Cherrier, V. A. Kostyuchenko, C. Xiao, V. D. Bowman, A. J. Battisti, X. Yan, P. R. Chipman, T. S. Baker, J. L. van Etten, and M. G. Rossmann, "An icosahedral algal virus has a complex unique vertex decorated by a spike," *Proceedings of the National Academy of Sciences, USA*, vol. 106, no. 27, pp. 11085-11089, 2009.

[83] M. L. Holmes, F. Pfeifer, and M. L. Dyall-Smith, "Analysis of the halobacterial plasmid pHK2 minimal replicon," *Gene*, vol. 153, no. 1, pp. 117-121, 1995.

[84] S. D. Nuttall and M. L. Dyall-Smith, "Halophage HF2: genome organisation and replication strategy," *Journal of Virology*, vol. 69, no. 4, pp. 2322-2327, 1995.

[85] R. Stettler, C. Thurner, D. Stax, L. Meile, and T. Leisinger, "Evidence for a defective prophage on the chromosome of *Methanobacterium wolfei*," *FEMS Microbiology Letters*, vol. 132, no. 1-2, pp. 85-89, 1995.

[86] C. Aagaard, I. Leviev, R. N. Aravalli, P. Forterre, D. Prieur, and R. A. Garrett, "General vectors for archaeal hyperthermophiles: strategies based on a mobile intron and a plasmid," *FEMS Microbiology Reviews*, vol. 18, no. 2-3, pp. 93-104, 1996.

[87] H.-W. Ackermann, "Frequency of morphological phage descriptions in 1995," *Archives of Virology*, vol. 141, no. 2, pp. 209-218, 1996.

[88] G. Erauso, S. Marsin, N. Benbouzid-Rollet, M. F. Baucher, T. Barbeyron, Y. Zivanovic, D. Prieur, and P. Forterre, "Sequence of plasmid pGT5 from the archaeon *Pyrococcus abyssi*: evidence for rolling-circle replication in a hyperthermophile," *Journal of Bacteriology*, vol. 178, no. 11, pp. 3232-3237, 1996.

[89] U. Goel, T. Kauri, H.-W. Ackermann, and D. J. Kushner, "A moderately halophilic Vibrio from a Spanish saltern and its lytic bacteriophage," *Canadian Journal of Microbiology*, vol. 42, no. 10, pp. 1015-1023, 1996.

[90] N. Guixa-Boixareu, J. I. Calderon-Paz, M. Heldal, G. Bratbak, and C. Pedros-Alio, "Viral lysis and bacterivory as prokaryotic loss factors along a salinity gradient," *Aquatic Microbial Ecology*, vol. 11, no. 3, pp. 215-227, 1996.

[91] C. J. Newbold, K. Ushida, B. Morvan, G. Fonty, and J. P. Jouany, "The role of ciliate protozoa in the lysis of methanogenic archaea in rumen fluid," *Letters in Applied Microbiology*, vol. 23, no. 6, pp. 421-425, 1996.

[92] W. Zillig, D. Prangishvili, C. Schleper, M. Elferink, I. Holz, S. Albers, D. Janekovic, and D. Gotz, "Viruses, plasmids and other genetic elements of thermophilic and hyperthermophilic *Archaea*," *FEMS Microbiology Reviews*, vol. 18, no. 2-3, pp. 225-236, 1996.

[93] H. X. Chiura, "Generalized gene transfer by virus-like particles from marine bacteria," *Aquatic Microbial Ecology*, vol. 13, pp. 75-83, 1997.

[94] D. Esposito and J. J. Scocca, "The integrase family of tyrosine recombinases: evolution of a conserved active site domain," *Nucleic Acids Research*, vol. 25, no. 18, pp. 3605-3614, 1997.

[95] A. Oren, G. Bratbak, and M. Heldal, "Occurrence of virus-like particles in the Dead Sea," *Extremophiles*, vol. 1, no. 3, pp. 143-149, 1997.

[96] D. L. Tumbula, T. L. Bowen, and W. B. Whitman, "Characterization of pURB500 from the archaeon *Methanococcus maripaludis* and construction of a shuttle vector," *Journal of Bacteriology*, vol. 179, no. 9, pp. 2976-2986, 1997.

[97] A. Witte, U. Baranyi, R. Klein, M. Sulzner, C. Luo, G. Wanner, D. H. Krueger, and W. Lubitz, "Characterization of *Natronobacterium magadii* phage ΦCh1, a unique archaeal phage containing DNA and RNA," *Molecular Microbiology*, vol. 23, no. 3, pp. 603-616, 1997.

[98] C. Bath and M. L. Dyall-Smith, "His1, an archaeal virus of the *Fuselloviridae* family that infects *Haloarcula hispanica*," *Journal of Virology*, vol. 72, no. 11, pp. 9392-9395, 1998.

[99] R. Bernander, "Archaea and the cell cycle," *Molecular Microbiology*, vol. 29, no. 4, pp. 955-961, 1998.

[100] R. Cannio, P. Contursi, M. Rossi, and S. Bartolucci, "An autonomously replicating transforming vector for *Sulfolobus solfataricus*," *Journal of Bacteriology*, vol. 180, no. 12, pp. 3237-3240, 1998.

[101] R. L. Charlebois, Q. She, D. P. Sprott, C. W. Sensen, and R. A. Garrett, "*Sulfolobus* genome: from genomics to biology," *Current Opinion in Microbiology*, vol. 1, no. 5, pp. 584-588, 1998.

[102] L. L. Daniels and A. C. Wais, "Virulence of phage populations infecting *Halobacterium* *cutirubrum*," *FEMS Microbiology Ecology*, vol. 25, no. 2, pp. 129-134, 1998.

[103] J. Höök-Nikanne, D. E. Berg, R. M. Peek, Jr., D. Kersulyte, M. K. Tummuru, and M. J. Blaser, "DNA sequence conservation and diversity in transposable element IS605 of Helicobacter pylori," *Helicobacter*, vol. 3, no. 2, pp. 79-85, 1998.

[104] J. Maniloff and H.-W. Ackermann, "Taxonomy of bacterial viruses: establishment of tailed virus genera and the order *Caudovirales*," *Archives in Virology*, vol. 143, no. 10, pp. 2051-2063, 1998.

[105] P. Pfister, A. Wasserfallen, R. Stettler, and T. Leisinger, "Molecular analysis of *Methanobacterium* phage ΨM2," *Molecular Microbiology*, vol. 30, no. 2, pp. 233-244, 1998.

[106] D. Prangishvili, H. P. Klenk, G. Jakobs, A. Schmiechen, C. Hanselmann, I. Holz, and W. Zillig, "Biochemical and phylogenetic characterization of the dUTPase from the archaeal virus SIRV," *Journal of Biological Chemistry*, vol. 273, no. 11, pp. 6024-6029, 1998.

[107] D. Salmi, V. Magrini, P. L. Hartzell, and P. Youderian, "Genetic determinants of immunity and integration of temperate *Myxococcus xanthus* phage Mx8," *Journal of Bacteriology*, vol. 180, no. 3, pp. 614-621, 1998.

[108] J. van der Oost, M. Ciaramella, M. Moracci, F. M. Pisani, M. Rossi, and W. M. de Vos, "Molecular biology of hyperthermophilic *Archaea*," *Advances in Biochemical Engineering/Biotechnology*, vol. 61, pp. 87-115, 1998.

[109] A. Ventosa, J. J. Nieto, and A. Oren, "Biology of moderately halophilic aerobic bacteria," *Microbiology and Molecular Biology Reviews*, vol. 62, no. 2, pp. 504-544, 1998.

[110] W. Zillig, H. P. Arnold, I. Holz, D. Prangishvili, A. Schweier, K. Stedman, Q. She, H. Phan, R. Garrett, and J. K. Kristjansson, "Genetic elements in the extremely thermophilic archaeon *Sulfolobus*," *Extremophiles*, vol. 2, no. 3, pp. 131-140, 1998.

[111] H.-W. Ackermann, "Tailed bacteriophages: the order *Caudovirales*," *Advances in Virus Research*, vol. 51, pp. 135-201, 1999.

[112] C. M. M. C. Andrade, N. Pereira, Jr., and G. Antranikian, "Extremely thermophililc microorganisms and their polymer-hydrolytic enzymes," *Revista de Microbiologia*, vol. 30, pp. 287-298, 1999.

[113] H. P. Arnold, Q. She, H. Phan, K. Stedman, D. Prangishvili, I. Holz, J. K. Kristjansson, R. Garrett, and W. Zillig, "The genetic element pSSVx of the extremely thermophilic crenarchaeon *Sulfolobus* is a hybrid between a plasmid and a virus," *Molecular Microbiology*, vol. 34, no. 2, pp. 217-226, 1999.

[114] H. P. Arnold, K. M. Stedman, and W. Zillig, "Archaeal phages," in *Encyclopedia of Virology*, R. G. Webster and A. Granoff, Eds. pp. 76-89, Academic Press, London, 1999.

[115] A. M. Baldo and M. A. McClure, "Evolution and horizontal transfer of dUTPase-encoding genes in viruses and their hosts," *Journal of Virology*, vol. 73, no. 9, pp. 7710-7721, 1999.

[116] G. Bertani, "Transduction-like gene transfer in the methanogen *Methanococcus voltae*," *Journal of Bacteriology*, vol. 181, no. 10, pp. 2992-3002, 1999.

[117] J. Conrad, L. Niu, K. Rudd, B. G. Lane, and J. Ofengand, "16S ribosomal RNA pseudouridine synthase RsuA of *Escherichia coli*: deletion, mutation of the conserved Asp102 residue, and sequence comparison among all other pseudouridine synthases," *RNA*, vol. 5, no. 6, pp. 751-763, 1999.

[118] F. Eiserling, A. Pushkin, M. Gingery, and G. Bertani, "Bacteriophage-like particles associated with the gene transfer agent of *Methanococcus voltae* PS," *Journal of General Virology*, vol. 80, pp. 3305-3308, 1999.

[119] P. Forterre, "Displacement of cellular proteins by functional analogues from plasmids or viruses could explain puzzling phylogenies of many DNA informational proteins," *Molecular Microbiology*, vol. 33, no. 3, pp. 457-465, 1999.

[120] R. W. Hendrix, "Evolution: the long evolutionary reach of viruses," *Current Biology*, vol. 9, no. Dec 16-30 (24), p. R914-R917, 1999.

[121] A. Hochheimer, R. Hedderich, and R. K. Thauer, "The DNA binding protein Tfx from *Methanobacterium thermoautotrophicum*: structure, DNA binding properties and transcriptional regulation," *Molecular Microbiology*, vol. 31, no. 2, pp. 641-650, 1999.

[122] K. F. Jarrell, D. P. Bayley, J. D. Correia, and N. A. Thomas, "Recent excitement about the Archaea," *BioScience*, vol. 49, no. 7, pp. 530-541, 1999.

[123] A. V. Klieve and R. S. Hegarty, "Opportunities for biological control of ruminal methanogenesis," *Australian Journal of Agriculture Research*, vol. 50, pp. 1315-1319, 1999.

[124] J. A. Leigh, "Transcriptional regulation in Archaea," *Current Opinion in Microbiology*, vol. 2, no. 2, pp. 131-134, 1999.

[125] K. S. Makarova, L. Aravind, and E. V. Koonin, "A superfamily of archaeal, bacterial, and eukaryotic proteins homologous to animal transglutaminases," *Protein Science*, vol. 8, no. 8, pp. 1714-1719, 1999.

[126] S. I. Makino, N. Amano, H. Koike, and M. Suzuki, "Prophages inserted in archaebacterial genomes," *Proceedings of the Japan Academy Series B Physical and Biological Sciences*, vol. 75, no. 6, pp. 166-171, 1999.

[127] I. Noll, S. Muller, and A. Klein, "Transcriptional regulation of genes encoding the selenium-free [NiFe]-hydrogenases in the archaeon *Methanococcus voltae* involves positive and negative control elements," *Genetics*, vol. 152, no. 4, pp. 1335-1341, 1999.

[128] D. Prangishvili, H. P. Arnold, D. Gotz, U. Ziese, I. Holz, J. K. Kristjansson, and W. Zillig, "A novel virus family, the *Rudiviridae*: Structure, virus-host interactions and genome variability of the sulfolobus viruses SIRV1 and SIRV2," *Genetics*, vol. 152, no. Aug, pp. 1387-1396, 1999.

[129] K. M. Stedman, C. Schleper, E. Rumpf, and W. Zillig, "Genetic requirements for the function of the archaeal virus SSV1 in *Sulfolobus solfataricus*: construction and testing of viral shuttle vectors," *Genetics*, vol. 152, no. 4, pp. 1397-1405, 1999.

[130] D. L. Tumbula and W. B. Whitman, "Genetics of *Methanococcus*: possibilities for functional genomics in Archaea," *Molecular Microbiology*, vol. 33, no. 1, pp. 1-7, 1999.

[131] W. B. Whitman, F. Pfeifer, P. Blum, and A. Klein, "What archaea have to tell biologists," *Genetics*, vol. 152, no. 4, pp. 1245-1248, 1999.

[132] H. P. Arnold, W. Zillig, U. Ziese, I. Holz, M. Crosby, T. Utterback, J. F. Weidmann, J. K. Kristjanson, H. P. Klenk, K. E. Nelson, and C. M. Fraser, "A novel lipothrixvirus, SIFV, of the extremely thermophilic crenarchaeon *Sulfolobus*," *Virology*, vol. 267, no. 2, pp. 252-266, 2000.

[133] H. P. Arnold, U. Ziese, and W. Zillig, "SNDV, a novel virus of the extremely thermophilic and acidophilic archaeon *Sulfolobus*," *Virology*, vol. 272, no. 2, pp. 409-416, 2000.

[134] U. Baranyi, R. Klein, W. Lubitz, D. H. Kruger, and A. Witte, "The archaeal halophilic virus-encoded Dam-like methyltransferase M.φCh1-I methylates adenine residues and complements *dam* mutants in the low salt environment of *Escherichia coli*," *Molecular Microbiology*, vol. 35, no. 5, pp. 1168-1179, 2000.

[135] R. Klein, B. Greineder, U. Baranyi, and A. Witte, "The structural protein E of the archaeal virus *φ*Ch1: evidence for processing in *Natrialba magadii* during virus maturation," *Virology*, vol. 276, no. 2, pp. 376-387, 2000.

[136] E. Martusewitsch, C. W. Sensen, and C. Schleper, "High spontaneous mutation rate in the hyperthermophilic archaeon *Sulfolobus solfataricus* is mediated by transposable elements," *Journal of Bacteriology*, vol. 182, no. 9, pp. 2574-2581, 2000.

[137] X. Peng, I. Holz, W. Zillig, R. A. Garrett, and Q. She, "Evolution of the family of pRN plasmids and their integrase-mediated insertion into the chromosome of the crenarchaeon *Sulfolobus solfataricus*," *Journal of Molecular Biology*, vol. 303, no. 4, pp. 449-454, 2000.

[138] I.-N. Wang, D. L. Smith, and R. Young, "Holins: The protein clocks of bacteriophage infections," *Annual Review of Microbiology*, vol. 54, pp. 799-825, 2000.

[139] A. Wasserfallen, J. Nölling, P. Pfister, J. Reeve, and M. E. Conway de, "Phylogenetic analysis of 18 thermophilic *Methanobacterium* isolates supports the proposals to create a new genus, *Methanothermobacter* gen. nov., and to reclassify several isolates in three species, *Methanothermobacter thermautotrophicus* comb. nov., *Methanothermobacter wolfeii* comb. nov., and *Methanothermobacter marburgensis* sp. nov," *International Journal of Systematic and Evolutionary Microbiology*, vol. 50 Pt 1, pp. 43-53, 2000.

[140] K. E. Wommack and R. R. Colwell, "Virioplankton: viruses in aquatic ecosystems," *Microbiology and Molecular Biology Reviews*, vol. 64, pp. 69-114, 2000.

[141] H.-W. Ackermann, "Frequency of morphological phage descriptions in the year 2000. Brief Review," *Archives of Virology*, vol. 146, pp. 843-857, 2001.

[142] E. C. Holmes, "On the origin and evolution of the human immunodeficiency virus (HIV)," *Biological reviews of the Cambridge Philosophical Society*, vol. 76, no. 2, pp. 239-254, 2001.

[143] R. P. Birkenbihl, K. Neef, D. Prangishvili, and B. Kemper, "Holliday junction resolving enzymes of archaeal viruses SIRV1 and SIRV2," *Journal of Molecular Biology*, vol. 309, no. 5, pp. 1067-1076, 2001.

[144] H. Blum, W. Zillig, S. Mallock, H. Domdey, and D. Prangishvili, "The genome of the archaeal virus SIRV1 has features in common with genomes of eukaryal viruses," *Virology*, vol. 281, no. 1, pp. 6-9, 2001.

[145] K. Brugger, P. Redder, Q. She, F. Confalonieri, Y. Zivanovic, and R. A. Garrett, "Mobile elements in archaeal genomes," *FEMS Microbiology Letters*, vol. 206, no. 2, pp. 131-141, 2002.

[146] R. Cannio, P. Contursi, M. Rossi, and S. Bartolucci, "Thermoadaptation of a mesophilic hygromycin B phosphotransferase by directed evolution in hyperthermophilic Archaea: selection of a stable genetic marker for DNA transfer into *Sulfolobus solfataricus*," *Extremophiles*, vol. 5, no. 3, pp. 153-159, 2001.

[147] R. N. Harty, A. P. Schmitt, F. Bouamr, C. B. Lopez, and C. Krummenacher, "Virus budding/host interactions," *Advances in Virology*, vol. 2011, p. 963192, 2011.

[148] J. S. Hofer and R. Sommaruga, "Seasonal dynamics of viruses in an alpine lake: Importance of filamentous forms," *Aquatic Microbial Ecology*, vol. 26, no. 1, pp. 1-11, 2001.

[149] L. M. Iyer, L. Aravind, and E. V. Koonin, "Common origin of four diverse families of large eukaryotic DNA viruses," *Journal of Virology*, vol. 75, no. 23, pp. 11720-11734, 2001.

[150] M. Kvaratskhelia, B. N. Wardleworth, and M. F. White, "Multiple Holliday junction resolving enzyme activities in the Crenarchaeota and Euryarchaeota," *FEBS Letters*, vol. 491, no. 3, pp. 243-246, 2001.

[151] M. Lange and B. K. Ahring, "A comprehensive study into the molecular methodology and molecular biology of methanogenic Archaea," *FEMS Microbiology Reviews*, vol. 25, no. 5, pp. 553-571, 2001.

[152] Y. Luo and A. Wasserfallen, "Gene transfer systems and their applications in Archaea," *Systematic and Applied Microbiology*, vol. 24, no. 1, pp. 15-25, 2001.

[153] Y. Luo, P. Pfister, T. Leisinger, and A. Wasserfallen, "The genome of archaeal prophage ΨM100 encodes the lytic enzyme responsible for autolysis of *Methanothermobacter wolfeii*," *Journal of Bacteriology*, vol. 183, no. Oct, pp. 5788-5792, 2001.

[154] Y. Luo, T. Leisinger, and A. Wasserfallen, "Comparative sequence analysis of plasmids pME2001 and pME2200 of *Methanothermobacter marburgensis* strains Marburg and ZH3," *Plasmid*, vol. 45, no. 1, pp. 18-30, 2001.

[155] M. A. McClure, "Evolution of the DUT gene: horizontal transfer between host and pathogen in all three domains of life," *Current protein & peptide science*, vol. 2, no. 4, pp. 313-324, 2001.

[156] X. Peng, H. Blum, Q. She, S. Mallok, K. Brügger, R. A. Garrett, and D. Prangishvili, "Sequences and replication of genomes of the archaeal rudiviruses SIRV1 and SIRV2: relationships to the archaeal lipothrixvirus SIFV and some eukaryal viruses," *Virology*, vol. 291, no. 2, pp. 226-234, 2001.

[157] D. Prangishvili, K. Stedman, and W. Zillig, "Viruses of the extremely thermophilic archaeon *Sulfolobus*," *Trends in Microbiology*, vol. 9, no. 1, pp. 39-42, 2001.

[158] G. Rice, K. Stedman, J. Snyder, B. Wiedenheft, D. Willits, S. Brumfield, T. McDermott, and M. J. Young, "Viruses from extreme thermal environments," *Proceedings of the National Academy of Sciences, USA*, vol. 98, no. 23, pp. 13341-13345, 2001.

[159] J. G. Sinkovics, "The place of viruses in the "tree of life"," *Acta Microbiologica et Immunologica Hungarica*, vol. 48, no. 1, pp. 115-127, 2001.

[160] S. J. Williamson, M. R. McLaughlin, and J. H. Paul, "Interaction of the ΦHSIC virus with its host: lysogeny or pseudolysogeny?," *Applied and Environmental Microbiology*, vol. 67, no. 4 (Apr), pp. 1682-1688, 2001.

[161] H.-W. Ackermann, "Bacteriophage observations and evolution," *Research in Microbiology*, vol. 154, no. 4, pp. 245-251, 2003.

[162] M. Bettstetter, X. Peng, R. A. Garrett, and D. Prangishvili, "AFV1, a novel virus infecting hyperthermophilic archaea of the genus *Acidianus*," *Virology*, vol. 315, pp. 68-79, 2003.

[163] M. Ciaramella, F. M. Pisani, and M. Rossi, "Molecular biology of extremophiles: recent progress on the hyperthermophilic *archaeon Sulfolobus*," *Antonie van Leeuwenhoek*, vol. 81, no. 1-4, pp. 85-97, 2002.

[164] C. Desplats and H. M. Krisch, "The diversity and evolution of the T4-type bacteriophages," *Research in Microbiology*, vol. 154, no. 4, pp. 259-267, 2003.

[165] J. Filee, P. Forterre, T. Sen-Lin, and J. Laurent, "Evolution of DNA polymerase families: evidences for multiple gene exchange between cellular and viral proteins," *Journal of Molecular Evolution*, vol. 54, no. 6, pp. 763-773, 2002.

[166] R. W. Hendrix, "Bacteriophages: evolution of the majority," *Theoretical Population Biology*, vol. 61, no. 4, pp. 471-480, 2002.

[167] R. Klein, U. Baranyi, N. Rossler, B. Greineder, H. Scholz, and A. Witte, "*Natrialba magadii* virus fCh1: first complete nucleotide sequence and functional organization of a virus infecting a haloalkaliphilic archaeon," *Molecular Microbiology*, vol. 45, no. 3, pp. 851-863, 2002.

[168] M. Kvaratskhelia, B. N. Wardleworth, C. S. Bond, J. M. Fogg, D. M. Lilley, and M. F. White, "Holliday junction resolution is modulated by archaeal chromatin components *in vitro*," *Journal of Biological Chemistry*, vol. 277, no. 4, pp. 2992-2996, 2002.

[169] A. S. Lang, T. A. Taylor, and J. T. Beatty, "Evolutionary implications of phylogenetic analyses of the gene transfer agent (GTA) of *Rhodobacter capsulatus*," *Journal of Molecular Evolution*, vol. 55, no. 5, pp. 534-543, 2002.

[170] J. G. Lawrence, G. F. Hatfull, and R. W. Hendrix, "Imbroglios of viral taxonomy: genetic exchange and failings of phenetic approaches," *Journal of Bacteriology*, vol. 184, no. 17, pp. 4891-4905, 2002.

[171] S. Lucas, L. Toffin, Y. Zivanovic, D. Charlier, H. Moussard, P. Forterre, D. Prieur, and G. Erauso, "Construction of a shuttle vector for, and spheroplast transformation of, the hyperthermophilic archaeon *Pyrococcus abyssi*," *Applied and Environmental Microbiology*, vol. 68, no. 11, pp. 5528-5536, 2002.

[172] Y. Luo, P. Pfister, T. Leisinger, and A. Wasserfallen, "Pseudomurein endoisopeptidases PeiW and PeiP, two moderately related members of a novel family of proteases produced in *Methanothermobacter* strains," *FEMS Microbiology Letters*, vol. 208, no. 1, pp. 47-51, 2002.

[173] M. S. Mitchell, S. Matsuzaki, S. Imai, and V. B. Rao, "Sequence analysis of bacteriophage T4 DNA packaging/terminase genes *16* and *17* reveals a common ATPase center in the large subunit of viral terminases," *Nucleic Acids Research*, vol. 30, no. 18, pp. 4009-4021, 2002.

[174] K. Neef, R. P. Birkenbihl, and B. Kemper, "Holliday junction-resolving enzymes from eight hyperthermophilic archaea differ in reactions with cruciform DNA," *Extremophiles*, vol. 6, no. 5, pp. 359-367, 2002.

[175] M. M. Poranen, R. Daugelavicius, and D. H. Bamford, "Common principles in viral entry," *Annual Review of Microbiology*, vol. 56, pp. 521-538, 2002.

[176] R. Rachel, M. Bettstetter, B. P. Hedlund, M. Häring, A. Kessler, K. O. Stetter, and D. Prangishvili, "Remarkable morphological diversity of viruses and virus-like particles in hot terrestrial environments," *Archives of Virology*, vol. 147, no. 12, pp. 2419-2429, 2002.

[177] C. Schiraldi, M. Giuliano, and R. M. De, "Perspectives on biotechnological applications of archaea," *Archaea*, vol. 1, no. 2, pp. 75-86, 2002.

[178] M. C. Serre, C. Letzelter, J. R. Garel, and M. Duguet, "Cleavage properties of an archaeal site-specific recombinase, the SSV1 integrase," *Journal of Biological Chemistry*, vol. 277, no. 19, pp. 16758-16767, 2002.

[179] Q. She, K. Brugger, and L. Chen, "Archaeal integrative genetic elements and their impact on genome evolution," *Research in Microbiology*, vol. 153, no. 6, pp. 325-332, 2002.

[180] S. L. Tang, S. Nuttall, K. Ngui, C. Fisher, P. Lopez, and M. Dyall-Smith, "HF2: a double-stranded DNA tailed haloarchaeal virus with a mosaic genome," *Molecular Microbiology*, vol. 44, no. 1, pp. 283-296, 2002.

[181] D. H. Bamford, "Do viruses form lineages across different domains of life?," *Research in Microbiology*, vol. 154, no. 4, pp. 231-236, 2003.

[182] S. Bartolucci, M. Rossi, and R. Cannio, "Characterization and functional complementation of a nonlethal deletion in the chromosome of a β-glycosidase mutant of *Sulfolobus solfataricus*," *Journal of Bacteriology*, vol. 185, no. 13, pp. 3948-3957, 2003.

[183] S. Casjens, "Prophages and bacterial genomics: what have we learned so far?," *Molecular Microbiology*, vol. 49, no. 2, pp. 277-300, 2003.

[184] G. N. Cohen, V. Barbe, D. Flament, M. Galperin, R. Heilig, O. Lecompte, O. Poch, D. Prieur, J. Querellou, R. Ripp, J. C. Thierry, J. van der Oost, J. Weissenbach, Y. Zivanovic, and P. Forterre, "An integrated analysis of the genome of the hyperthermophilic archaeon *Pyrococcus abyssi*," *Molecular Microbiology*, vol. 47, no. 6, pp. 1495-1512, 2003.

[185] P. Contursi, R. Cannio, S. Prato, G. Fiorentino, M. Rossi, and S. Bartolucci, "Development of a genetic system for hyperthermophilic Archaea: expression of a moderate thermophilic bacterial alcohol dehydrogenase gene in *Sulfolobus solfataricus*," *FEMS Microbiology Letters*, vol. 218, no. 1, pp. 115-120, 2003.

[186] M. Dyall-Smith, S.-L. Tang, and C. Bath, "Haloarchaeal viruses: how diverse are they?," *Research in Microbiology*, vol. 154, no. 4, pp. 309-313, 2003.

[187] D. M. Faguy, "Lateral gene transfer (LGT) between Archaea and *Escherichia coli* is a contributor to the emergence of novel infectious disease," *BMC Infectious Diseases*, vol. 3, p. 13, 2003.

[188] J. Filée, P. Forterre, and J. Laurent, "The role played by viruses in the evolution of their hosts: a view based on informational protein phylogenies," *Research in Microbiology*, vol. 154, no. 4, pp. 237-243, 2003.

[189] C. Geslin, M. Le Romancer, M. Gaillard, G. Erauso, and D. Prieur, "Observation of virus-like particles in high temperature enrichment cultures from deep-sea hydrothermal vents," *Research in Microbiology*, vol. 154, no. 4, pp. 303-307, 2003.

[190] C. Geslin, R. M. Le, G. Erauso, M. Gaillard, G. Perrot, and D. Prieur, "PAV1, the first virus-like particle isolated from a hyperthermophilic euryarchaeote, "*Pyrococcus abyssi*"," *Journal of Bacteriology*, vol. 185, no. 13, pp. 3888-3894, 2003.

[191] R. W. Hendrix, "Bacteriophage genomics," *Current Opinion in Microbiology*, vol. 6, no. 5, pp. 506-511, 2003.

[192] M. Jonuscheit, E. Martusewitsch, K. M. Stedman, and C. Schleper, "A reporter gene system for the hyperthermophilic archaeon *Sulfolobus solfataricus* based on a selectable and integrative shuttle vector," *Molecular Microbiology*, vol. 48, no. 5, pp. 1241-1252, 2003.

[193] H. Li, H. Xu, D. E. Graham, and R. H. White, "The *Methanococcus jannaschii* dCTP deaminase is a bifunctional deaminase and diphosphatase," *Journal of Biological Chemistry*, vol. 278, no. 13, pp. 11100-11106, 2003.

[194] D. Prangishvili, "Evolutionary insights from studies on viruses from hot habitats," *Research in Microbiology*, vol. 154, no. **4**, pp. 289-294, 2003.

[195] R. J. Roberts, M. Belfort, T. Bestor, A. S. Bhagwat, T. A. Bickle, J. Bitinaite, R. M. Blumenthal, S. K. Degtyarev, D. T. Dryden, K. Dybvig, K. Firman, E. S. Gromova, R. I. Gumport, S. E. Halford, S. Hattman, J. Heitman, D. P. Hornby, A. Janulaitis, A. Jeltsch, J. Josephsen, A. Kiss, T. R. Klaenhammer, I. Kobayashi, H. Kong, D. H. Kruger, S. Lacks, M. G. Marinus, M. Miyahara, R. D. Morgan, N. E. Murray, V. Nagaraja, A. Piekarowicz, A. Pingoud, E. Raleigh, D. N. Rao, N. Reich, V. E. Repin, E. U. Selker, P. C. Shaw, D. C. Stein, B. L. Stoddard, W. Szybalski, T. A. Trautner, J. L. van Etten, J. M. Vitor, G. G. Wilson, and S. Y. Xu, "A nomenclature for restriction enzymes, DNA methyltransferases, homing endonucleases and their genes," *Nucleic Acids Research*, vol. 31, no. 7, pp. 1805-1812, 2003.

[196] M. Rossi, M. Ciaramella, R. Cannio, F. M. Pisani, M. Moracci, and S. Bartolucci, "Extremophiles 2002," *Journal of Bacteriology*, vol. 185, no. 13, pp. 3683-3689, 2003.

[197] T. Sato, T. Fukui, H. Atomi, and T. Imanaka, "Targeted gene disruption by homologous recombination in the hyperthermophilic archaeon *Thermococcus kodakaraensis* KOD1," *Journal of Bacteriology*, vol. 185, no. 1, pp. 210-220, 2003.

[198] M. C. Serre and M. Duguet, "Enzymes that cleave and religate DNA at high temperature: the same story with different actors," *Progress in Nucleic Acid Research and Molecular Biology*, vol. 74, pp. 37-81, 2003.

[199] J. C. Snyder, K. Stedman, G. Rice, B. Wiedenheft, J. Spuhler, and M. J. Young, "Viruses of hyperthermophilic Archaea," *Research in Microbiology*, vol. 154, no. 7, pp. 474-482, 2003.

[200] K. M. Stedman, Q. She, H. Phan, H. P. Arnold, I. Hoz, R. A. Garrett, and W. Zillig, "Relationships between fuselloviruses infecting the extremely thermophilic archaeon *Sulfolobus*: SSV1 and SSV2," *Research in Microbiology*, vol. 154, no. 4, pp. 295-302, 2003.

[201] S. D. Benson, J. K. Bamford, D. H. Bamford, and R. M. Burnett, "Does common architecture reveal a viral lineage spanning all three domains of life?," *Molecular Cell*, vol. 16, no. 5, pp. 673-685, 2004.

[202] D. Boadi, C. Benchaar, J. Chiquette, and Massé, "Mitigation strategies to reduce enteric methane emissions from dairy cows: update review," *Canadian Journal of Animal Science*, vol. 84, no. 3, pp. 319-335, 2004.

[203] R. Bonneau, N. S. Baliga, E. W. Deutsch, P. Shannon, and L. Hood, "Comprehensive *de novo* structure prediction in a systems-biology context for the archaea *Halobacterium* sp. *NRC-1*," *Genome Biology*, vol. 5, no. 8, p. R52, 2004.

[204] M. Breitbart, L. Wegley, S. Leeds, T. Schoenfeld, and F. Rohwer, "Phage community dynamics in hot springs," *Applied and Environmental Microbiology*, vol. 70, no. 3, pp. 1633-1640, 2004.

[205] H. Cheng, N. Shen, J. Pei, and N. V. Grishin, "Double-stranded DNA bacteriophage prohead protease is homologous to herpesvirus protease," *Protein Science*, vol. 13, no. 8, pp. 2260-2269, 2004.

[206] H. X. Chiura, "Novel broad-host range gene transfer particles in nature," *Microbes and Environments*, vol. 19, no. 4, pp. 249-264, 2004.

[207] M. Häring, X. Peng, K. Brugger, R. Rachel, K. O. Stetter, R. A. Garrett, and D. Prangishvili, "Morphology and genome organization of the virus PSV of the hyperthermophilic archaeal genera *Pyrobaculum* and *Thermoproteus*: a novel virus family, the *Globuloviridae*," *Virology*, vol. 323, no. 2, pp. 233-242, 2004.

[208] R. W. Hendrix, "Hot new virus, deep connections," *Proceedings of the National Academy of Sciences, USA*, vol. 101, no. 20, pp. 7495-7496, 2004.

[209] L. M. Iyer, K. S. Makarova, E. V. Koonin, and L. Aravind, "Comparative genomics of the FtsK-HerA superfamily of pumping ATPases: implications for the origins of chromosome segregation, cell division and viral capsid packaging," *Nucleic Acids Research*, vol. 32, no. 17, pp. 5260-5279, 2004.

[210] S. Jones, "Founding member," *Nature Reviews Microbiology*, vol. 2, pp. 524-525, 2004.

[211] A. Kessler, A. B. Brinkman, J. van der Oost, and D. Prangishvili, "Transcription of the rod-shaped viruses SIRV1 and SIRV2 of the hyperthermophilic archaeon *Sulfolobus*," *Journal of Bacteriology*, vol. 186, no. 22, pp. 7745-7753, 2004.

[212] P. Kraft, A. Oeckinghaus, D. Kummel, G. H. Gauss, J. Gilmore, B. Wiedenheft, M. Young, and C. M. Lawrence, "Crystal structure of F-93 from *Sulfolobus* spindle-shaped virus 1, a winged-helix DNA binding protein," *Journal of Virology*, vol. 78, no. 21, pp. 11544-11550, 2004.

[213] P. Kraft, D. Kummel, A. Oeckinghaus, G. H. Gauss, B. Wiedenheft, M. Young, and C. M. Lawrence, "Structure of D-63 from sulfolobus spindle-shaped virus 1: surface properties of the dimeric four-helix bundle suggest an adaptor protein function," *Journal of Virology*, vol. 78, no. 14, pp. 7438-7442, 2004.

[214] G. Lipps, "The replication protein of the *Sulfolobus islandicus* plasmid pRN1," *Biochemical Society Transactions*, vol. 32, no. 2, pp. 240-244, 2004.

[215] M. Liu, M. Gingery, S. R. Doulatov, Y. Liu, A. Hodes, S. Baker, P. Davis, M. Simmonds, C. Churcher, K. Mungall, M. A. Quail, A. Preston, E. T. Harvill, D. J. Maskell, F. A. Eiserling, J. Parkhill, and J. F. Miller, "Genomic and genetic analysis of *Bordetella* bacteriophages encoding reverse transcriptase-mediated tropism-switching cassettes," *Journal of Bacteriology*, vol. 186, no. 5, pp. 1503-1517, 2004.

[216] M. S. Mitchell and V. B. Rao, "Novel and deviant Walker A ATP-binding motifs in bacteriophage large terminase-DNA packaging proteins," *Virology*, vol. 321, no. 2, pp. 217-221, 2004.

[217] X. Peng, A. Kessler, H. Phan, R. A. Garrett, and D. Prangishvili, "Multiple variants of the archaeal DNA rudivirus SIRV1 in a single host and a novel mechanism of genomic variation," *Molecular Microbiology*, vol. 54, no. 2, pp. 366-375, 2004.

[218] D. Prangishvili and R. A. Garrett, "Exceptionally diverse morphotypes and genomes of crenarchaeal hyperthermophilic viruses," *Biochemical Society Transactions*, vol. 32, no. 2, pp. 204-208, 2004.

[219] D. Prieur, G. Erauso, C. Geslin, S. Lucas, M. Gaillard, A. Bidault, A. C. Mattenet, K. Rouault, D. Flament, P. Forterre, and R. M. Le, "Genetic elements of Thermococcales," *Biochemical Society Transactions*, vol. 32, no. Pt 2, pp. 184-187, 2004.

[220] G. Rice, L. Tang, K. Stedman, F. Roberto, J. Spuhler, E. Gillitzer, J. E. Johnson, T. Douglas, and M. Young, "The structure of a thermophilic archaeal virus shows a double-stranded DNA viral capsid type that spans all domains of life," *Proceedings of the National Academy of Sciences, USA*, vol. 101, no. 20, pp. 7716-7720, 2004.

[221] N. Rossler, R. Klein, H. Scholz, and A. Witte, "Inversion within the haloalkaliphilic virus φCh1 DNA results in differential expression of structural proteins," *Molecular Microbiology*, vol. 52, no. 2, pp. 413-426, 2004.

[222] J. C. Snyder, J. Spuhler, B. Wiedenheft, F. F. Roberto, T. Douglas, and M. J. Young, "Effects of culturing on the population structure of a hyperthermophilic virus," *Microbial Ecology*, vol. 48, pp. 561-566, 2004.

[223] S. L. Tang, S. Nuttall, and M. Dyall-Smith, "Haloviruses HF1 and HF2: evidence for a recent and large recombination event," *Journal of Bacteriology*, vol. 186, no. 9, pp. 2810-2817, 2004.

[224] J. C. Venter, K. Remington, J. F. Heidelberg, A. L. Halpern, D. Rusch, J. A. Eisen, D. Wu, I. Paulsen, K. E. Nelson, W. Nelsen, D. E. Fouts, S. Levy, A. H. Knap, M. W. Lomas, K. Nealson, O. White, J. Peterson, J. Hoffman, R. Parsons, H. Baden-Tillson, C. Pfannkoch, Y.-H. Rogers, and H. O. Smith, "Environmental genome shotgun sequencing of the Sargasso Sea," *Science*, vol. 304, no. 5667, pp. 66-74, 2004.

[225] M. Ventura, C. Canchaya, R. D. Pridmore, and H. Brüssow, "The prophages of *Lactobacillus johnsonii* NCC 533: comparative genomics and transcription analysis," *Virology*, vol. 320, no. 2, pp. 229-242, 2004.

[226] M. G. Weinbauer, "Ecology of prokaryotic viruses," *FEMS Microbiology Reviews*, vol. 28, no. 2, pp. 127-181, 2004.

[227] B. Wiedenheft, K. Stedman, F. Roberto, D. Willits, A. K. Gleske, L. Zoeller, J. Snyder, T. Douglas, and M. Young, "Comparative genomic analysis of hyperthermophilic archaeal *Fuselloviridae* viruses," *Journal of Virology*, vol. 78, no. 4, pp. 1954-1961, 2004.

[228] C. Winter, A. Smit, G. J. Herndl, and M. G. Weinbauer, "Impact of virioplankton on archaeal and bacterial community richness as assessed in seawater batch cultures," *Applied and Environmental Microbiology*, vol. 70, no. 2, pp. 804-813, 2004.

[229] T. Allers and M. Mevarech, "Archaeal genetics - the third way," *Nature Reviews Genetics*, vol. 6, no. 1, pp. 58-73, 2005.

[230] D. H. Bamford, J. J. Ravantti, G. Ronnholm, S. Laurinavicius, P. Kukkaro, M. Dyall-Smith, P. Somerharju, N. Kalkkinen, and J. K. Bamford, "Constituents of SH1, a novel lipid-containing virus infecting the halophilic euryarchaeon *Haloarcula hispanica*," *Journal of Virology*, vol. 79, no. 14, pp. 9097-9107, 2005.

[231] M. Breitbart and F. Rohwer, "Here a virus, there a virus, everywhere the same virus?," *Trends in Microbiology*, vol. 13, no. 6, pp. 278-284, 2005.

[232] K. Carlson, "Working with bacteriophages: common techniques and methodological approaches," in *Bacteriophages: Biology and Application*, E. Kutter and A. Sulakvelidze, Eds. pp. 437-494, CRC Press, Boca Raton, Florida, 2005.

[233] U. Desselberger, "Report on an ICTV-sponsored symposium on Virus Evolution," *Archives of Virology*, vol. 150, no. 3, pp. 629-635, 2005.

[234] E. Ennifar, J. Basquin, R. Birkenbihl, and D. Suck, "Purification, crystallization and preliminary X-ray diffraction studies of the archaeal virus resolvase SIRV2," *Acta Crystallographica Section F, Structural Biology and Crystallization Communications*, vol. 61, no. 5, pp. 507-509, 2005.

[235] L. A. Fernández, "Exploring prokaryotic diversity: there are other molecular worlds," *Molecular Microbiology*, vol. 55, no. 1, pp. 5-15, 2005.

[236] T. Fukui, H. Atomi, T. Kanai, R. Matsumi, S. Fujiwara, and T. Imanaka, "Complete genome sequence of the hyperthermophilic archaeon *Thermococcus kodakaraensis* KOD1 and comparison with *Pyrococcus* genomes," *Genome Research*, vol. 15, no. 3, pp. 352-363, 2005.

[237] M. Haring, G. Vestergaard, R. Rachel, L. Chen, R. A. Garrett, and D. Prangishvili, "Virology: independent virus development outside a host," *Nature*, vol. 436, no. 7054, pp. 1101-1102, 2005.

[238] M. Häring, G. Vestergaard, K. Brugger, R. Rachel, R. A. Garrett, and D. Prangishvili, "Structure and genome organization of AFV2, a novel archaeal lipothrixvirus with unusual terminal and core structures," *Journal of Bacteriology*, vol. 187, no. 11, pp. 3855-3858, 2005.

[239] M. Häring, R. Rachel, X. Peng, R. A. Garrett, and D. Prangishvili, "Viral diversity in hot springs of Pozzuoli, Italy, and characterization of a unique archaeal virus, *Acidianus* bottle-shaped virus, from a new family, the *Ampullaviridae*," *Journal of Virology*, vol. 79, no. 15, pp. 9904-9911, 2005.

[240] R. Khayat, L. Tang, E. T. Larson, C. M. Lawrence, M. Young, and J. E. Johnson, "Structure of an archaeal virus capsid protein reveals a common ancestry to eukaryotic and bacterial viruses," *Proceedings of the National Academy of Sciences, USA*, vol. 102, no. 52, pp. 18944-18949, 2005.

[241] H. Koike, K. Yokoyama, T. Kawashima, T. Yamasaki, S. Makino, L. Clowney, and M. Suzuki, "GATC methylation by Dam methylase in archae: its roles and possible transcription regulation by FFRP," *Proceedings of the Japanese Academy Series B*, vol. 81, pp. 278-290, 2012.

[242] P. A. Laurinmaki, J. T. Huiskonen, D. H. Bamford, and S. J. Butcher, "Membrane proteins modulate the bilayer curvature in the bacterial virus Bam35," *Structure*, vol. 13, no. 12, pp. 1819-1828, 2005.

[243] X. Liu and F. Yang, "Identification and function of a shrimp white spot syndrome virus (WSSV) gene that encodes a dUTPase," *Virus Research*, vol. 110, no. 1-2, pp. 21-30, 2005.

[244] J. B. H. Martiny and D. Field, "Ecological perspectives on the sequenced genome collection," *Ecology Letters*, vol. 8, no. 12, pp. 1334-1345, 2005.

[245] F. J. Mojica, C. Diez-Villasenor, J. Garcia-Martinez, and E. Soria, "Intervening sequences of regularly spaced prokaryotic repeats derive from foreign genetic elements," *Journal of Molecular Evolution*, vol. 60, no. 2, pp. 174-182, 2005.

[246] K. Namba, K. Hagiwara, H. Tanaka, Y. Nakaishi, K. T. Chong, E. Yamashita, G. E. Armah, Y. Ono, Y. Ishino, T. Omura, T. Tsukihara, and A. Nakagawa, "Expression and molecular characterization of spherical particles derived from the genome of the hyperthermophilic euryarchaeote *Pyrococcus furiosus*," *Journal of Biochemistry*, vol. 138, no. 2, pp. 193-199, 2005.

[247] J. L. Parker and M. F. White, "The endonuclease Hje catalyses rapid, multiple turnover resolution of Holliday junctions," *Journal of Molecular Biology*, vol. 350, no. 1, pp. 1-6, 2005.

[248] S. Paukner, P. Kudela, G. Kohl, T. Schlapp, S. Friedrichs, and W. Lubitz, "DNA-loaded bacterial ghosts efficiently mediate reporter gene transfer and expression in macrophages," *Molecular Therapy*, vol. 11, no. 2, pp. 215-223, 2005.

[249] K. Porter, P. Kukkaro, J. K. Bamford, C. Bath, H. M. Kivela, M. L. Dyall-Smith, and D. H. Bamford, "SH1: A novel, spherical halovirus isolated from an Australian hypersaline lake," *Virology*, vol. 335, no. 1, pp. 22-33, 2005.

[250] D. Prangishvili and R. A. Garrett, "Viruses of hyperthermophilic Crenarchaea," *Trends in Microbiology*, vol. 13, no. 11, pp. 535-542, 2005.

[251] L. Randau, M. Pearson, and D. Soll, "The complete set of tRNA species in *Nanoarchaeum equitans*," *FEBS Letters*, vol. 579, no. 13, pp. 2945-2947, 2005.

[252] A. M. Saren, J. J. Ravantti, S. D. Benson, R. M. Burnett, L. Paulin, D. H. Bamford, and J. K. H. Bamford, "A snapshot of viral evolution from genome analysis of the tectiviridae family," *Journal of Molecular Biology*, vol. 350, no. 3, pp. 427-440, 2005.

[253] N. J. Stromsten, D. H. Bamford, and J. K. Bamford, "In vitro DNA packaging of PRD1: a common mechanism for internal-membrane viruses," *Journal of Molecular Biology*, vol. 348, no. 3, pp. 617-629, 2005.

[254] K. Suhre, S. Audic, and J. M. Claverie, "Mimivirus gene promoters exhibit an unprecedented conservation among all eukaryotes," *Proceedings of the National Academy of Sciences, USA*, vol. 102, no. 41, pp. 14689-14693, 2005.

[255] M. T. Teixeira and E. Gilson, "Telomere maintenance, function and evolution: the yeast paradigm," *Chromosome Research*, vol. 13, no. 5, pp. 535-548, 2005.

[256] N. Umadevi, S. Kumar, and N. Narayana, "Crystallization and preliminary X-ray diffraction studies of the WW4 domain of the Nedd4-2 ubiquitin-protein ligase," *Acta Crystallographica Section F, Structural Biology and Crystallization Communications*, vol. 61, no. 12, pp. 1084-1086, 2005.

[257] G. Vestergaard, M. Häring, X. Peng, R. Rachel, R. A. Garrett, and D. Prangishvili, "A novel rudivirus, ARV1, of the hyperthermophilic archaeal genus *Acidianus*," *Virology*, vol. 336, no. 1, pp. 83-92, 2005.

[258] L. P. Villarreal, *Viruses and the Evolution of Life*, ASM Press, Washington, D.C., 2005.

[259] X. Xiang, L. Chen, X. Huang, Y. Luo, Q. She, and L. Huang, "*Sulfolobus tengchongensis* spindle-shaped virus STSV1: virus-host interactions and genomic features," *Journal of Virology*, vol. 79, no. 14, pp. 8677-8686, 2005.

[260] M. Young, B. Wiedenheft, J. Snyder, J. Spuhler, F. Roberto, and T. Douglas, "Archaeal viruses from Yellowstone's high temperature environments," in *Geothermal Biology and Geochemistry in Yellowstone Nationtal Park*, pp. 289-304, 2005.

[261] D. G. Ahn, S. I. Kim, J. K. Rhee, K. P. Kim, J. G. Pan, and J. W. Oh, "TTSV1, a new virus-like particle isolated from the hyperthermophilic crenarchaeote *Thermoproteus tenax*," *Virology*, vol. 351, no. 2, pp. 280-290, 2006.

[262] S. V. Albers, M. Jonuscheit, S. Dinkelaker, T. Urich, A. Kletzin, R. Tampe, A. J. Driessen, and C. Schleper, "Production of recombinant and tagged proteins in the hyperthermophilic archaeon *Sulfolobus solfataricus*," *Applied and Environmental Microbiology*, vol. 72, no. 1, pp. 102-111, 2006.

[263] T. Aucelli, P. Contursi, M. Girfoglio, M. Rossi, and R. Cannio, "A spreadable, non-integrative and high copy number shuttle vector for *Sulfolobus solfataricus* based on the genetic element pSSVx from *Sulfolobus islandicus*," *Nucleic Acids Research*, vol. 34, no. 17, p. e114, 2006.

[264] C. Bath, T. Cukalac, K. Porter, and M. L. Dyall-Smith, "His1 and His2 are distantly related, spindle-shaped haloviruses belonging to the novel virus group, *Salterprovirus*," *Virology*, vol. 350, no. 1, pp. 228-239, 2006.

[265] M. Bose and R. D. Barber, "Prophage Finder: a prophage loci prediction tool for prokaryotic genome sequences," *In silico biology*, vol. 6, no. 3, pp. 223-227, 2006.

[266] R. M. Burnett, "More barrels from the viral tree of life," *Proceedings of the National Academy of Sciences, USA*, vol. 103, no. 1, pp. 3-4, 2006.

[267] P. Contursi, S. Jensen, T. Aucelli, M. Rossi, S. Bartolucci, and Q. She, "Characterization of the *Sulfolobus* host-SSV2 virus interaction," *Extremophiles*, vol. 10, no. 6, pp. 615-627, 2006.

[268] R. Dorazi, J. L. Parker, and M. F. White, "PCNA activates the Holliday junction endonuclease Hjc," *Journal of Molecular Biology*, vol. 364, no. 3, pp. 243-247, 2006.

[269] P. Durand, F. Mahe, A. S. Valin, and J. Nicolas, "Browsing repeats in genomes: Pygram and an application to non-coding region analysis," *BMC Bioinformatics*, vol. 7, p. 477, 2006.

[270] G. Erauso, K. M. Stedman, H. J. van de Werken, W. Zillig, and J. van der Oost, "Two novel conjugative plasmids from a single strain of *Sulfolobus*," *Microbiology (Reading)*, vol. 152, no. Pt 7, pp. 1951-1968, 2006.

[271] P. Forterre, "The origin of viruses and their possible roles in major evolutionary transitions," *Virus Research*, vol. 117, no. 1, pp. 5-16, 2006.

[272] P. Forterre, "DNA topoisomerase V: a new fold of mysterious origin," *Trends in Biotechnology*, vol. 24, no. 6, pp. 245-247, 2006.

[273] A. Goulet, S. Spinelli, V. Campanacci, S. Porciero, S. Blangy, R. A. Garrett, T. H. van, N. Leulliot, T. Basta, D. Prangishvili, and C. Cambillau, "Crystallization and preliminary X-ray diffraction analysis of protein 14 from *Sulfolobus islandicus* filamentous virus (SIFV)," *Acta Crystallographica Section F, Structural Biology and Crystallization Communications*, vol. 62, no. Pt 9, pp. 884-886, 2006.

[274] A. Kessler, G. Sezonov, J. I. Guijarro, N. Desnoues, T. Rose, M. Delepierre, S. D. Bell, and D. Prangishvili, "A novel archaeal regulatory protein, Sta1, activates transcription from viral promoters," *Nucleic Acids Research*, vol. 34, no. 17, pp. 4837-4845, 2006.

[275] H. M. Kivela, E. Roine, P. Kukkaro, S. Laurinavicius, P. Somerharju, and D. H. Bamford, "Quantitative dissociation of archaeal virus SH1 reveals distinct capsid proteins and a lipid core," *Virology*, vol. 356, no. 1-2, pp. 4-11, 2006.

[276] E. V. Koonin, "Temporal order of evolution of DNA replication systems inferred by comparison of cellular and viral DNA polymerases," *Biology Direct*, vol. 1, p. 39, 2006.

[277] E. V. Koonin and V. V. Dolja, "Evolution of complexity in the viral world: The dawn of a new vision," *Virus Research*, vol. 117, no. 1, pp. 1-4, 2006.

[278] E. V. Koonin, T. G. Senkevich, and V. V. Dolja, "The ancient Virus World and evolution of cells," *Biology Direct*, vol. 1, p. 29, 2006.

[279] E. T. Larson, D. Reiter, M. Young, and C. M. Lawrence, "Structure of A197 from *Sulfolobus* turreted icosahedral virus: a crenarchaeal viral glycosyltransferase exhibiting the GT-A fold," *Journal of Virology*, vol. 80, no. 15, pp. 7636-7644, 2006.

[280] B. A. Legault, A. Lopez-Lopez, J. C. Alba-Casado, W. F. Doolittle, H. Bolhuis, F. Rodriguez-Valera, and R. T. Papke, "Environmental genomics of "*Haloquadratum walsbyi*" in a saltern crystallizer indicates a large pool of accessory genes in an otherwise coherent species," *BMC Genomics*, vol. 7, p. 171, 2006.

[281] R. Leplae, G. Lima-Mendez, and A. Toussaint, "A first global analysis of plasmid encoded proteins in the ACLAME database," *FEMS Microbiology Reviews*, vol. 30, no. 6, pp. 980-994, 2006.

[282] R. K. Lillestol, P. Redder, R. A. Garrett, and K. Brugger, "A putative viral defence mechanism in archaeal cells," *Archaea (Vancouver, B C )*, vol. 2, no. 1, pp. 59-72, 2006.

[283] G. Lipps, "Plasmids and viruses of the thermoacidophilic crenarchaeote *Sulfolobus*," *Extremophiles*, vol. 10, no. 1, pp. 17-28, 2006.

[284] W. S. Maaty, A. C. Ortmann, M. Dlakic, K. Schulstad, J. K. Hilmer, L. Liepold, B. Weidenheft, R. Khayat, T. Douglas, M. J. Young, and B. Bothner, "Characterization of the archaeal thermophile *Sulfolobus* turreted icosahedral virus validates an evolutionary link among double-stranded DNA viruses from all domains of life," *Journal of Virology*, vol. 80, no. 15, pp. 7625-7635, 2006.

[285] M. A. Mayo and L. A. Ball, "ICTV in San Francisco: a report from the Plenary Session," *Archives of Virology*, vol. 151, no. 2, pp. 413-422, 2006.

[286] I. J. Molineux, "Fifty-three years since Hershey and Chase; much ado about pressure but which pressure is it?," *Virology*, vol. 344, no. 1, pp. 221-229, 2006.

[287] K. Nakamura, T. Terada, Y. Sekiguchi, N. Shinzato, X. Y. Meng, M. Enoki, and Y. Kamagata, "Application of pseudomurein endoisopeptidase to fluorescence in situ hybridization of methanogens within the family *Methanobacteriaceae*," *Applied and Environmental Microbiology*, vol. 72, no. 11, pp. 6907-6913, 2006.

[288] T. Naryshkina, J. Liu, L. Florens, S. K. Swanson, A. R. Pavlov, N. V. Pavlova, R. Inman, L. Minakhin, S. A. Kozyavkin, M. Washburn, A. Mushegian, and K. Severinov, "*Thermus thermophilus* bacteriophage φYS40 genome and proteomic characterization of virions," *Journal of Molecular Biology*, vol. 364, no. 4, pp. 667-677, 2006.

[289] H. Onimatsu, K. Suganuma, S. Uenoyama, and T. Yamada, "C-terminal repetitive motifs in Vp130 present at the unique vertex of the *Chlorovirus* capsid are essential for binding to the host *Chlorella* cell wall," *Virology*, vol. 353, no. 2, pp. 433-442, 2006.

[290] A. C. Ortmann, B. Wiedenheft, T. Douglas, and M. Young, "Hot crenarchaeal viruses reveal deep evolutionary connections," *Nature Reviews Microbiology*, vol. 4, no. 7, pp. 520-528, 2006.

[291] D. Prangishvili, P. Forterre, and R. A. Garrett, "Viruses of the Archaea: a unifying view," *Nature Reviews Microbiology*, vol. 4, no. 11, pp. 837-848, 2006.

[292] D. Prangishvili, G. Vestergaard, M. Häring, R. Aramayo, T. Basta, R. Rachel, and R. A. Garrett, "Structural and genomic properties of the hyperthermophilic archaeal virus ATV with an extracellular stage of the reproductive cycle," *Journal of Molecular Biology*, vol. 359, no. 5, pp. 1203-1216, 2006.

[293] D. Prangishvili, R. A. Garrett, and E. V. Koonin, "Evolutionary genomics of archaeal viruses: unique viral genomes in the third domain of life," *Virus Research*, vol. 117, no. 1, pp. 52-67, 2006.

[294] U. Rass and S. C. West, "Synthetic junctions as tools to identify and characterize Holliday junction resolvases," *Methods in Enzymology*, vol. 408, pp. 485-501, 2006.

[295] D. Ratel, J. L. Ravanat, F. Berger, and D. Wion, "N6-methyladenine: the other methylated base of DNA," *Bioessays*, vol. 28, no. 3, pp. 309-315, 2006.

[296] M. Skurnik and E. Strauch, "Phage therapy: facts and fiction," *International Journal of Medical Microbiology*, vol. 296, no. 1, pp. 5-14, 2006.

[297] K. M. Stedman, A. Clore, and Y. Combet-Blanc, "Biogeographical diversity of archaeal viruses," in *Prokaryotic Diversity: Mechanisms and Significance*, N. A. Logan, H. M. Lappin-Scott, and P. C. F. Oyston, Eds. pp. 131-143, Cambridge University Press, Cambridge, UK, 2006.

[298] K. M. Stedman, D. Prangishvili, and W. Zillig, "Viruses of Archaea," in *The Bacteriophages*, R. Calendar and S. T. Abedon, Eds. pp. 499-516, Oxford University Press, Oxford, 2006.

[299] P. J. Steenbakkers, W. J. Geerts, N. A. Ayman-Oz, and J. T. Keltjens, "Identification of pseudomurein cell wall binding domains," *Molecular Microbiology*, vol. 62, no. 6, pp. 1618-1630, 2006.

[300] G. Witzany, "Natural genome-editing competences of viruses," *Acta Biotheoretica*, vol. 54, no. 4, pp. 235-253, 2006.

[301] P. Worning, L. J. Jensen, P. F. Hallin, H. H. Staerfeldt, and D. W. Ussery, "Origin of replication in circular prokaryotic chromosomes," *Environmental Microbiology*, vol. 8, no. 2, pp. 353-361, 2006.

[302] H.-W. Ackermann, "5500 phages examined in the electron microscope," *Archives of Virology*, vol. 152, pp. 227-243, 2007.

[303] H. W. Ackermann and A. M. Kropinski, "Curated list of prokaryote viruses with fully sequenced genomes," *Research in Microbiology*, vol. 158, no. 7, pp. 555-566, 2007.

[304] F. Akita, K. T. Chong, H. Tanaka, E. Yamashita, N. Miyazaki, Y. Nakaishi, M. Suzuki, K. Namba, Y. Ono, T. Tsukihara, and A. Nakagawa, "The crystal structure of a virus-like particle from the hyperthermophilic archaeon *Pyrococcus furiosus* provides insight into the evolution of viruses," *Journal of Molecular Biology*, vol. 368, no. 5, pp. 1469-1483, 2007.

[305] E. E. Allen, G. W. Tyson, R. J. Whitaker, J. C. Detter, P. M. Richardson, and J. F. Banfield, "Genome dynamics in a natural archaeal population," *Proceedings of the National Academy of Sciences, USA*, vol. 104, no. 6, pp. 1883-1888, 2007.

[306] D. V. Alzhanova, A. I. Prokhnevsky, V. V. Peremyslov, and V. V. Dolja, "Virion tails of *Beet yellows virus*: Coordinated assembly by three structural proteins," *Virology*, vol. 359, no. 1, pp. 220-226, 2007.

[307] R. Barrangou, C. Fremaux, H. Deveau, M. Richards, P. Boyaval, S. Moineau, D. A. Romero, and P. Horvath, "CRISPR provides acquired resistance against viruses in prokaryotes," *Science*, vol. 315, no. 5819, pp. 1709-1712, 2007.

[308] K. Brügger, L. Chen, M. Stark, A. Zibat, P. Redder, A. Ruepp, M. Awayez, Q. She, R. A. Garrett, and H. P. Klenk, "The genome of *Hyperthermus butylicus*: a sulfur-reducing, peptide fermenting, neutrophilic Crenarchaeote growing up to 108 degrees C," *Archaea*, vol. 2, no. 2, pp. 127-135, 2007.

[309] A. J. Clore and K. M. Stedman, "The SSV1 viral integrase is not essential," *Virology*, vol. 361, no. 1, pp. 103-111, 2007.

[310] P. Contursi, R. Cannio, S. Prato, Q. She, M. Rossi, and S. Bartolucci, "Transcriptional analysis of the genetic element pSSVx: differential and temporal regulation of gene expression reveals correlation between transcription and replication," *Journal of Bacteriology*, vol. 189, no. 17, pp. 6339-6350, 2007.

[311] S. Cuadros-Orellana, A.-B. Martin-Cuadrado, B. Legault, G. D'Auria, O. Zhaxybayeva, R. T. Papke, and F. Rodriguez-Valera, "Genomic plasticity in prokaryotes: the case of the square haloarchaeon," *ISME Journal*, vol. 1, no. 3, pp. 235-245, 2007.

[312] J. Filée, P. Siguier, and M. Chandler, "Insertion sequence diversity in archaea," *Microbiology and Molecular Biology Reviews*, vol. 71, no. 1, pp. 121-157, 2007.

[313] S. Fröls, P. M. Gordon, M. A. Panlilio, I. G. Duggin, S. D. Bell, C. W. Sensen, and C. Schleper, "Response of the hyperthermophilic archaeon *Sulfolobus solfataricus* to UV damage," *Journal of Bacteriology*, vol. 189, no. 23, pp. 8708-8718, 2007.

[314] S. Fröls, P. M. Gordon, M. A. Panlilio, C. Schleper, and C. W. Sensen, "Elucidating the transcription cycle of the UV-inducible hyperthermophilic archaeal virus SSV1 by DNA microarrays," *Virology*, vol. 365, no. 1, pp. 48-59, 2007.

[315] C. Geslin, M. Gaillard, D. Flament, K. Rouault, R. M. Le, D. Prieur, and G. Erauso, "Analysis of the first genome of a hyperthermophilic marine virus-like particle, PAV1, isolated from *Pyrococcus abyssi*," *Journal of Bacteriology*, vol. 189, no. 12, pp. 4510-4519, 2007.

[316] J. T. Huiskonen and S. J. Butcher, "Membrane-containing viruses with icosahedrally symmetric capsids," *Current Opinion in Structural Biology*, vol. 17, no. 2, pp. 229-236, 2007.

[317] M. Iro, R. Klein, B. Galos, U. Baranyi, N. Rossler, and A. Witte, "The lysogenic region of virus *φ*Ch1: identification of a repressor-operator system and determination of its activity in halophilic *Archaea*," *Extremophiles*, vol. 11, no. 2, pp. 383-396, 2007.

[318] J. Keller, N. Leulliot, C. Cambillau, V. Campanacci, S. Porciero, D. Prangishvili, P. Forterre, D. Cortez, S. Quevillon-Cheruel, and T. H. van, "Crystal structure of AFV3-109, a highly conserved protein from crenarchaeal viruses," *Virology Journal*, vol. 4, p. 12, 2007.

[319] N. P. King, E. O. Yeates, and T. O. Yeates, "Identification of rare slipknots in proteins and their implications for stability and folding," *Journal of Molecular Biology*, vol. 373, no. 1, pp. 153-166, 2007.

[320] E. T. Larson, B. Eilers, S. Menon, D. Reiter, A. Ortmann, M. J. Young, and C. M. Lawrence, "A winged-helix protein from Sulfolobus turreted icosahedral virus points toward stabilizing disulfide bonds in the intracellular proteins of a hyperthermophilic virus," *Virology*, vol. 368, no. 2, pp. 249-261, 2007.

[321] E. T. Larson, B. J. Eilers, D. Reiter, A. C. Ortmann, M. J. Young, and C. M. Lawrence, "A new DNA binding protein highly conserved in diverse crenarchaeal viruses," *Virology*, vol. 363, no. 2, pp. 387-396, 2007.

[322] G. Lima-Mendez, A. Toussaint, and R. Leplae, "Analysis of the phage sequence space: the benefit of structured information," *Virology*, vol. 365, no. 2, pp. 241-249, 2007.

[323] Y. Mei, J. Chen, D. Sun, D. Chen, Y. Yang, P. Shen, and X. Chen, "Induction and preliminary characterization of a novel halophage SNJ1 from lysogenic *Natrinema* sp. F5," *Canadian Journal of Microbiology*, vol. 53, no. 9, pp. 1106-1110, 2007.

[324] M. Merabishvili, R. Verhelst, T. Glonti, N. Chanishvili, V. Krylov, C. Cuvelier, M. Tediashvili, and M. Vaneechoutte, "Digitized fluorescent RFLP analysis (fRFLP) as a universal method for comparing genomes of culturable dsDNA viruses: application to bacteriophages," *Research in Microbiology*, vol. 158, no. 7, pp. 572-581, 2007.

[325] E. Pagaling, R. D. Haigh, W. D. Grant, D. A. Cowan, B. E. Jones, Y. Ma, A. Ventosa, and S. Heaphy, "Sequence analysis of an archaeal virus isolated from a hypersaline lake in Inner Mongolia, China," *BMC Genomics*, vol. 8, p. 410, 2007.

[326] M. O. Park, H. Ikenaga, and K. Watanabe, "Phage diversity in a methanogenic digester," *Microbial Ecology*, vol. 53, no. 1, pp. 98-103, 2007.

[327] X. Peng, T. Basta, M. Häring, R. A. Garrett, and D. Prangishvili, "Genome of the *Acidianus* bottle-shaped virus and insights into the replication and packaging mechanisms," *Virology*, vol. 364, no. 1, pp. 237-243, 2007.

[328] A. M. Poole and E. Willerslev, "Can identification of a fourth domain of life be made from sequence data alone, and could it be done on Mars?," *Astrobiology*, vol. 7, no. 5, pp. 801-814, 2007.

[329] K. Porter, B. R. Russ, and M. L. Dyall-Smith, "Virus–host interactions in salt lakes," *Current Opinion in Microbiology*, vol. 10, no. 4, pp. 418-424, 2007.

[330] D. Prangishvili, "Editorial: the 90th anniversary of "bacteriophage"," *Research in Microbiology*, vol. 158, no. 7, pp. 551-552, 2007.

[331] S. A. Qureshi, "Protein-DNA interactions at the *Sulfolobus* spindle-shaped virus-1 (SSV1) T5 and T6 gene promoters," *Canadian Journal of Microbiology*, vol. 53, no. 9, pp. 1076-1083, 2007.

[332] J. M. Russell, J. W. Barnett, E. Désilets, and S. Bertrand, "Mitigation strateties to reduce GHG emmissions from the dairy industry," *Bulletin of the International Dairy Federation*, vol. 422, pp. 30-44, 2007.

[333] B. S. Samuel, E. E. Hansen, J. K. Manchester, P. M. Coutinho, B. Henrissat, R. Fulton, P. Latreille, K. Kim, R. K. Wilson, and J. I. Gordon, "Genomic and metabolic adaptations of *Methanobrevibacter smithii* to the human gut," *Proceedings of the National Academy of Sciences, USA*, vol. 104, no. 25, pp. 10643-10648, 2007.

[334] F. Santos, A. Meyerdierks, A. Pena, R. Rossello-Mora, R. Amann, and J. Anton, "Metagenomic approach to the study of halophages: the environmental halophage 1," *Environmental Microbiology*, vol. 9, no. 7, pp. 1711-1723, 2007.

[335] P. Serwer, "Evolution and the complexity of bacteriophages," *Virology Journal*, vol. 4, p. 30, 2007.

[336] P. Serwer, S. J. Hayes, J. A. Thomas, G. A. Griess, and S. C. Hardies, "Rapid determination of genomic DNA length for new bacteriophages," *Electrophoresis*, vol. 28, no. 12, pp. 1896-1902, 2007.

[337] A. Shinkai, S. Sekine, A. Urushibata, T. Terada, M. Shirouzu, and S. Yokoyama, "The putative DNA-binding protein Sto12a from the thermoacidophilic archaeon *Sulfolobus tokodaii* contains intrachain and interchain disulfide bonds," *Journal of Molecular Biology*, vol. 372, no. 5, pp. 1293-1304, 2007.

[338] J. C. Snyder, B. Wiedenheft, M. Lavin, F. F. Roberto, J. Spuhler, A. C. Ortmann, T. Douglas, and M. Young, "Virus movement maintains local virus population diversity," *Proceedings of the Royal Society of London Series B*, vol. 104, no. 48, pp. 19102-19107, 2007.

[339] T. B. Stanton, "Prophage-like gene transfer agents-novel mechanisms of gene exchange for *Methanococcus*, *Desulfovibrio*, *Brachyspira*, and *Rhodobacter* species," *Anaerobe*, vol. 13, no. 2, pp. 43-49, 2007.

[340] A. Toussaint, G. Lima-Mendez, and R. Leplae, "PhiGO, a phage ontology associated with the ACLAME database," *Research in Microbiology*, vol. 158, no. 7, pp. 567-571, 2007.

[341] Y. Wang, Z. Duan, H. Zhu, X. Guo, Z. Wang, J. Zhou, Q. She, and L. Huang, "A novel *Sulfolobus* non-conjugative extrachromosomal genetic element capable of integration into the host genome and spreading in the presence of a fusellovirus," *Virology*, vol. 363, no. 1, pp. 124-133, 2007.

[342] K. B. Zeldovich, P. Chen, and E. I. Shakhnovich, "Protein stability imposes limits on organism complexity and speed of molecular evolution," *Proceedings of the National Academy of Sciences, USA*, vol. 104, no. 41, pp. 16152-16157, 2007.

[343] Z. Zhao, F. Ke, J. Gui, and Q. Zhang, "Characterization of an early gene encoding for dUTPase in *Rana grylio* virus," *Virus Research*, vol. 123, no. 2, pp. 128-137, 2007.

[344] N. G. Abrescia, J. M. Grimes, H. M. Kivela, R. Assenberg, G. C. Sutton, S. J. Butcher, J. K. Bamford, D. H. Bamford, and D. I. Stuart, "Insights into virus evolution and membrane biogenesis from the structure of the marine lipid-containing bacteriophage PM2," *Molecular Cell*, vol. 31, no. 5, pp. 749-761, 2008.

[345] S. V. Albers and A. J. Driessen, "Conditions for gene disruption by homologous recombination of exogenous DNA into the *Sulfolobus solfataricus* genome," *Archaea*, vol. 2, no. 3, pp. 145-149, 2008.

[346] I. Anderson, J. Rodriguez, D. Susanti, I. Porat, C. Reich, L. E. Ulrich, J. G. Elkins, K. Mavromatis, A. Lykidis, E. Kim, L. S. Thompson, M. Nolan, M. Land, A. Copeland, A. Lapidus, S. Lucas, C. Detter, I. B. Zhulin, G. J. Olsen, W. Whitman, B. Mukhopadhyay, J. Bristow, and N. Kyrpides, "Genome sequence of *Thermofilum pendens* reveals an exceptional loss of biosynthetic pathways without genome reduction," *Journal of Bacteriology*, vol. 190, no. 8, pp. 2957-2965, 2008.

[347] S. Andersson, "Description of virus capsid structures with methods from inorganic solid state chemistry," *Zeitschrift für Anorganische und Allgemeine Chemie*, vol. 634, pp. 2504-2510, 2008.

[348] S. Andersson, "The structure of virus capsids," *Zeitschrift für Anorganische und Allgemeine Chemie*, vol. 634, pp. 2161-2170, 2008.

[349] A. F. Andersson and J. F. Banfield, "Virus population dynamics and acquired virus resistance in natural microbial communities," *Science*, vol. 320, no. 5879, pp. 1047-1050, 2008.

[350] C. Ash, J. Foley, and E. Pennisi, "Microbial ecology. Lost in microbial space. Special section introduction," *Science*, vol. 320, no. 5879, p. 1027, 2008.

[351] S. Berkner and G. Lipps, "Genetic tools for *Sulfolobus* spp.: vectors and first applications," *Archives of Microbiology*, vol. 190, no. 3, pp. 217-230, 2008.

[352] A. Bize, X. Peng, M. Prokofeva, K. Maclellan, S. Lucas, P. Forterre, R. A. Garrett, E. A. Bonch-Osmolovskaya, and D. Prangishvili, "Viruses in acidic geothermal environments of the Kamchatka Peninsula," *Research in Microbiology*, vol. 159, no. 5, pp. 358-366, 2008.

[353] G. Caetano-Anolles, F. J. Sun, M. Wang, L. S. Yafremava, A. Harish, H. S. Kim, V. Knudsen, D. Caetano-Anolles, and J. E. Mittenthal, "Origins and evolution of modern biochemistry: insights from genomes and molecular structure," *Frontiers in Bioscience*, vol. 13, pp. 5212-5240, 2008.

[354] A. M. Comeau, G. F. Hatfull, H. M. Krisch, D. Lindell, N. H. Mann, and D. Prangishvili, "Exploring the prokaryotic virosphere," *Research in Microbiology*, vol. 159, no. 5, pp. 306-313, 2008.

[355] R. Danovaro, C. Corinaldesi, M. Filippini, U. R. Fischer, M. O. Gessner, S. Jacquet, M. Magagnini, and B. Velimirov, "Viriobenthos in freshwater and marine sediments: a review," *Freshwater Biology*, vol. 53, no. 6, pp. 1186-1213, 2008.

[356] J. Filee, N. Pouget, and M. Chandler, "Phylogenetic evidence for extensive lateral acquisition of cellular genes by Nucleocytoplasmic large DNA viruses," *BMC Evolutionary Biology*, vol. 8, p. 320, 2008.

[357] M. C. Gutierrez, A. M. Castillo, E. Pagaling, S. Heaphy, M. Kamekura, Y. Xue, Y. Ma, D. A. Cowan, B. E. Jones, W. D. Grant, and A. Ventosa, "*Halorubrum kocurii* sp. nov., an archaeon isolated from a saline lake," *International Journal of Systematic and Evolutionary Microbiology*, vol. 58, no. Pt 9, pp. 2031-2035, 2008.

[358] G. F. Hatfull, "Bacteriophage genomics," *Current Opinion in Microbiology*, vol. 11, no. 5, pp. 447-453, 2008.

[359] H. T. Jaalinoja, E. Roine, P. Laurinmaki, H. M. Kivela, D. H. Bamford, and S. J. Butcher, "Structure and host-cell interaction of SH1, a membrane-containing, halophilic euryarchaeal virus," *Proceedings of the National Academy of Sciences, USA*, vol. 105, no. 23, pp. 8008-8013, 2008.

[360] S. T. Jaatinen, L. J. Happonen, P. Laurinmaki, S. J. Butcher, and D. H. Bamford, "Biochemical and structural characterisation of membrane-containing icosahedral dsDNA bacteriophages infecting thermophilic *Thermus thermophilus*," *Virology*, vol. 379, no. 1, pp. 10-19, 2008.

[361] E. F. Jackson and C. R. Jackson, "Viruses in wetland ecosystems," *Freshwater Biology*, vol. 53, no. 6, pp. 1214-1227, 2008.

[362] S. Kato, T. Kosaka, and K. Watanabe, "Comparative transcriptome analysis of responses of *Methanothermobacter thermautotrophicus* to different environmental stimuli," *Environmental Microbiology*, vol. 10, no. 4, pp. 893-905, 2008.

[363] H. M. Kivelä, S. Madonna, M. Krupovic, M. L. Tutino, and J. K. Bamford, "Genetics for *Pseudoalteromonas* provides tools to manipulate marine bacterial virus PM2," *Journal of Bacteriology*, vol. 190, no. 4, pp. 1298-1307, 2008.

[364] E. V. Koonin, Y. I. Wolf, K. Nagasaki, and V. V. Dolja, "The Big Bang of picorna-like virus evolution antedates the radiation of eukaryotic supergroups," *Nature Reviews Microbiology*, vol. 6, no. 12, pp. 925-939, 2008.

[365] J. S. Koti, M. C. Morais, R. Rajagopal, B. A. Owen, C. T. McMurray, and D. L. Anderson, "DNA packaging motor assembly intermediate of bacteriophage φ29," *Journal of Molecular Biology*, vol. 381, no. 5, pp. 1114-1132, 2008.

[366] H. M. Krisch and A. M. Comeau, "The immense journey of bacteriophage T4-rom d'Hérelle to Delbrück and then to Darwin and beyond," *Research in Microbiology*, vol. 159, no. 5, pp. 314-324, 2008.

[367] M. Krupovic and D. H. Bamford, "Archaeal proviruses TKV4 and MVV extend the PRD1-adenovirus lineage to the phylum *Euryarchaeota*," *Virology*, vol. 375, no. 1, pp. 292-300, 2008.

[368] M. Krupovic and D. H. Bamford, "Virus evolution: how far does the double β-barrel viral lineage extend?," *Nature Reviews Microbiology*, vol. 6, no. 12, pp. 941-948, 2008.

[369] P. Kudela, S. Paukner, U. B. Mayr, D. Cholujova, G. Kohl, Z. Schwarczova, J. Bizik, J. Sedlak, and W. Lubitz, "Effective gene transfer to melanoma cells using bacterial ghosts," *Cancer Letters*, vol. 262, no. 1, pp. 54-63, 2008.

[370] J. E. Kyle, H. S. Eydal, F. G. Ferris, and K. Pedersen, "Viruses in granitic groundwater from 69 to 450 m depth of the Asp+¦ hard rock laboratory, Sweden," *ISME Journal*, vol. 2, no. 5, pp. 571-574, 2008.

[371] J. E. Kyle, K. Pedersen, and F. G. Ferris, "Virus Mineralization at Low pH in the Rio Tinto, Spain," *Geomicrobiology Journal*, vol. 25, no. 7-8, pp. 338-345, 2008.

[372] B. La Scola, C. Desnues, I. Pagnier, C. Robert, L. Barrassi, G. Fournous, M. Merchat, M. Suzan-Monti, P. Forterre, E. Koonin, and D. Raoult, "The virophage as a unique parasite of the giant mimivirus," *Nature*, vol. 455, no. 7209, pp. 100-104, 2008.

[373] A. C. Lindas, E. A. Karlsson, M. T. Lindgren, T. J. Ettema, and R. Bernander, "A unique cell division machinery in the Archaea," *Proceedings of the National Academy of Sciences, USA*, vol. 105, no. 48, pp. 18942-18946, 2008.

[374] B. Liu and X. Zhang, "Deep-sea thermophilic *Geobacillus* bacteriophage GVE2 transcriptional profile and proteomic characterization of virions," *Applied Microbiology and Biotechnology*, vol. 80, no. 4, pp. 697-707, 2008.

[375] L. A. Marraffini and E. J. Sontheimer, "CRISPR interference limits horizontal gene transfer in staphylococci by targeting DNA," *Science*, vol. 322, no. 5909, pp. 1843-1845, 2008.

[376] C. Martin, D. P. Morgavi, and M. Doreau, "Methane mitigation in ruminants: from microbe to the farm scale," *Animal*, vol. 4, no. 3, pp. 351-365, 2010.

[377] A. T. McGeoch and S. D. Bell, "Extra-chromosomal elements and the evolution of cellular DNA replication machineries," *Nature Reviews Molecular Cell Biology*, vol. 9, no. 7, pp. 569-574, 2008.

[378] S. K. Menon, W. S. Maaty, G. J. Corn, S. C. Kwok, B. J. Eilers, P. Kraft, E. Gillitzer, M. J. Young, B. Bothner, and C. M. Lawrence, "Cysteine usage in *Sulfolobus* spindle-shaped virus 1 and extension to hyperthermophilic viruses in general," *Virology*, vol. 376, no. 2, pp. 270-278, 2008.

[379] D. Moreira and C. +. Brochier-Armanet, "Giant viruses, giant chimeras: the multiple evolutionary histories of Mimivirus genes," *BMC Evolutionary Biology*, vol. 8, p. 12, 2008.

[380] A. C. Ortmann, S. K. Brumfield, J. Walther, K. McInnerney, S. J. Brouns, H. J. van de Werken, B. Bothner, T. Douglas, J. van de Oost, and M. J. Young, "Transcriptome analysis of infection of the archaeon *Sulfolobus solfataricus* with *Sulfolobus* turreted icosahedral virus," *Journal of Virology*, vol. 82, no. 10, pp. 4874-4883, 2008.

[381] X. Peng, "Evidence for the horizontal transfer of an integrase gene from a fusellovirus to a pRN-like plasmid within a single strain of *Sulfolobus* and the implications for plasmid survival," *Microbiology (Reading)*, vol. 154, no. Pt 2, pp. 383-391, 2008.

[382] K. Porter and M. L. Dyall-Smith, "Transfection of haloarchaea by the DNAs of spindle and round haloviruses and the use of transposon mutagenesis to identify non-essential regions," *Molecular Microbiology*, vol. 70, no. 5, pp. 1236-1245, 2008.

[383] K. Porter, B. E. Russ, J. Yang, and M. L. Dyall-Smith, "The transcription programme of the protein-primed halovirus SH1," *Microbiology (Reading)*, vol. 154, no. 11, pp. 3599-3608, 2008.

[384] E. Prestel, S. Salamitou, and M. S. DuBow, "An examination of the bacteriophages and bacteria of the Namib desert," *Journal of microbiology (Seoul, Korea)*, vol. 46, no. 4, pp. 364-372, 2008.

[385] D. T. Pride and T. Schoenfeld, "Genome signature analysis of thermal virus metagenomes reveals Archaea and thermophilic signatures," *BMC Genomics*, vol. 9, p. 420, 2008.

[386] L. Randau and D. Soll, "Transfer RNA genes in pieces," *EMBO Rep*, vol. 9, no. 7, pp. 623-628, 2008.

[387] D. Raoult and P. Forterre, "Redefining viruses: lessons from Mimivirus," *Nature Reviews Microbiology*, vol. 6, no. 4, pp. 315-319, 2008.

[388] C. Säwström, J. Lisle, A. M. Anesio, J. C. Priscu, and J. Laybourn-Parry, "Bacteriophage in polar inland waters," *Extremophiles*, vol. 12, no. 2, pp. 167-175, 2008.

[389] T. Schoenfeld, M. Patterson, P. M. Richardson, K. E. Wommack, M. Young, and D. Mead, "Assembly of viral metagenomes from yellowstone hot springs," *Applied and Environmental Microbiology*, vol. 74, no. 13, pp. 4164-4174, 2008.

[390] J. G. Sinkovics and J. C. Horvath, "Natural and genetically engineered viral agents for oncolysis and gene therapy of human cancers," *Archivum Immunologiae et Therapiae Experimentalis*, vol. 56, no. S1, pp. 3s-59s, 2008.

[391] R. Smallridge, "A virus gets a virus," *Nature Reviews Microbiology*, vol. 6, no. 10, p. 714, 2008.

[392] N. Soler, E. Marguet, J. M. Verbavatz, and P. Forterre, "Virus-like vesicles and extracellular DNA produced by hyperthermophilic archaea of the order Thermococcales," *Research in Microbiology*, vol. 159, no. 5, pp. 390-399, 2008.

[393] N. F. Steinmetz, A. Bize, R. C. Findlay, G. P. Lomonossoff, M. Manchester, D. J. Evans, and D. Prangishvili, "Site-specific and spatially controlled addressability of a new viral nanobuilding block: *Sulfolobus islandicus* rod-shaped virus 2," *Advanced Functional Materials*, vol. 18, pp. 3478-3486, 2008.

[394] F. J. Sun and G. Caetano-Anolles, "Evolutionary patterns in the sequence and structure of transfer RNA: early origins of Archaea and viruses," *PLoS Computational Biology*, vol. 4, no. 3, p. e1000018, 2008.

[395] F. J. Sun and G. Caetano-Anolles, "Transfer RNA and the origins of diversified life," *Science Progress*, vol. 91, no. Pt 3, pp. 265-284, 2008.

[396] R. L. V. Thurber, K. L. Barott, D. Hall, H. Liu, B. Rodriguez-Mueller, C. Desnues, R. A. Edwards, M. Haynes, F. E. Angly, L. Wegley, and F. L. Rohwer, "Metagenomic analysis indicates that stressors induce production of herpes-like viruses in the coral *Porites compressa*," *Proceedings of the National Academy of Sciences, USA*, vol. 105, no. 47, pp. 18413-18418, 2008.

[397] G. Vestergaard, S. A. Shah, A. Bize, W. Reitberger, M. Reuter, H. Phan, A. Briegel, R. Rachel, R. A. Garrett, and D. Prangishvili, "*Stygiolobus* rod-shaped virus and the interplay of crenarchaeal rudiviruses with the CRISPR antiviral system," *Journal of Bacteriology*, vol. 190, no. 20, pp. 6837-6845, 2008.

[398] G. Vestergaard, R. Aramayo, T. Basta, M. Haring, X. Peng, K. Brugger, L. Chen, R. Rachel, N. Boisset, R. A. Garrett, and D. Prangishvili, "Structure of the *Acidianus* filamentous virus 3 and comparative genomics of related archaeal lipothrixviruses," *Journal of Virology*, vol. 82, no. 1, pp. 371-381, 2008.

[399] C. Winter, M. M. Moeseneder, G. J. Herndl, and M. G. Weinbauer, "Relationship of geographic distance, depth, temperature, and viruses with prokaryotic communities in the eastern tropical Atlantic Ocean," *Microbial Ecology*, vol. 56, no. 2, pp. 383-389, 2008.

[400] G. Witzany, "Bio-communication of bacteria and their evolutionary roots in natural genome editing competences of viruses," *The Open Evolution Jouranl*, vol. 2, pp. 44-54, 2008.

[401] G. Witzany, "The viral origins of telomeres and telomerases and their important role in eukaryogenesis and genome maintenance," *Biosemiotics*, vol. 1, no. 2, pp. 191-206, 2008.

[402] H. W. Ackermann, "Phage classification and characterization," *Methods in Molecular Biology*, vol. 501, pp. 127-140, 2009.

[403] B. J. Baker, G. W. Tyson, L. Goosherst, and J. F. Banfield, "Insights into the diversity of eukaryotes in acid mine drainage biofilm communities," *Applied and Environmental Microbiology*, vol. 75, no. 7, pp. 2192-2199, 2009.

[404] J. F. Banfield and M. Young, "Microbiology. Variety-the splice of life-in microbial communities," *Science*, vol. 326, no. 5957, pp. 1198-1199, 2009.

[405] T. Basta, J. Smyth, P. Forterre, D. Prangishvili, and X. Peng, "Novel archaeal plasmid pAH1 and its interactions with the lipothrixvirus AFV1," *Molecular Microbiology*, vol. 71, no. 1, pp. 23-34, 2009.

[406] P. J. Bell, "The viral eukaryogenesis hypothesis: a key role for viruses in the emergence of eukaryotes from a prokaryotic world environment," *Annals of the New York Academy of Sciences*, vol. 1178, pp. 91-105, 2009.

[407] A. Bize, E. A. Karlsson, K. Ekefjard, T. E. Quax, M. Pina, M. C. Prevost, P. Forterre, O. Tenaillon, R. Bernander, and D. Prangishvili, "A unique virus release mechanism in the Archaea," *Proceedings of the National Academy of Sciences, USA*, vol. 106, no. 27, pp. 11306-11311, 2009.

[408] M. L. Bochman and A. Schwacha, "The Mcm complex: unwinding the mechanism of a replicative helicase," *Microbiology and Molecular Biology Reviews*, vol. 73, no. 4, pp. 652-683, 2009.

[409] S. K. Brumfield, A. C. Ortmann, V. Ruigrok, P. Suci, T. Douglas, and M. J. Young, "Particle assembly and ultrastructural features associated with replication of the lytic archaeal virus *Sulfolobus* turreted icosahedral virus," *Journal of Virology*, vol. 83, no. 12, pp. 5964-5970, 2009.

[410] H. Brüssow, "The not so universal tree of life or the place of viruses in the living world," *Philosophical Transactions of the Royal Society of London B Biological Sciences*, vol. 364, no. 1527, pp. 2263-2274, 2009.

[411] M. Buée, W. De Boer, and F. Martin, "The rhizosphere zoo: An overview of plant-associated communities of microorganisms, including phages, bacteria, archaea, and fungi, and of some of their structuring factors," *Plant and Soil*, vol. 321, pp. 189-212, 2009.

[412] N. Byrne, F. Lesongeur, N. Bienvenu, C. Geslin, K. Alain, D. Prieur, and A. Godfroy, "Effect of variation of environmental conditions on the microbial communities of deep-sea vent chimneys, cultured in a bioreactor," *Extremophiles*, vol. 13, no. 4, pp. 595-608, 2009.

[413] A. M. Cerdeño-Tárraga, "Genome watch: What a scorcher!," *Nature Reviews Microbiology*, vol. 7, no. 6, pp. 408-409, 2009.

[414] L. R. Comolli, B. J. Baker, K. H. Downing, C. E. Siegerist, and J. F. Banfield, "Three-dimensional analysis of the structure and ecology of a novel, ultra-small archaeon," *ISME Journal*, vol. 3, no. 2, pp. 159-167, 2009.

[415] D. Cortez, P. Forterre, and S. Gribaldo, "A hidden reservoir of integrative elements is the major source of recently acquired foreign genes and ORFans in archaeal and bacterial genomes," *Genome Biology*, vol. 10, no. 6, p. R65, 2009.

[416] S. DasSarma, J. A. Coker, and P. DasSama, "Archaea (overview)," in *The Desktop Encyclopedia of Microbiology*, M. Schaecter, Ed., pp. 118-139, Elsevier, Oxford, 2012.

[417] G. J. Dick, A. F. Andersson, B. J. Baker, S. L. Simmons, B. C. Thomas, A. P. Yelton, and J. F. Banfield, "Community-wide analysis of microbial genome sequence signatures," *Genome Biology*, vol. 10, no. 8, p. R85, 2009.

[418] C. Díez-Villaseñor, C. Almendros, J. García-Martínez, and F. J. Mojica, "Diversity of CRISPR loci in *Escherichia coli*," *Microbiology (Reading)*, vol. 156, no. 5, pp. 1351-1361, 2010.

[419] T. J. Ettema and R. Bernander, "Cell division and the ESCRT complex: A surprise from the archaea," *Communicative and Integrative Biology*, vol. 2, no. 2, pp. 86-88, 2009.

[420] D. J. Evans, "Exploitation of plant and archaeal viruses in bionanotechnology," *Biochemical Society Transactions*, vol. 37, no. 4, pp. 665-670, 2009.

[421] P. Forterre and D. Prangishvili, "The origin of viruses," *Research in Microbiology*, vol. 160, no. 7, pp. 466-472, 2009.

[422] P. Forterre and D. Prangishvili, "The great billion-year war between ribosome- and capsid-encoding organisms (cells and viruses) as the major source of evolutionary novelties," *Annals of the New York Academy of Sciences*, vol. 1178, pp. 65-77, 2009.

[423] K. Fujishima, J. Sugahara, M. Tomita, and A. Kanai, "Large-scale tRNA intron transposition in the archaeal order Thermoproteales represents a novel mechanism of intron gain," *Molecular Biology and Evolution*, vol. 27, no. 10, pp. 2233-2243, 2010.

[424] J. Fulton, B. Bothner, M. Lawrence, J. E. Johnson, T. Douglas, and M. Young, "Genetics, biochemistry and structure of the archaeal virus STIV," *Biochemical Society Transactions*, vol. 37, no. Pt 1, pp. 114-117, 2009.

[425] A. Goulet, S. Spinelli, S. Blangy, T. H. van, N. Leulliot, T. Basta, D. Prangishvili, C. Cambillau, and V. Campanacci, "The thermo- and acido-stable ORF-99 from the archaeal virus AFV1," *Protein Science*, vol. 18, no. 6, pp. 1316-1320, 2009.

[426] A. Goulet, S. Blangy, P. Redder, D. Prangishvili, C. Felisberto-Rodrigues, P. Forterre, V. Campanacci, and C. Cambillau, "*Acidianus* filamentous virus 1 coat proteins display a helical fold spanning the filamentous archaeal viruses lineage," *Proceedings of the National Academy of Sciences, USA*, vol. 106, no. 50, pp. 21155-21160, 2009.

[427] A. Goulet, S. Spinelli, S. Blangy, T. H. van, N. Leulliot, T. Basta, D. Prangishvili, C. Cambillau, and V. Campanacci, "The crystal structure of ORF14 from *Sulfolobus islandicus* filamentous virus," *Proteins: Structure, Function, and Bioinformatics*, vol. 76, no. 4, pp. 1020-1022, 2009.

[428] F. Guilliere, N. Peixeiro, A. Kessler, B. Raynal, N. Desnoues, J. Keller, M. Delepierre, D. Prangishvili, G. Sezonov, and J. I. Guijarro, "Structure, function, and targets of the transcriptional regulator SvtR from the hyperthermophilic archaeal virus SIRV1," *Journal of Biological Chemistry*, vol. 284, no. 33, pp. 22222-22237, 2009.

[429] N. L. Held and R. J. Whitaker, "Viral biogeography revealed by signatures in *Sulfolobus islandicus* genomes," *Environmental Microbiology*, vol. 11, no. 2, pp. 457-466, 2009.

[430] P. Hyman and S. T. Abedon, "Bacteriophage (overview)," in *The Desktop Encyclopedia of Microbiology*, M. Schaecter, Ed., pp. 166-182, Elsevier, Oxford, 2012.

[431] M. Jalasvuori and J. K. Bamford, "Did the ancient crenarchaeal viruses from the dawn of life survive exceptionally well the eons of meteorite bombardment?," *Astrobiology*, vol. 9, no. 1, pp. 131-137, 2009.

[432] M. Jalasvuori, S. T. Jaatinen, S. Laurinavicius, E. Ahola-Iivarinen, N. Kalkkinen, D. H. Bamford, and J. K. H. Bamford, "The closest relatives of icosahedral viruses of thermophilic bacteria are among viruses and plasmids of the halophilic archaea," *Journal of Virology*, vol. 83, no. 18, pp. 9388-9397, 2009.

[433] M. Jalasvuori, A. Örmäläa, and J. K. H. Bamford, "On the astrobiological relevance of viruses in extraterrestrial ecosystems," *International Journal of Astrobiology*, vol. 8, no. 2, pp. 95-100, 2009.

[434] J. Keller, N. Leulliot, B. Collinet, V. Campanacci, C. Cambillau, D. Prangishvilli, and T. H. van, "Crystal structure of AFV1-102, a protein from the acidianus filamentous virus 1," *Protein Science*, vol. 18, no. 4, pp. 845-849, 2009.

[435] J. Keller, N. Leulliot, N. Soler, B. Collinet, R. Vincentelli, P. Forterre, and T. H. van, "A protein encoded by a new family of mobile elements from Euryarchaea exhibits three domains with novel folds," *Protein Science*, vol. 18, no. 4, pp. 850-855, 2009.

[436] E. V. Koonin, "On the origin of cells and viruses: primordial virus world scenario," *Annals of the New York Academy of Sciences*, vol. 1178, pp. 47-64, 2009.

[437] A. M. Kropinski, D. Prangishvili, and R. Lavigne, "Position paper: the creation of a rational scheme for the nomenclature of viruses of *Bacteria* and *Archaea*," *Environmental Microbiology*, vol. 11, no. 11, pp. 2775-2777, 2009.

[438] P. Kukkaro and D. H. Bamford, "Virus-host interactions in environments with a wide range of ionic strengths," *Environmental Microbiology Reports*, vol. 1, no. 1, pp. 71-77, 2009.

[439] A. S. Lang, M. L. Rise, A. I. Culley, and G. F. Steward, "RNA viruses in the sea," *FEMS Microbiology Reviews*, vol. 33, no. 2, pp. 295-323, 2009.

[440] R. Lavigne, P. Darius, E. J. Summer, D. Seto, P. Mahadevan, A. S. Nilsson, H. W. Ackermann, and A. M. Kropinski, "Classification of *Myoviridae* bacteriophages using protein sequence similarity," *BMC Microbiology*, vol. 9, p. 224, 2009.

[441] C. M. Lawrence, S. Menon, B. J. Eilers, B. Bothner, R. Khayat, T. Douglas, and M. J. Young, "Structural and functional studies of archaeal viruses," *Journal of Biological Chemistry*, vol. 284, no. 19, pp. 12599-12603, 2009.

[442] B. Liu, F. Zhou, S. Wu, Y. Xu, and X. Zhang, "Genomic and proteomic characterization of a thermophilic *Geobacillus* bacteriophage GBSV1," *Research in Microbiology*, vol. 160, no. 2, pp. 166-171, 2009.

[443] D. Moreira and P. Lopez-Garcia, "Ten reasons to exclude viruses from the tree of life," *Nature Reviews Microbiology*, vol. 7, no. 4, pp. 306-311, 2009.

[444] R. T. Papke, "A critique of prokaryotic species concepts," *Methods in Molecular Biology*, vol. 532, no. 22, pp. 379-395, 2009.

[445] M. K. Pietilä, E. Roine, L. Paulin, N. Kalkkinen, and D. H. Bamford, "An ssDNA virus infecting archaea: a new lineage of viruses with a membrane envelope," *Molecular Microbiology*, vol. 72, no. 2, pp. 307-319, 2009.

[446] N. V. Ravin, A. V. Mardanov, A. V. Beletsky, I. V. Kublanov, T. V. Kolganova, A. V. Lebedinsky, N. A. Chernyh, E. A. Bonch-Osmolovskaya, and K. G. Skryabin, "Complete genome sequence of the anaerobic, protein-degrading hyperthermophilic crenarchaeon *Desulfurococcus kamchatkensis*," *Journal of Bacteriology*, vol. 191, no. 7, pp. 2371-2379, 2009.

[447] P. Redder, X. Peng, K. Brugger, S. A. Shah, F. Roesch, B. Greve, Q. She, C. Schleper, P. Forterre, R. A. Garrett, and D. Prangishvili, "Four newly isolated fuselloviruses from extreme geothermal environments reveal unusual morphologies and a possible interviral recombination mechanism," *Environmental Microbiology*, vol. 11, no. 11, pp. 2849-2862, 2009.

[448] F. Rohwer and R. V. Thurber, "Viruses manipulate the marine environment," *Nature*, vol. 459, no. 7244, pp. 207-212, 2009.

[449] F. Rohwer, D. Prangishvili, and D. Lindell, "Roles of viruses in the environment," *Environmental Microbiology*, vol. 11, no. 11, pp. 2771-2774, 2009.

[450] K. Rosario, C. Nilsson, Y. W. Lim, Y. Ruan, and M. Breitbart, "Metagenomic analysis of viruses in reclaimed water," *Environmental Microbiology*, vol. 11, no. 11, pp. 2806-2820, 2009.

[451] B. Roucourt and R. Lavigne, "The role of interactions between phage and bacterial proteins within the infected cell: a diverse and puzzling interactome," *Environmental Microbiology*, vol. 11, no. 11, pp. 2789-2805, 2009.

[452] S. Sabet, L. Diallo, L. Hays, W. Jung, and J. G. Dillon, "Characterization of halophiles isolated from solar salterns in Baja California, Mexico," *Extremophiles*, vol. 13, no. 4, pp. 643-656, 2009.

[453] M. Sanchez, M. Drechsler, H. Stark, and G. Lipps, "DNA translocation activity of the multifunctional replication protein ORF904 from the archaeal plasmid pRN1," *Nucleic Acids Research*, vol. 37, no. 20, pp. 6831-6848, 2009.

[454] C. Schlenker, S. Menon, C. M. Lawrence, and V. Copie, "(1)H, (13)C, (15)N backbone and side chain NMR resonance assignments for E73 from *Sulfolobus* spindle-shaped virus ragged hills, a hyperthermophilic crenarchaeal virus from Yellowstone National Park," *Biomolecular NMR assignments*, vol. 3, no. 2, pp. 219-222, 2009.

[455] Y. Sevastsyanovich, S. Alfasi, and J. Cole, "Recombinant protein production: a comparative view on host physiology," *New Biotechnology*, vol. 25, no. 4, pp. 175-180, 2009.

[456] S. A. Shah, N. R. Hansen, and R. A. Garrett, "Distribution of CRISPR spacer matches in viruses and plasmids of crenarchaeal acidothermophiles and implications for their inhibitory mechanism," *Biochemical Society Transactions*, vol. 37, no. Pt 1, pp. 23-28, 2009.

[457] J. I. Sulkowska, P. Sulkowski, and J. Onuchic, "Dodging the crisis of folding proteins with knots," *Proceedings of the National Academy of Sciences, USA*, vol. 106, no. 9, pp. 3119-3124, 2009.

[458] B. R. Szymczyna, R. E. Taurog, M. J. Young, J. C. Snyder, J. E. Johnson, and J. R. Williamson, "Synergy of NMR, computation, and X-ray crystallography for structural biology," *Structure*, vol. 17, no. 4, pp. 499-507, 2009.

[459] R. V. Thurber, M. Haynes, M. Breitbart, L. Wegley, and F. Rohwer, "Laboratory procedures to generate viral metagenomes," *Nature Protocols*, vol. 4, no. 4, pp. 470-483, 2009.

[460] W. Valdivia-Granda and F. Larson, "ORION-VIRCAT: a tool for mapping ICTV and NCBI taxonomies," *Database (Oxford)*, vol. 2009, p. bap014, 2009.

[461] T. E. Waddell, K. Franklin, A. Mazzocco, A. M. Kropinski, and R. P. Johnson, "Generalized transduction by lytic bacteriophages," *Methods in Molecular Biology*, vol. 501, pp. 293-303, 2009.

[462] P. Wilmes, S. L. Simmons, V. J. Denef, and J. F. Banfield, "The dynamic genetic repertoire of microbial communities," *FEMS Microbiology Reviews*, vol. 33, no. 1, pp. 109-132, 2009.

[463] G. Witzany, "Noncoding RNAs: persistent viral agents as modular tools for cellular needs," *Annals of the New York Academy of Sciences*, vol. 1178, pp. 244-267, 2009.

[464] S. Wu, B. Liu, and X. Zhang, "Identification of a tail assembly gene cluster from deep-sea thermophilic bacteriophage GVE2," *Virus Genes*, vol. 38, no. 3, pp. 507-514, 2009.

[465] C. Xiao, Y. G. Kuznetsov, S. Sun, S. L. Hafenstein, V. A. Kostyuchenko, P. R. Chipman, M. Suzan-Monti, D. Raoult, A. McPherson, and M. G. Rossmann, "Structural studies of the giant mimivirus," *PLoS Biology*, vol. 7, no. 4, p. e92, 2009.

[466] X. Yan, Z. Yu, P. Zhang, A. J. Battisti, H. A. Holdaway, P. R. Chipman, C. Bajaj, M. Bergoin, M. G. Rossmann, and T. S. Baker, "The capsid proteins of a large, icosahedral dsDNA virus," *Journal of Molecular Biology*, vol. 385, no. 4, pp. 1287-1299, 2009.

[467] S. Yokobori, T. Itoh, S. Yoshinari, N. Nomura, Y. Sako, A. Yamagishi, T. Oshima, K. Kita, and Y. Watanabe, "Gain and loss of an intron in a protein-coding gene in Archaea: the case of an archaeal RNA pseudouridine synthase gene," *BMC Evolutionary Biology*, vol. 9, p. 198, 2009.

[468] N. Yutin and E. V. Koonin, "Evolution of DNA ligases of nucleo-cytoplasmic large DNA viruses of eukaryotes: a case of hidden complexity," *Biology Direct*, vol. 4, p. 51, 2009.

[469] Y. Zivanovic, J. Armengaud, A. Lagorce, C. Leplat, P. Guerin, M. Dutertre, V. Anthouard, P. Forterre, P. Wincker, and F. Confalonieri, "Genome analysis and genome-wide proteomics of *Thermococcus gammatolerans*, the most radioresistant organism known amongst the Archaea," *Genome Biology*, vol. 10, no. 6, p. R70, 2009.

[470] V. I. Agol, "Which cam first, the virus or the cell?," *Paleontological Journal*, vol. 44, no. 7, pp. 728-736, 2010.

[471] R. Bernander and T. J. Ettema, "FtsZ-less cell division in archaea and bacteria," *Current Opinion in Microbiology*, vol. 13, no. 6, pp. 747-752, 2010.

[472] Y. Bettarel, A. Desnues, and E. Rochelle-Newall, "Lytic failure in cross-inoculation assays between phages and prokaryotes from three aquatic sites of contrasting salinity," *FEMS Microbiology Letters*, vol. 311, no. 2, pp. 113-118, 2010.

[473] J. M. Claverie and C. Abergel, "Mimivirus: the emerging paradox of quasi-autonomous viruses," *Trends in Genetics*, vol. 26, no. 10, pp. 431-437, 2010.

[474] P. Contursi, R. Cannio, and Q. She, "Transcription termination in the plasmid/virus hybrid pSSVx from *Sulfolobus islandicus*," *Extremophiles*, vol. 14, no. 5, pp. 453-463, 2010.

[475] R. F. de Souza, L. M. Iyer, and L. Aravind, "Diversity and evolution of chromatin proteins encoded by DNA viruses," *Biochimica et Biophysica Acta*, vol. 1799, no. 3-4, pp. 302-318, 2010.

[476] P. Deschavanne, M. S. DuBow, and C. Regeard, "The use of genomic signature distance between bacteriophages and their hosts displays evolutionary relationships and phage growth cycle determination," *Virology Journal*, vol. 7, no. 1, p. 163, 2010.

[477] C. Desnues and D. Raoult, "Inside the lifestyle of the virophage," *Intervirology*, vol. 53, no. 5, pp. 293-303, 2010.

[478] H. Deveau, J. E. Garneau, and S. Moineau, "CRISPR/Cas system and its role in phage-bacteria interactions," *Annual Review of Microbiology*, vol. 64, pp. 475-493, 2010.

[479] M. Deza, "Some problems, I care most," *European Journal of Combinatorics*, vol. 31, pp. 649-675, 2010.

[480] R. M. N. Fard, M. D. Barton, and M. W. Heuzenroeder, "Novel bacteriophage in *Enterococcus* spp.," *Current Microbiology*, vol. 60, pp. 400-406, 2010.

[481] S. Fisher, "Are RNA viruses vestiges of an RNA world?," *Journal for General Philosophy of Science*, vol. 41, no. 1, pp. 67-87, 2010.

[482] R. M. Flugel, "The precellular scenario of genovirions," *Virus Genes*, vol. 40, no. 2, pp. 151-154, 2010.

[483] P. Forterre, "Defining life: the virus viewpoint," *Origins of Life and Evolution of the Biosphere*, vol. 40, no. 2, pp. 151-160, 2010.

[484] P. Forterre, "Giant viruses: conflicts in revisiting the virus concept," *Intervirology*, vol. 53, no. 5, pp. 362-378, 2010.

[485] C. Y. Fu, K. Wang, L. Gan, J. Lanman, R. Khayat, M. J. Young, G. J. Jensen, P. C. Doerschuk, and J. E. Johnson, "In vivo assembly of an archaeal virus studied with whole-cell electron cryotomography," *Structure*, vol. 18, no. 12, pp. 1579-1586, 2010.

[486] R. A. Garrett, D. Prangishvili, S. A. Shah, M. Reuter, K. O. Stetter, and X. Peng, "Metagenomic analyses of novel viruses and plasmids from a cultured environmental sample of hyperthermophilic neutrophiles," *Environmental Microbiology*, vol. 12, no. 11, pp. 2918-2930, 2010.

[487] A. Goulet, M. Pina, P. Redder, D. Prangishvili, L. Vera, J. Lichiere, N. Leulliot, T. H. van, M. Ortiz-Lombardia, V. Campanacci, and C. Cambillau, "ORF157 from the archaeal virus *Acidianus* filamentous virus 1 defines a new class of nuclease," *Journal of Virology*, vol. 84, no. 10, pp. 5025-5031, 2010.

[488] A. Goulet, G. Vestergaard, C. Felisberto-Rodrigues, V. Campanacci, R. A. Garrett, C. Cambillau, and M. Ortiz-Lombardia, "Getting the best out of long-wavelength X-rays: *de novo* chlorine/sulfur SAD phasing of a structural protein from ATV," *Acta Crystallographica Section D Biological Crystalography*, vol. 66, no. 3, pp. 304-308, 2010.

[489] W. D. Grant and S. Heaphy, "Metagenomics and recovery of enzyme genes from alkaline saline environments," *Environmental Technology*, vol. 31, no. 10, pp. 1135-1143, 2010.

[490] A. Groisillier, C. Herve, A. Jeudy, E. Rebuffet, P. F. Pluchon, Y. Chevolot, D. Flament, C. Geslin, I. M. Morgado, D. Power, M. Branno, H. Moreau, G. Michel, C. Boyen, and M. Czjzek, "MARINE-EXPRESS: taking advantage of high throughput cloning and expression strategies for the post-genomic analysis of marine organisms," *Microbial Cell Factories*, vol. 9, p. 45, 2010.

[491] L. J. Happonen, P. Redder, X. Peng, L. J. Reigstad, D. Prangishvili, and S. J. Butcher, "Familial relationships in hyperthermo- and acidophilic archaeal viruses," *Journal of Virology*, vol. 84, no. 9, pp. 4747-4754, 2010.

[492] I. U. Heinemann, D. Soll, and L. Randau, "Transfer RNA processing in archaea: unusual pathways and enzymes," *FEBS Letters*, vol. 584, no. 2, pp. 303-309, 2010.

[493] W. P. Inskeep, D. B. Rusch, Z. J. Jay, M. J. Herrgard, M. A. Kozubal, T. H. Richardson, R. E. Macur, N. Hamamura, R. Jennings, B. W. Fouke, A. L. Reysenbach, F. Roberto, M. Young, A. Schwartz, E. S. Boyd, J. H. Badger, E. J. Mathur, A. C. Ortmann, M. Bateson, G. Geesey, and M. Frazier, "Metagenomes from high-temperature chemotrophic systems reveal geochemical controls on microbial community structure and function," *PLoS One*, vol. 5, no. 3, p. e9773, 2010.

[494] S. Jacquet, T. Miki, R. Noble, P. Peduzzi, and S. Wilhelm, "Viruses in aquatic ecosystems: important advancements of the last 20 years and prospects for the future in the field of microbial oceanography and limnology," *Advances in Oceanography and Limnology*, vol. 1, no. 1, pp. 97-141, 2010.

[495] M. Jalasvuori, A. Pawlowski, and J. K. Bamford, "A unique group of virus-related, genome-integrating elements found solely in the bacterial family *Thermaceae* and the archaeal family *Halobacteriaceae*," *Journal of Bacteriology*, vol. 192, no. 12, pp. 3231-3234, 2010.

[496] F. V. Karginov and G. J. Hannon, "The CRISPR system: small RNA-guided defense in bacteria and archaea," *Molecular Cell*, vol. 37, no. 1, pp. 7-19, 2010.

[497] R. Khayat, C. Y. Fu, A. C. Ortmann, M. J. Young, and J. E. Johnson, "The architecture and chemical stability of the archaeal *Sulfolobus* turreted icosahedral virus," *Journal of Virology*, vol. 84, no. 18, pp. 9575-9583, 2010.

[498] A. Klieve, *Reducing emissions from livestock research program: archaeaphage therapy to control rumen methanogens*, Meat & Livestock Australia, North Sydney, 2010.

[499] E. V. Koonin, "The wonder world of microbial viruses," *Expert Review of Anti-Infective Therapy*, vol. 8, no. 10, pp. 1097-1099, 2010.

[500] K. J. Koudelka and M. Manchester, "Chemically modified viruses: principles and applications," *Curr Opin Chem Biol*, vol. 14, no. 6, pp. 810-817, 2010.

[501] D. M. Kristensen, A. R. Mushegian, V. V. Dolja, and E. V. Koonin, "New dimensions of the virus world discovered through metagenomics," *Trends in Microbiology*, vol. 18, no. 1, pp. 11-19, 2010.

[502] M. Krupovic and D. H. Bamford, "Putative prophages related to lytic tailless marine dsDNA phage PM2 are widespread in the genomes of aquatic bacteria," *BMC Genomics*, vol. 8, p. 236, 2007.

[503] M. Krupovic and D. H. Bamford, "Order to the viral universe," *Journal of Virology*, vol. 84, no. 24, pp. 12476-12479, 2010.

[504] M. Krupovic, S. Gribaldo, D. H. Bamford, and P. Forterre, "The evolutionary history of archaeal MCM helicases: a case study of vertical evolution combined with hitchhiking of mobile genetic elements," *Molecular Biology and Evolution*, vol. 27, no. 12, pp. 2716-2732, 2010.

[505] M. Krupovic, P. Forterre, and D. H. Bamford, "Comparative analysis of the mosaic genomes of tailed archaeal viruses and proviruses suggests common themes for virion architecture and assembly with tailed viruses of bacteria," *Journal of Molecular Biology*, vol. 397, no. 1, pp. 144-160, 2010.

[506] H. Liesegang, A. K. Kaster, A. Wiezer, M. Goenrich, A. Wollherr, H. Seedorf, G. Gottschalk, and R. K. Thauer, "Complete genome sequence of *Methanothermobacter marburgensis*, a methanoarchaeon model organism," *Journal of Bacteriology*, vol. 192, no. 21, pp. 5850-5851, 2010.

[507] D. Lundin, S. Gribaldo, E. Torrents, B. M. Sjoberg, and A. M. Poole, "Ribonucleotide reduction - horizontal transfer of a required function spans all three domains," *BMC Evolutionary Biology*, vol. 10, p. 383, 2010.

[508] Y. Ma, E. A. Galinski, W. D. Grant, A. Oren, and A. Ventosa, "Halophiles 2010: life in saline environments," *Applied and Environmental Microbiology*, vol. 76, no. 21, pp. 6971-6981, 2010.

[509] L. A. Marraffini and E. J. Sontheimer, "Self versus non-self discrimination during CRISPR RNA-directed immunity," *Nature*, vol. 463, no. 7280, pp. 568-571, 2010.

[510] L. A. Marraffini and E. J. Sontheimer, "CRISPR interference: RNA-directed adaptive immunity in bacteria and archaea," *Nature Reviews Genetics*, vol. 11, no. 3, pp. 181-190, 2010.

[511] S. K. Menon, B. J. Eilers, M. J. Young, and C. M. Lawrence, "The crystal structure of D212 from sulfolobus spindle-shaped virus ragged hills reveals a new member of the PD-(D/E)XK nuclease superfamily," *Journal of Virology*, vol. 84, no. 12, pp. 5890-5897, 2010.

[512] T. Mochizuki, T. Yoshida, R. Tanaka, P. Forterre, Y. Sako, and D. Prangishvili, "Diversity of viruses of the hyperthermophilic archaeal genus *Aeropyrum*, and isolation of the *Aeropyrum pernix* bacilliform virus 1, APBV1, the first representative of the family *Clavaviridae*," *Virology*, vol. 402, no. 2, pp. 347-354, 2010.

[513] M. K. Pietilä, S. Laurinavicius, J. Sund, E. Roine, and D. H. Bamford, "The single-stranded DNA genome of novel archaeal virus *Halorubrum* pleomorphic virus 1 is enclosed in the envelope decorated with glycoprotein spikes," *Journal of Virology*, vol. 84, no. 2, pp. 788-798, 2010.

[514] T. E. Quax, M. Krupovic, S. Lucas, P. Forterre, and D. Prangishvili, "The *Sulfolobus* rod-shaped virus 2 encodes a prominent structural component of the unique virion release system in Archaea," *Virology*, vol. 404, no. 1, pp. 1-4, 2010.

[515] D. Raoult, "Giant viruses from amoeba in a post-Darwinist viral world," *Intervirology*, vol. 53, no. 5, pp. 251-253, 2010.

[516] D. Raoult and M. Boyer, "Amoebae as genitors and reservoirs of giant viruses," *Intervirology*, vol. 53, no. 5, pp. 321-329, 2010.

[517] E. Roine, P. Kukkaro, L. Paulin, S. Laurinavicius, A. Domanska, P. Somerharju, and D. H. Bamford, "New, closely related haloarchaeal viral elements with different nucleic acid types," *Journal of Virology*, vol. 84, no. 7, pp. 3682-3689, 2010.

[518] G. Ruprich-Robert and P. Thuriaux, "Non-canonical DNA transcription enzymes and the conservation of two-barrel RNA polymerases," *Nucleic Acids Research*, vol. 38, no. 14, pp. 4559-4569, 2010.

[519] F. Santos, P. Yarza, V. Parro, C. Briones, and J. Anton, "The metavirome of a hypersaline environment," *Environmental Microbiology*, vol. 12, no. 11, pp. 2965-2976, 2010.

[520] J. C. Snyder, M. M. Bateson, M. Lavin, and M. J. Young, "Use of cellular CRISPR (clusters of regularly interspaced short palindromic repeats) spacer-based microarrays for detection of viruses in environmental samples," *Applied and Environmental Microbiology*, vol. 76, no. 21, pp. 7251-7258, 2010.

[521] N. Soler, E. Marguet, D. Cortez, N. Desnoues, J. Keller, T. H. van, G. Sezonov, and P. Forterre, "Two novel families of plasmids from hyperthermophilic archaea encoding new families of replication proteins," *Nucleic Acids Research*, vol. 38, no. 15, pp. 5088-5104, 2010.

[522] V. A. Sorokin, M. S. Gelfand, and I. I. Artamonova, "Evolutionary dynamics of clustered irregularly interspaced short palindromic repeat systems in the ocean metagenome," *Applied and Environmental Microbiology*, vol. 76, no. 7, pp. 2136-2144, 2010.

[523] N. F. Steinmetz, M. E. Mertens, R. E. Taurog, J. E. Johnson, U. Commandeur, R. Fischer, and M. Manchester, "Potato virus X as a novel platform for potential biomedical applications," *Nano Letters*, vol. 10, no. 1, pp. 305-312, 2010.

[524] S. Sun, S. B. La, V. D. Bowman, C. M. Ryan, J. P. Whitelegge, D. Raoult, and M. G. Rossmann, "Structural studies of the Sputnik virophage," *Journal of Virology*, vol. 84, no. 2, pp. 894-897, 2010.

[525] J. Tie, S. Uchigasaki, T. Haseba, Y. Ohno, I. Isahai, and S. Oshida, "Direct and rapid PCR amplification using digested tissues for the diagnosis of drowning," *Electrophoresis*, vol. 31, no. 14, pp. 2411-2415, 2010.

[526] G. Tremberger, Jr., V. Gallardo, C. Espinoza, T. Holden, N. Gadura, E. Cheung, P. Schneider, D. Lieberman, and T. Cheung, "Archaeon and archaeal virus diversity classification via sequence entropy and fractal dimension," in *Instruments, Methods, and Missions for Astrobiology XIII*, R. B. Hoover, G. V. Levin, A. Y. Rozanov, and P. C. Davies, Eds. 2010.

[527] J. L. van Etten, J. R. Gurnon, G. M. Yanai-Balser, D. D. Dunigan, and M. V. Graves, "Chlorella viruses encode most, if not all, of the machinery to glycosylate their glycoproteins independent of the endoplasmic reticulum and Golgi," *Biochimica et Biophysica Acta*, vol. 1800, no. 2, pp. 152-159, 2010.

[528] L. P. Villarreal and G. Witzany, "Viruses are essential agents within the roots and stem of the tree of life," *Journal of Theoretical Biology*, vol. 262, no. 4, pp. 698-710, 2010.

[529] E. A. Waligora, D. M. Ramsey, E. E. Pryor, Jr., H. Lu, T. Hollis, G. P. Sloan, R. Deora, and D. J. Wozniak, "AmrZ beta-sheet residues are essential for DNA binding and transcriptional control of *Pseudomonas aeruginosa* virulence genes," *Journal of Bacteriology*, vol. 192, no. 20, pp. 5390-5401, 2010.

[530] A. D. Walters and J. P. Chong, "An archaeal order with multiple minichromosome maintenance genes," *Microbiology (Reading)*, vol. 156, no. 5, pp. 1405-1414, 2010.

[531] J. Walther, P. Sierocinski, and J. van der Oost, "Hot transcriptomics," *Archaea*, vol. 2010, p. 897585, 2011.

[532] Z. Wang and N. Goldenfeld, "Fixed points and limit cycles in the population dynamics of lysogenic viruses and their hosts," *Physical Review E, Statistical, Nonlinear, and Soft Matter Physics*, vol. 82, no. 1 Pt 1, p. 011918, 2010.

[533] D. Wei and X. Zhang, "Proteomic analysis of interactions between a deep-sea thermophilic bacteriophage and its host at high temperature," *Journal of Virology*, vol. 84, no. 5, pp. 2365-2373, 2010.

[534] W. H. Wilson and D. Schroeder, "Sequencing and characterization of virus genomes," in *Manual of Aquatic Viral Ecology*, S. W. Wilhelm, M. G. Weinbauer, and C. A. Suttle, Eds. pp. 134-144, ASLO, 2010.

[535] C. Winter, T. Bouvier, M. G. Weinbauer, and T. F. Thingstad, "Trade-offs between competition and defense specialists among unicellular planktonic organisms: the "killing the winner" hypothesis revisited," *Microbiology and Molecular Biology Reviews*, vol. 74, no. 1, pp. 42-57, 2010.

[536] M. Zaparty, D. Esser, S. Gertig, P. Haferkamp, T. Kouril, A. Manica, T. K. Pham, J. Reimann, K. Schreiber, P. Sierocinski, D. Teichmann, W. M. van, J. M. von, P. Wieloch, S. V. Albers, A. J. Driessen, H. P. Klenk, C. Schleper, D. Schomburg, J. van der Oost, P. C. Wright, and B. Siebers, ""Hot standards" for the thermoacidophilic archaeon *Sulfolobus solfataricus*," *Extremophiles*, vol. 14, no. 1, pp. 119-142, 2010.

[537] J. Zhang, T. Kasciukovic, and M. F. White, "The CRISPR associated protein Cas4 Is a 5' to 3' DNA exonuclease with an iron-sulfur cluster," *PLoS One*, vol. 7, no. 10, p. e47232, 2012.

[538] M. Zourob and S. Ripp, "Bacteriophage-based biosensors," in *Recognition Receptors in Biosensors*, M. Zourob, Ed., pp. 415-448, Springer Science, 2010.

[539] A. Abroi and J. Gough, "Are viruses a source of new protein folds for organisms? - Virosphere structure space and evolution," *Bioessays*, vol. 33, no. 8, pp. 626-635, 2011.

[540] S. V. Albers and B. H. Meyer, "The archaeal cell envelope," *Nature Reviews Microbiology*, vol. 9, no. 6, pp. 414-426, 2011.

[541] R. E. Anderson, W. J. Brazelton, and J. A. Baross, "Using CRISPRs as a metagenomic tool to identify microbial hosts of a diffuse flow hydrothermal vent viral assemblage," *FEMS Microbiology Ecology*, vol. 77, no. 1, pp. 120-133, 2011.

[542] A. Bertin, F. M. de, and L. Letellier, "Bacteriophage-host interactions leading to genome internalization," *Current Opinion in Microbiology*, vol. 14, no. 4, pp. 492-496, 2011.

[543] Y. Bettarel, T. Bouvier, C. Bouvier, C. Carre, A. Desnues, I. Domaizon, S. Jacquet, A. Robin, and T. Sime-Ngando, "Ecological traits of planktonic viruses and prokaryotes along a full-salinity gradient," *FEMS Microbiology Ecology*, vol. 76, no. 2, pp. 360-372, 2011.

[544] B. S. Blumberg, "Astrobiology, space and the future age of discovery," *Philosophical Transactions Series A, Mathematical, Physical, And Engineering Sciences*, vol. 369, no. 1936, pp. 508-515, 2011.

[545] M. Breitbart, "Marine viruses: truth or dare," *Annual Review of Marine Science*, vol. 4, pp. 425-448, 2012.

[546] A. Brodt, M. N. Lurie-Weinberger, and U. Gophna, "CRISPR loci reveal networks of gene exchange in archaea," *Biology Direct*, vol. 6, no. 1, p. 65, 2011.

[547] R. Cavicchioli, "Archaea — timeline of the third domain," *Nature Reviews Microbiology*, vol. 9, no. 1, pp. 51-61, 2011.

[548] M. R. Clokie, A. D. Millard, A. V. Letarov, and S. Heaphy, "Phages in nature," *Bacteriophage*, vol. 1, no. 1, pp. 31-45, 2011.

[549] P. Contursi, K. D'Ambrosio, L. Pirone, E. Pedone, T. Aucelli, Q. She, S. G. De, and S. Bartolucci, "C68 from the *Sulfolobus islandicus* plasmid-virus pSSVx is a novel member of the AbrB-like transcription factor family," *Biochemical Journal*, vol. 435, no. 1, pp. 157-166, 2011.

[550] M. DeYoung, M. Thayer, J. van der Oost, and K. M. Stedman, "Growth phase-dependent gene regulation *in vivo* in *Sulfolobus solfataricus*," *FEMS Microbiology Letters*, vol. 321, no. 2, pp. 92-99, 2011.

[551] M. L. Dyall-Smith, F. Pfeiffer, K. Klee, P. Palm, K. Gross, S. C. Schuster, M. Rampp, and D. Oesterhelt, "*Haloquadratum walsbyi*: limited diversity in a global pond," *PLoS One*, vol. 6, no. 6, p. e20968, 2011.

[552] S. Erdmann, U. Scheele, and R. A. Garrett, "AAA ATPase p529 of *Acidianus* two-tailed virus ATV and host receptor recognition," *Virology*, vol. 421, no. 1, pp. 61-66, 2011.

[553] P. Forterre, "Manipulation of cellular syntheses and the nature of viruses: the virocell concept," *Comptes Rendus Chimie*, vol. 14, no. 4, pp. 392-399, 2011.

[554] P. Forterre, "A new fusion hypothesis for the origin of Eukarya: better than previous ones, but probably also wrong," *Research in Microbiology*, vol. 162, no. 1, pp. 77-91, 2011.

[555] C. Y. Fu and J. E. Johnson, "Viral life cycles captured in three-dimensions with electron microscopy tomography," *Current Opinion in Virology*, vol. 1, no. 2, pp. 125-133, 2011.

[556] A. F. Gardner, C. Guan, and W. E. Jack, "Biochemical characterization of a structure-specific resolving enzyme from *Sulfolobus islandicus* rod-shaped virus 2," *PLoS One*, vol. 6, no. 8, p. e23668, 2011.

[557] A. F. Gardner, D. Prangishvili, and W. E. Jack, "Characterization of *Sulfolobus islandicus* rod-shaped virus 2 gp19, a single-strand specific endonuclease," *Extremophiles*, vol. 15, no. 5, pp. 619-624, 2011.

[558] E. E. Gill and F. S. Brinkman, "The proportional lack of archaeal pathogens: Do viruses/phages hold the key?," *Bioessays*, vol. 33, no. 4, pp. 248-254, 2011.

[559] M. Gonnet, G. Erauso, D. Prieur, and R. M. Le, "pAMT11, a novel plasmid isolated from a *Thermococcus sp.* strain closely related to the virus-like integrated element TKV1 of the *Thermococcus kodakaraensis* genome," *Research in Microbiology*, vol. 162, no. 2, pp. 132-143, 2011.

[560] A. Goulet, J. Lai-Kee-Him, D. Veesler, I. Auzat, G. Robin, D. A. Shepherd, A. E. Ashcroft, E. Richard, J. Lichiere, P. Tavares, C. Cambillau, and P. Bron, "The opening of the SPP1 bacteriophage tail, a prevalent mechanism in Gram-positive-infecting siphophages," *Journal of Biological Chemistry*, vol. 286, no. 28, pp. 25397-25405, 2011.

[561] S. Gudbergsdottir, L. Deng, Z. Chen, J. V. Jensen, L. R. Jensen, Q. She, and R. A. Garrett, "Dynamic properties of the *Sulfolobus* CRISPR/Cas and CRISPR/Cmr systems when challenged with vector-borne viral and plasmid genes and protospacers," *Molecular Microbiology*, vol. 79, no. 1, pp. 35-49, 2011.

[562] J. Heinemann, W. S. Maaty, G. H. Gauss, N. Akkaladevi, S. K. Brumfield, V. Rayaprolu, M. J. Young, C. M. Lawrence, and B. Bothner, "Fossil record of an archaeal HK97-like provirus," *Virology*, vol. 417, no. 2, pp. 362-368, 2011.

[563] L. Herrero-Uribe, "Viruses, definitions and reality," *Revista de Biologica Tropical*, vol. 59, no. 3, pp. 993-998, 2011.

[564] A. Hirata, T. Kitajima, and H. Hori, "Cleavage of intron from the standard or non-standard position of the precursor tRNA by the splicing endonuclease of *Aeropyrum pernix*, a hyper-thermophilic Crenarchaeon, involves a novel RNA recognition site in the Crenarchaea specific loop," *Nucleic Acids Research*, vol. 39, no. 21, pp. 9376-9389, 2011.

[565] S. Ishino, S. Fujino, H. Tomita, H. Ogino, K. Takao, H. Daiyasu, T. Kanai, H. Atomi, and Y. Ishino, "Biochemical and genetical analyses of the three *mcm* genes from the hyperthermophilic archaeon, *Thermococcus kodakarensis*," *Genes to Cells*, vol. 16, no. 12, pp. 1176-1189, 2011.

[566] K. F. Jarrell, A. D. Walters, C. Bochiwal, J. M. Borgia, T. Dickinson, and J. P. Chong, "Major players on the microbial stage: why archaea are important," *Microbiology (Reading)*, vol. 157, no. 4, pp. 919-936, 2011.

[567] J. Jorda and T. O. Yeates, "Widespread disulfide bonding in proteins from thermophilic archaea," *Archaea*, vol. 2011, p. 409156, 2011.

[568] J. Kan, S. Clingenpeel, R. E. Macur, W. P. Inskeep, D. Lovalvo, J. Varley, Y. Gorby, T. R. McDermott, and K. Nealson, "Archaea in Yellowstone Lake," *ISME J*, vol. 5, no. 11, pp. 1784-1795, 2011.

[569] A. K. Kaster, M. Goenrich, H. Seedorf, H. Liesegang, A. Wollherr, G. Gottschalk, and R. K. Thauer, "More than 200 genes required for methane formation from H_2_ and CO_2_ and energy conservation are present in *Methanothermobacter marburgensis* and *Methanothermobacter thermautotrophicus*," *Archaea*, vol. 2011, p. 973848, 2011.

[570] D. Kazlauskas and C. Venclovas, "Computational analysis of DNA replicases in double-stranded DNA viruses: relationship with the genome size," *Nucleic Acids Research*, vol. 39, no. 19, pp. 8291-8305, 2011.

[571] D. M. Kristensen, X. Cai, and A. Mushegian, "Evolutionarily conserved orthologous families in phages are relatively rare in their prokaryotic hosts," *Journal of Bacteriology*, vol. 193, no. 8, pp. 1806-1814, 2011.

[572] M. Krupovic and D. H. Bamford, "Double-stranded DNA viruses: 20 families and only five different architectural principles for virion assembly," *Current Opinion in Virology*, vol. 1, no. 2, pp. 118-124, 2011.

[573] M. Krupovic and V. Cvirkaite-Krupovic, "Virophages or satellite viruses?," *Nature Reviews Microbiology*, vol. 9, no. 11, pp. 762-763, 2011.

[574] M. Krupovic and P. Forterre, "*Microviridae* goes temperate: microvirus-related proviruses reside in the genomes of *Bacteroidetes*," *PLoS One*, vol. 6, no. 5, p. e19893, 2011.

[575] M. Krupovic, A. Spang, S. Gribaldo, P. Forterre, and C. Schleper, "A thaumarchaeal provirus testifies for an ancient association of tailed viruses with archaea," *Biochemical Society Transactions*, vol. 39, no. 1, pp. 82-88, 2011.

[576] M. Krupovic, D. Prangishvili, R. W. Hendrix, and D. H. Bamford, "Genomics of bacterial and archaeal viruses: dynamics within the prokaryotic virosphere," *Microbiology and Molecular Biology Reviews*, vol. 75, no. 4, pp. 610-635, 2011.

[577] M. Laganeckas, M. Margelevicius, and C. Venclovas, "Identification of new homologs of PD-(D/E)XK nucleases by support vector machines trained on data derived from profile-profile alignments," *Nucleic Acids Research*, vol. 39, no. 4, pp. 1187-1196, 2011.

[578] C. D. Litchfield, "Potential for industrial products from the halophilic Archaea," *Journal of Industrial Microbiology and Biotechnology*, vol. 38, no. 10, pp. 1635-1647, 2011.

[579] H. Liu, Z. Wu, M. Li, F. Zhang, H. Zheng, J. Han, J. Liu, J. Zhou, S. Wang, and H. Xiang, "Complete genome sequence of *Haloarcula hispanica*, a model haloarchaeon for studying genetics, metabolism, and virus-host interaction," *Journal of Bacteriology*, vol. 193, no. 21, pp. 6086-6087, 2011.

[580] K. S. Makarova, Y. I. Wolf, S. Snir, and E. V. Koonin, "Defense islands in bacterial and archaeal genomes and prediction of novel defense systems," *Journal of Bacteriology*, vol. 193, no. 21, pp. 6039-6056, 2011.

[581] A. Manica, Z. Zebec, D. Teichmann, and C. Schleper, "In vivo activity of CRISPR-mediated virus defence in a hyperthermophilic archaeon," *Molecular Microbiology*, vol. 80, no. 2, pp. 481-491, 2011.

[582] T. Mochizuki, Y. Sako, and D. Prangishvili, "Provirus induction in hyperthermophilic archaea: characterization of *Aeropyrum pernix* spindle-shaped virus 1 and *Aeropyrum pernix* ovoid virus 1," *Journal of Bacteriology*, vol. 193, no. 19, pp. 5412-5419, 2011.

[583] M. Oke, M. Kerou, H. Liu, X. Peng, R. A. Garrett, D. Prangishvili, J. H. Naismith, and M. F. White, "A dimeric Rep protein initiates replication of a linear archaeal virus genome: implications for the Rep mechanism and viral replication," *Journal of Virology*, vol. 85, no. 2, pp. 925-931, 2011.

[584] C. Pan, C. R. Fischer, D. Hyatt, B. P. Bowen, R. L. Hettich, and J. F. Banfield, "Quantitative tracking of isotope flows in proteomes of microbial communities," *Molecular and Cellular Proteomics*, vol. 10, no. 4, p. M110, 2011.

[585] M. Pina, A. Bize, P. Forterre, and D. Prangishvili, "The archeoviruses," *FEMS Microbiology Reviews*, vol. 35, no. 6, pp. 1035-1054, 2011.

[586] D. Prangishvili, "Viruses of the Archaea: a view on the viral world from the perspective of hyperthermophilic viruses," *Bulletin of the Georgian National Academy of Sciences*, vol. 5, no. 2, pp. 118-126, 2011.

[587] D. Prangishvili and T. E. Quax, "Exceptional virion release mechanism: one more surprise from archaeal viruses," *Current Opinion in Microbiology*, vol. 14, no. 3, pp. 315-320, 2011.

[588] T. E. Quax, S. Lucas, J. Reimann, G. Pehau-Arnaudet, M. C. Prevost, P. Forterre, S. V. Albers, and D. Prangishvili, "Simple and elegant design of a virion egress structure in Archaea," *Proceedings of the National Academy of Sciences, USA*, vol. 108, no. 8, pp. 3354-3359, 2011.

[589] F. Rohwer and M. Youle, "Consider something viral in your research ," *Nature Reviews Microbiology*, vol. 9, no. 5, pp. 308-309, 2011.

[590] S. G. Sanmukh, W. N. Paunikar, T. K. Ghosh, and T. Chakrabarti, "Structural & functional prediction of hypothetical proteins in bacteriophages against halophilic bacteial - an *in silico* approach," *International Journal of Pharma and Bio Sciences*, vol. 2, no. 2, p. B-61-B-70, 2011.

[591] F. Santos, M. Moreno-Paz, I. Meseguer, C. Lopez, R. Rossello-Mora, V. Parro, and J. Anton, "Metatranscriptomic analysis of extremely halophilic viral communities," *ISME Journal*, vol. 5, no. 10, pp. 1621-1633, 2011.

[592] U. Scheele, S. Erdmann, E. J. Ungewickell, C. Felisberto-Rodrigues, M. Ortiz-Lombardia, and R. A. Garrett, "Chaperone role for proteins p618 and p892 in the extracellular tail development of *Acidianus* two-tailed virus," *Journal of Virology*, vol. 85, no. 10, pp. 4812-4821, 2011.

[593] V. Sejian, R. Lal, J. Lakritz, and T. Ezeji, "Measurement and prediction of enteric methane emission," *International Journal of Biometeorology*, vol. 55, no. 1, pp. 1-16, 2011.

[594] P. Serwer, "Proposed ancestors of phage nucleic acid packaging motors (and cells)," *Viruses*, vol. 3, no. 7, pp. 1249-1280, 2011.

[595] L. Guo, K. Brugger, C. Liu, S. A. Shah, H. Zheng, Y. Zhu, S. Wang, R. K. Lillestol, L. Chen, J. Frank, D. Prangishvili, L. Paulin, Q. She, L. Huang, and R. A. Garrett, "Genome analyses of Icelandic strains of *Sulfolobus islandicus*: model organisms for genetic and virus-host interaction studies," *Journal of Bacteriology*, vol. 193, no. 7, pp. 1672-1680, 2011.

[596] S. A. Shah and R. A. Garrett, "CRISPR/Cas and Cmr modules, mobility and evolution of adaptive immune systems," *Research in Microbiology*, vol. 162, no. 1, pp. 27-38, 2011.

[597] B. Siebers, M. Zaparty, G. Raddatz, B. Tjaden, S. V. Albers, S. D. Bell, F. Blombach, A. Kletzin, N. Kyrpides, C. Lanz, A. Plagens, M. Rampp, A. Rosinus, J. M. von, K. S. Makarova, H. P. Klenk, S. C. Schuster, and R. Hensel, "The complete genome sequence of *Thermoproteus tenax*: a physiologically versatile member of the *Crenarchaeota*," *PLoS One*, vol. 6, no. 10, p. e24222, 2011.

[598] P. L. Siering, J. M. Clarke, and M. S. Wilson, "Geochemical and biological diversity of acidic, hot springs in Lassen Volcanic National Park," *Geomicrobiology Journal*, vol. 23, pp. 129-141, 2006.

[599] T. Sime-Ngando, S. Lucas, A. Robin, K. P. Tucker, J. Colombet, Y. Bettarel, E. Desmond, S. Gribaldo, P. Forterre, M. Breitbart, and D. Prangishvili, "Diversity of virus-host systems in hypersaline Lake Retba, Senegal," *Environmental Microbiology*, vol. 13, no. 8, pp. 1956-1972, 2011.

[600] J. C. Snyder and M. J. Young, "Advances in understanding archaea-virus interactions in controlled and natural environments," *Current Opinion in Microbiology*, vol. 14, no. 4, pp. 497-503, 2011.

[601] J. C. Snyder and M. J. Young, "Potential role of cellular ESCRT proteins in the STIV life cycle," *Biochemical Society Transactions*, vol. 39, no. 1, pp. 107-110, 2011.

[602] J. C. Snyder, S. K. Brumfield, N. Peng, Q. She, and M. J. Young, "*Sulfolobus* turreted icosahedral virus c92 protein responsible for the formation of pyramid-like cellular lysis structures," *Journal of Virology*, vol. 85, no. 13, pp. 6287-6292, 2011.

[603] N. Soler, M. Gaudin, E. Marguet, and P. Forterre, "Plasmids, viruses and virus-like membrane vesicles from Thermococcales," *Biochemical Society Transactions*, vol. 39, no. 1, pp. 36-44, 2011.

[604] J. C. Snyder, B. Bolduc, M. M. Bateson, and M. J. Young, "The prevalence of STIV c92-like Proteins in acidic thermal environments," *Advances in Virology*, vol. 2011, p. 650930, 2011.

[605] M. Tamakoshi, A. Murakami, M. Sugisawa, K. Tsuneizumi, S. Takeda, T. Saheki, T. Izumi, T. Akiba, K. Mitsuoka, H. Toh, A. Yamashita, F. Arisaka, M. Hattori, T. Oshima, and A. Yamagishi, "Genomic and proteomic characterization of the large Myoviridae bacteriophage varphiTMA of the extreme thermophile *Thermus thermophilus*," *Bacteriophage*, vol. 1, no. 3, pp. 152-164, 2011.

[606] M. P. Terns and R. M. Terns, "CRISPR-based adaptive immune systems," *Current Opinion in Microbiology*, vol. 14, no. 3, pp. 321-327, 2011.

[607] R. L. V. Thurber and A. M. S. Correa, "Viruses of reef-building scleractinian corals," *Journal of Experimental Marine Biology and Ecology*, vol. 408, pp. 102-113, 2011.

[608] S. N. Trojet, A. Caumont-Sarcos, E. Perrody, A. M. Comeau, and H. M. Krisch, "The gp38 adhesins of the T4 superfamily: a complex modular determinant of the phage's host specificity," *Genome Biology and Evolution*, vol. 3, pp. 674-686, 2011.

[609] M. Vesteg and J. Krajcovic, "The falsifiability of the models for the origin of eukaryotes," *Current Genetics*, vol. 57, no. 6, pp. 367-390, 2011.

[610] L. P. Villarreal, "Viral ancestors of antiviral systems," *Viruses*, vol. 3, no. 10, pp. 1933-1958, 2011.

[611] G. R. Visweswaran, B. W. Dijkstra, and J. Kok, "Murein and pseudomurein cell wall binding domains of bacteria and archaea--a comparative view," *Applied Microbiology and Biotechnology*, vol. 92, no. 5, pp. 921-928, 2011.

[612] G. R. Visweswaran, B. W. Dijkstra, and J. Kok, "A minimum of three motifs is essential for optimal binding of pseudomurein cell wall-binding domain of *Methanothermobacter thermautotrophicus*," *PLoS One*, vol. 6, no. 6, p. e21582, 2011.

[613] J. F. Wirth, J. C. Snyder, R. A. Hochstein, A. C. Ortmann, D. A. Willits, T. Douglas, and M. J. Young, "Development of a genetic system for the archaeal virus *Sulfolobus* turreted icosahedral virus (STIV)," *Virology*, vol. 415, no. 1, pp. 6-11, 2011.

[614] X. Y. You, C. Liu, S. Y. Wang, C. Y. Jiang, S. A. Shah, D. Prangishvili, Q. She, S. J. Liu, and R. A. Garrett, "Genomic analysis of *Acidianus hospitalis* W1 a host for studying crenarchaeal virus and plasmid life cycles," *Extremophiles*, vol. 15, no. 4, pp. 487-497, 2011.

[615] A. P. Aalto, D. Bitto, J. J. Ravantti, D. H. Bamford, J. T. Huiskonen, and H. M. Oksanen, "Snapshot of virus evolution in hypersaline environments from the characterization of a membrane-containing *Salisaeta* icosahedral phage 1," *Proceedings of the National Academy of Sciences, USA*, vol. 109, no. 18, pp. 7079-7084, 2012.

[616] N. G. Abrescia, D. H. Bamford, J. M. Grimes, and D. I. Stuart, "Structure unifies the viral universe," *Annu Rev Biochem*, vol. 81, pp. 795-822, 2012.

[617] H. W. Ackermann and K. L. Tiekotter, "Murphy's law-if anything can go wrong, it will: Problems in phage electron microscopy," *Bacteriophage*, vol. 2, no. 2, pp. 122-129, 2012.

[618] H. W. Ackermann, "Bacteriophage electron microscopy," *Advances in Virus Research*, vol. 82, pp. 1-32, 2012.

[619] H. W. Ackermann and D. Prangishvili, "Prokaryote viruses studied by electron microscopy," *Archives of Virology*, vol. 157, no. 10, pp. 1843-1849, 2012.

[620] N. S. Atanasova, E. Roine, A. Oren, D. H. Bamford, and H. M. Oksanen, "Global network of specific virus-host interactions in hypersaline environments," *Environmental Microbiology*, vol. 14, no. 2, pp. 426-440, 2012.

[621] J. K. Blackwood, N. J. Rzechorzek, A. S. Abrams, J. D. Maman, L. Pellegrini, and N. P. Robinson, "Structural and functional insights into DNA-end processing by the archaeal HerA helicase-NurA nuclease complex," *Nucleic Acids Research*, vol. 40, no. 7, pp. 3183-3196, 2012.

[622] B. Bolduc, D. P. Shaughnessy, Y. I. Wolf, E. V. Koonin, F. F. Roberto, and M. Young, "Identification of novel positive-strand RNA viruses by metagenomic analysis of archaea-dominated Yellowstone hot springs," *Journal of Virology*, vol. 86, no. 10, pp. 5562-5573, 2012.

[623] G. Borrel, J. Colombet, A. Robin, A. C. Lehours, D. Prangishvili, and T. Sime-Ngando, "Unexpected and novel putative viruses in the sediments of a deep-dark permanently anoxic freshwater habitat," *ISME Journal*, vol. 6, no. 11, pp. 2119-2127, 2012.

[624] I. Boujelben, P. Yarza, C. Almansa, J. Villamor, S. Maalej, J. Anton, and F. Santos, "Virioplankton community structure in Tunisian solar salterns," *Applied and Environmental Microbiology*, vol. 78, no. 20, pp. 7429-7437, 2012.

[625] R. M. Ceballos, C. D. Marceau, J. O. Marceau, S. Morris, A. J. Clore, and K. M. Stedman, "Differential virus host-ranges of the *Fuselloviridae* of hyperthermophilic Archaea: implications for evolution in extreme environments," *Frontiers in Microbiology*, vol. 3, p. 295, 2012.

[626] P. P. Chan, A. D. Holmes, A. M. Smith, D. Tran, and T. M. Lowe, "The UCSC Archaeal Genome Browser: 2012 update," *Nucleic Acids Research*, vol. 40, no. D1, p. D646-D652, 2012.

[627] S. Chen, R. E. Tulloss, Y. Liu, B. Feng, Z. Zhao, and Z. L. Yang, "Lateral gene transfer occurring in haloarchaea: an interpretative imitation study," *World Journal of Microbiology and Biotechnology*, vol. 28, no. 9, pp. 2913-2918, 2012.

[628] A. D. Dearborn, P. Laurinmaki, P. Chandramouli, C. M. Rodenburg, S. Wang, S. J. Butcher, and T. Dokland, "Structure and size determination of bacteriophage P2 and P4 procapsids: function of size responsiveness mutations," *Journal of Structural Biology*, vol. 178, no. 3, pp. 215-224, 2012.

[629] L. Deng, C. S. Kenchappa, X. Peng, Q. She, and R. A. Garrett, "Modulation of CRISPR locus transcription by the repeat-binding protein Cbp1 in *Sulfolobus*," *Nucleic Acids Research*, vol. 40, no. 6, pp. 2470-2480, 2012.

[630] C. Desnues, S. B. La, N. Yutin, G. Fournous, C. Robert, S. Azza, P. Jardot, S. Monteil, A. Campocasso, E. V. Koonin, and D. Raoult, "Provirophages and transpovirons as the diverse mobilome of giant viruses," *Proceedings of the National Academy of Sciences, USA*, vol. 109, no. 44, pp. 18078-18083, 2012.

[631] B. Dwivedi, R. Schmieder, D. B. Goldsmith, R. A. Edwards, and M. Breitbart, "PhiSiGns: an online tool to identify signature genes in phages and design PCR primers for examining phage diversity," *BMC Bioinformatics*, vol. 13, p. 37, 2012.

[632] B. J. Eilers, M. J. Young, and C. M. Lawrence, "The structure of an archaeal viral integrase reveals an evolutionarily conserved catalytic core yet supports a mechanism of DNA cleavage in trans," *Journal of Virology*, vol. 86, no. 15, pp. 8309-8313, 2012.

[633] J. B. Emerson, B. C. Thomas, K. Andrade, E. E. Allen, K. B. Heidelberg, and J. F. Banfield, "Dynamic viral populations in hypersaline systems as revealed by metagenomic assembly," *Applied and Environmental Microbiology*, vol. 78, no. 17, pp. 6309-6320, 2012.

[634] S. Erdmann and R. A. Garrett, "Selective and hyperactive uptake of foreign DNA by adaptive immune systems of an archaeon via two distinct mechanisms," *Molecular Microbiology*, vol. 85, no. 6, pp. 1044-1056, 2012.

[635] C. Felisberto-Rodrigues, S. Blangy, A. Goulet, G. Vestergaard, C. Cambillau, R. A. Garrett, and M. Ortiz-Lombardia, "Crystal structure of ATV*^ORF273^*, a new fold for a thermo- and acido-stable protein from the *Acidianus* two-tailed virus," *PLoS One*, vol. 7, no. 10, p. e45847, 2012.

[636] J. Filèe and M. Chandler, "Unpacking the baggage: origin and evolution of giant viruses," in *Viruses: Essential Agents of Life*, G. Witzany, Ed., pp. 203-216, Springer, Dordrecht, 2012.

[637] P. Forterre, N. Soler, M. Krupovic, E. Marguet, and H. W. Ackermann, "Fake virus particles generated by fluorescence microscopy," *Trends in Microbiology*, 2012.

[638] P. Forterre, "Darwin's goldmine is still open: variation and selection run the world," *Frontiers in Cellular and Infection Microbiology*, vol. 2, p. 106, 2012.

[639] C. Y. Fu and J. E. Johnson, "Structure and cell biology of archaeal virus STIV," *Current Opinion in Virology*, vol. 2, no. 2, pp. 122-127, 2012.

[640] I. Garcia-Heredia, A. B. Martin-Cuadrado, F. J. Mojica, F. Santos, A. Mira, J. Anton, and F. Rodriguez-Valera, "Reconstructing viral genomes from the environment using fosmid clones: the case of haloviruses," *PLoS One*, vol. 7, no. 3, p. e33802, 2012.

[641] M. Gaudin, E. Gauliard, S. Schouten, L. Houel-Renault, P. Lenormand, E. Marguet, and P. Forterre, "Hyperthermophilic archaea produce membrane vesicles that can transfer DNA," *Environmental Microbiology Reports*, vol. doi:10.1111/j.1758-2229.2012.00348.x, 2012.

[642] K. Georgiades and D. Raoult, "How microbiology helps define the rhizome of life," *Frontiers in Cellular and Infection Microbiology*, vol. 2, p. 60, 2012.

[643] U. Gophna and A. Brodt, "CRISPR/Cas systems in archaea: What array spacers can teach us about parasitism and gene exchange in the 3rd domain of life," *Mobile Genetic Elements*, vol. 2, no. 1, pp. 63-64, 2012.

[644] A. Gorlas, E. V. Koonin, N. Bienvenu, D. Prieur, and C. Geslin, "TPV1, the first virus isolated from the hyperthermophilic genus *Thermococcus*," *Environmental Microbiology*, vol. 14, no. 2, pp. 503-516, 2012.

[645] P. B. Hedlund, J. K. Cole, A. J. Williams, W. Hou, E. Zhou, W. Li, and H. Dong, "A review of the microbiology of the Rehai geothermal field in Tengchong, Yunnan Province, China," *Geoscience Frontiers*, vol. 3, no. 3, pp. 273-288, 2012.

[646] M. Henry and L. Debarbieux, "Tools from viruses: bacteriophage successes and beyond," *Virology*, 2012.

[647] L. Huang, "Unveiling the beauty of Archaea," *Science China Life Sciences*, vol. 55, no. 5, pp. 375-376, 2012.

[648] P. Hyman and S. T. Abedon, "Smaller fleas: viruses of microorganisms," *Scientifica*, vol. 2012, p. 734023, 2012.

[649] E. Iverson and K. Stedman, "A genetic study of SSV1, the prototypical fusellovirus," *Frontiers in Microbiology*, vol. 3, p. 200, 2012.

[650] S. T. Jaakkola, R. K. Penttinen, S. T. Vilen, M. Jalasvuori, G. Ronnholm, J. K. Bamford, D. H. Bamford, and H. M. Oksanen, "Closely related archaeal *Haloarcula hispanica* icosahedral viruses HHIV-2 and SH1 have nonhomologous genes encoding host recognition functions," *Journal of Virology*, vol. 86, no. 9, pp. 4734-4742, 2012.

[651] L. Kandiba, O. Aitio, J. Helin, Z. Guan, P. Permi, D. H. Bamford, J. Eichler, and E. Roine, "Diversity in prokaryotic glycosylation: an archaeal-derived N-linked glycan contains legionaminic acid," *Molecular Microbiology*, vol. 84, no. 3, pp. 578-593, 2012.

[652] R. Klein, N. Rossler, M. Iro, H. Scholz, and A. Witte, "Haloarchaeal myovirus φCh1 harbours a phase variation system for the production of protein variants with distinct cell surface adhesion specificities," *Molecular Microbiology*, vol. 83, no. 1, pp. 137-150, 2012.

[653] E. V. Koonin and V. V. Dolja, "Expanding networks of RNA virus evolution," *BMC Biology*, vol. 10, p. 54, 2012.

[654] D. M. Kristensen, A. S. Waller, T. Yamada, P. Bork, A. R. Mushegian, and E. V. Koonin, "Orthologous gene clusters and taxon signature genes for viruses of prokaryotes," *Journal of Bacteriology*, 2012.

[655] M. Krupovic, N. Peixeiro, M. Bettstetter, R. Rachel, and D. Prangishvili, "Archaeal tetrathionate hydrolase goes viral: secretion of a sulfur metabolism enzyme in the form of virus-like particles," *Applied and Environmental Microbiology*, vol. 78, no. 15, pp. 5463-5465, 2012.

[656] M. Krupovic, M. F. White, P. Forterre, and D. Prangishvili, "Postcards from the edge: structural genomics of archaeal viruses," *Advances in Virus Research*, vol. 82, pp. 33-62, 2012.

[657] A. S. Lang, O. Zhaxybayeva, and J. T. Beatty, "Gene transfer agents: phage-like elements of genetic exchange," *Nature Reviews Microbiology*, vol. 10, no. 7, pp. 472-482, 2012.

[658] Y. W. Lim, R. Schmieder, M. Haynes, D. Willner, M. Furlan, M. Youle, K. Abbott, R. Edwards, J. Evangelista, D. Conrad, and F. Rohwer, "Metagenomics and metatranscriptomics: Windows on CF-associated viral and microbial communities," *Journal of Cystic Fibrosis*, 2012.

[659] M. N. Lurie-Weinberger, M. Peeri, and U. Gophna, "Contribution of lateral gene transfer to the gene repertoire of a gut-adapted methanogen," *Genomics*, vol. 99, no. 1, pp. 52-58, 2012.

[660] W. S. Maaty, J. D. Steffens, J. Heinemann, A. C. Ortmann, B. D. Reeves, S. K. Biswas, E. A. Dratz, P. A. Grieco, M. J. Young, and B. Bothner, "Global analysis of viral infection in an archaeal model system," *Frontiers in Microbiology*, vol. 3, p. 411, 2012.

[661] W. S. Maaty, K. Selvig, S. Ryder, P. Tarlykov, J. K. Hilmer, J. Heinemann, J. Steffens, J. C. Snyder, A. C. Ortmann, N. Movahed, K. Spicka, L. Chetia, P. A. Grieco, E. A. Dratz, T. Douglas, M. J. Young, and B. Bothner, "Proteomic analysis of *Sulfolobus solfataricus* during *Sulfolobus* turreted icosahedral virus infection," *Journal of Proteome Research*, vol. 11, no. 2, pp. 1420-1432, 2012.

[662] A. Marchfelder, S. Fischer, J. Brendel, B. Stoll, L. K. Maier, D. Jager, D. Prasse, A. Plagens, R. A. Schmitz, and L. Randau, "Small RNAs for defence and regulation in archaea," *Extremophiles*, vol. 16, no. 5, pp. 685-696, 2012.

[663] A. V. Mardanov and N. V. Ravin, "The impact of genomics on research in diversity and evolution of archaea," *Biochemistry (Mosc )*, vol. 77, no. 8, pp. 799-812, 2012.

[664] M. Mentasti, N. K. Fry, B. Afshar, C. Palepou-Foxley, F. C. Naik, and T. G. Harrison, "Application of *Legionella pneumophila*-specific quantitative real-time PCR combined with direct amplification and sequence-based typing in the diagnosis and epidemiological investigation of Legionnaires' disease," *European Journal of Clinical Microbiology and Infectious Diseases*, vol. 31, no. 8, pp. 2017-2028, 2012.

[665] T. Mochizuki, M. Krupovic, G. Pehau-Arnaudet, Y. Sako, P. Forterre, and D. Prangishvili, "Archaeal virus with exceptional virion architecture and the largest single-stranded DNA genome," *Proceedings of the National Academy of Sciences, USA*, vol. 109, no. 33, pp. 13386-13391, 2012.

[666] A. Nasir, K. M. Kim, and G. Caetano-Anollés, "Viral Evolution: primordial cellular origins and late adaptation to parasitism," *Mobile Genetic Elements*, vol. 2, no. 5, pp. 1-6, 2012.

[667] S. Ng, M. R. Jafari, and R. Derda, "Bacteriophages and viruses as a support for organic synthesis and combinatorial chemistry," *ACS Chemical Biology*, vol. 7, no. 1, pp. 123-138, 2012.

[668] N. Peixeiro, J. Keller, B. Collinet, N. Leulliot, V. Campanacci, D. Cortez, C. Cambillau, K. R. Nitta, R. Vincentelli, P. Forterre, D. Prangishvili, G. Sezonov, and T. H. van, "Structure and function of AvtR, a novel transcriptional regulator from a hyperthermophilic archaeal lipothrixvirus," *Journal of Virology*, 2012.

[669] M. K. Pietila, N. S. Atanasova, H. M. Oksanen, and D. H. Bamford, "Modified coat protein forms the flexible spindle-shaped virion of haloarchaeal virus His1," *Environmental Microbiology*, 2012.

[670] M. K. Pietilä, N. S. Atanasova, V. Manole, L. Liljeroos, S. J. Butcher, H. M. Oksanen, and D. H. Bamford, "Virion architecture unifies globally distributed pleolipoviruses infecting halophilic archaea," *Journal of Virology*, vol. 86, no. 9, pp. 5067-5079, 2012.

[671] A. Plagens, B. Tjaden, A. Hagemann, L. Randau, and R. Hensel, "Characterization of the CRISPR/Cas subtype I-A system of the hyperthermophilic crenarchaeon *Thermoproteus tenax*," *Journal of Bacteriology*, vol. 194, no. 10, pp. 2491-2500, 2012.

[672] M. M. Poranen and D. H. Bamford, "Assebly of large icosahedral double-stranded RNA viruses," in *Viral Molecular Machine*, M. G. Rossmann and V. B. Rao, Eds. pp. 379-402, Spring Science, 2012.

[673] D. Prangishvili and M. Krupovic, "A new proposed taxon for double-stranded DNA viruses, the order "Ligamenvirales"," *Archives of Virology*, vol. 157, no. 4, pp. 791-795, 2012.

[674] M. Redrejo-Rodríguez, D. Munoz-Espín, I. Holguera, M. Mencia, and M. Salas, "Functional eukaryotic nuclear localization signals are widespread in terminal proteins of bacteriophages," *Proceedings of the National Academy of Sciences, USA*, vol. 109, no. 45, pp. 18482-18487, 2012.

[675] I. Rissanen, A. Pawlowski, K. Harlos, J. M. Grimes, D. I. Stuart, and J. K. Bamford, "Crystallization and preliminary crystallographic analysis of the major capsid proteins VP16 and VP17 of bacteriophage P23-77," *Acta Crystallographica Section F, Structural Biology and Crystallization Communications*, vol. 68, no. Pt 5, pp. 580-583, 2012.

[676] F. Rohwer and K. Barott, "Viral information," *Biology and Philosophy*, 2012.

[677] E. Roine and D. H. Bamford, "Lipids of archaeal viruses," *Archaea*, vol. 2012, p. 384919, 2012.

[678] M. J. Roossinck, "Plant virus metagenomics: biodiversity and ecology," *Annual Review of Genetics*, vol. 46, pp. 359-369, 2012.

[679] K. Rosario, S. Duffy, and M. Breitbart, "A field guide to eukaryotic circular single-stranded DNA viruses: insights gained from metagenomics," *Archives of Virology*, vol. 157, no. 10, pp. 1851-1871, 2012.

[680] F. Santos, P. Yarza, V. Parro, I. Meseguer, R. Rossello-Mora, and J. Anton, "Culture-independent approaches for studying viruses from hypersaline environments," *Applied and Environmental Microbiology*, vol. 78, no. 6, pp. 1635-1643, 2012.

[681] C. Schlenker, A. Goel, B. P. Tripet, S. Menon, T. Willi, M. Dlakic, M. J. Young, C. M. Lawrence, and V. Copie, "Structural studies of E73 from a hyperthermophilic archaeal virus identify the "RH3" domain, an elaborated ribbon-helix-helix motif involved in DNA recognition," *Biochemistry*, vol. 51, no. 13, pp. 2899-2910, 2012.

[682] V. Seguritan, N. Alves, Jr., M. Arnoult, A. Raymond, D. Lorimer, A. B. Burgin, Jr., P. Salamon, and A. M. Segall, "Artificial neural networks trained to detect viral and phage structural proteins," *PLoS Computational Biology*, vol. 8, no. 8, p. e1002657, 2012.

[683] A. Sencilo, L. Paulin, S. Kellner, M. Helm, and E. Roine, "Related haloarchaeal pleomorphic viruses contain different genome types," *Nucleic Acids Research*, vol. 40, no. 12, pp. 5523-5534, 2012.

[684] P. S. Shen, M. J. Domek, E. Sanz-Garcia, A. Makaju, R. M. Taylor, R. Hoggan, M. D. Culumber, C. J. Oberg, D. P. Breakwell, J. T. Prince, and D. M. Belnap, "Sequence and structural characterization of great salt lake bacteriophage CW02, a member of the T7-like supergroup," *Journal of Virology*, vol. 86, no. 15, pp. 7907-7917, 2012.

[685] S. Siddaramappa, J. F. Challacombe, R. E. Decastro, F. Pfeiffer, D. E. Sastre, M. I. Gimenez, R. A. Paggi, J. C. Detter, K. W. Davenport, L. A. Goodwin, N. Kyrpides, R. Tapia, S. Pitluck, S. Lucas, T. Woyke, and J. A. Maupin-Furlow, "A comparative genomics perspective on the genetic content of the alkaliphilic haloarchaeon *Natrialba magadii* ATCC 43099T," *BMC Genomics*, vol. 13, p. 165, 2012.

[686] J. C. Snyder, S. K. Brumfield, K. M. Kerchner, T. E. Quax, D. Prangishvili, and M. J. Young, "Insights into a viral lytic pathway from an archaeal virus-host system," *Journal of Virology*, 2012.

[687] J. Sugahara, K. Fujishima, T. Nunoura, Y. Takaki, H. Takami, K. Takai, M. Tomita, and A. Kanai, "Genomic heterogeneity in a natural archaeal population suggests a model of tRNA gene disruption," *PLoS One*, vol. 7, no. 3, p. e32504, 2012.

[688] D. Veesler, J. Quispe, N. Grigorieff, C. S. Potter, B. Carragher, and J. E. Johnson, "Maturation in action: CryoEM study of a viral capsid caught during expansion," *Structure*, vol. 20, no. 8, pp. 1384-1390, 2012.

[689] G. R. Visweswaran, B. W. Dijkstra, and J. Kok, "A genetically engineered protein domain binding to bacterial murein, archaeal pseudomurein, and fungal chitin cell wall material," *Applied Microbiology and Biotechnology*, vol. 96, no. 3, pp. 729-737, 2012.

[690] A. D. Weinberger, C. L. Sun, M. M. Plucinski, V. J. Denef, B. C. Thomas, P. Horvath, R. Barrangou, M. S. Gilmore, W. M. Getz, and J. F. Banfield, "Persisting viral sequences shape microbial CRISPR-based immunity," *PLoS Computational Biology*, vol. 8, no. 4, p. e1002475, 2012.

[691] X. Peng, R. A. Garrett, and Q. She, "Archaeal viruses—novel, diverse and enigmatic," *Science China Life Sciences*, vol. 55, no. 5, pp. 422-433, 2012.

[692] Z. Zhan, S. Ouyang, W. Liang, Z. Zhang, Z. J. Liu, and L. Huang, "Structural and functional characterization of the C-terminal catalytic domain of SSV1 integrase," *Acta Crystallogr D Biol Crystallogr*, vol. 68, no. Pt 6, pp. 659-670, 2012.

[693] X. Zhang, S. Sun, Y. Xiang, J. Wong, T. Klose, D. Raoult, and M. G. Rossmann, "Structure of Sputnik, a virophage, at 3.5-A resolution," *Proceedings of the National Academy of Sciences, USA*, vol. 109, no. 45, pp. 18431-18436, 2012.

[694] Z. Zhang, Y. Liu, S. Wang, D. Yang, Y. Cheng, J. Hu, J. Chen, Y. Mei, P. Shen, D. H. Bamford, and X. Chen, "Temperate membrane-containing halophilic archaeal virus SNJ1 has a circular dsDNA genome identical to that of plasmid pHH205," *Virology*, vol. 434, no. 2, pp. 233-241, 2012.

1. *Correspondence to: Stephen T. Abedon; Department of Microbiology, The Ohio State University, 1680 University Dr., Mansfield, OH 44906 USA; Tel.: 419.755.4343; Fax: 419.755.4327; Email: abedon.1@osu.edu. [↑](#footnote-ref-1)
2. “Yes” if one or more of the following three columns are “Yes”. [↑](#footnote-ref-2)
3. As used to describe a virus or equivalent of domain *Archaea* with these terms either standing alone or in combination with “arch…” [↑](#footnote-ref-3)
4. Or as “Halobacteriophage”. [↑](#footnote-ref-4)
5. As used to describe a virus or equivalent of domain *Archaea*. [↑](#footnote-ref-5)
6. From p. 48: “One additional factor in the north arm water is the presence of a variety of phages specific for the Great Salt Lake bacteria. Two different halophages specific for lake bacteria and five for strains of *Halobacterium halobium*… have been isolated.” [↑](#footnote-ref-6)
7. Uses “halophilic phage” instead. [↑](#footnote-ref-7)
8. “Other likely sources of autolysis include infection by lytic phages, accumulation of toxic products, and excretion of proteases. These mechanisms may occur as well, and a virus-like particle has been observed…” (p. 6). [↑](#footnote-ref-8)
9. “…postulated roles for phage (VLPs) in aquatic environments…” (p. 75). [↑](#footnote-ref-9)
10. “The spontaneous release of VLPs [Virus-Like-Particles] has been reported from archaebacteria…” (p. 78). [↑](#footnote-ref-10)
11. “For routine use, virus was stored in halophage (HF) diluent” (p. 9392) [↑](#footnote-ref-11)
12. Note that this chapter’s title, “Archaeal Phages”, was chosen by the editors rather than the authors (Stedman, personal communication). [↑](#footnote-ref-12)
13. “All but two viruses of Euryarchaeota… are head-and-tail phages…” (p. 1387). [↑](#footnote-ref-13)
14. Probably using “Prophage” generally but difficult to tell from context. [↑](#footnote-ref-14)
15. “In contrast to viruses of extremely thermophilic and hyperthermophilic Archaea, all but two known viruses of extremely halophilic and methane producing Archaea are typical head-and-tail phages” (p. 42) [↑](#footnote-ref-15)
16. Though the following usage may indeed be referring to archaeal viruses, it is our opinion that the intention was not to claim that they are anything other than bacterial viruses (p. 68): “This diversity included particles similar to fuselloviruses, rudiviruses, and lipothrixviruses, as well as typical head-and-tail phages and unusual particles not previously observed in nature.” [↑](#footnote-ref-16)
17. From p. 479: “…some of the phages that infect archaeal hosts have the morphology of tailed phages and share features of genome organization and even sequence with them.” [↑](#footnote-ref-17)
18. “TBLASTN searches revealed a number of φCh1 ORFs of unknown function with similarities to *Hbt. salinarum* virus φH only. This prompted us to investigate the relationship between the two phages in more detail. Unfortunately, only parts of the φH genome have been sequenced…” (p. 856). [↑](#footnote-ref-18)
19. “In contrast to the morphological variety of crenarchaeal viruses, all but two viruses of euryarchaeotes are typical head-and-tail phages…” (p. 2426), although on p. 2419, “Two virus types *resembled* head-and-tail bacteriophages…” (emphasis added). [↑](#footnote-ref-19)
20. From p. 16759: “This is the first case described in archaea, suggesting that targeting of tRNA genes is an ancient process that was conserved during evolution of bacteriophages.” [↑](#footnote-ref-20)
21. “Site-specific integration of SSV1 into its host chromosome is catalyzed by the virus-encoded integrase (Int^SSV^). This enzyme catalyzes recombination between viral and chromosomal attachment sites, *att*P and *att*B (the latter previously denoted *att*A), to generate a left (*att*L) and right (*att*R) prophage *att* sites…” (p. 16758). [↑](#footnote-ref-21)
22. From p. 283: “This VLP population was a mixture of unusual fusiform VLP and tailed phages.” [↑](#footnote-ref-22)
23. To the extent that “viral” is being used broadly enough to include archaeal viruses then “prophage” in this sentence could refer to archaeal proviruses: “The integration of cryptic prophages which have lost their ability to excise and replicate themselves seems to be a common mechanism to acquire viral genes. For example, one of the C-type DNA polymerases of *B. subtilis* is located in a cryptic prophage genome…” (p. 242). [↑](#footnote-ref-23)
24. From p. 1249: “Our defective and unstable viral derivative pMJ02 could become a useful helper phage in a genetic system propagating pSSVx derivatives.” [↑](#footnote-ref-24)
25. “…the vast majority of viruses of euryarchaeotes are typical tailed phages, belonging to the bacteriophage Families *Myoviriadae* and *Syphoviriadae*” (p. 291) [↑](#footnote-ref-25)
26. This article is fairly consistent in distinguishing phages from archaeal viruses, except perhaps for this sentence (p. 61): “Members of the tyrosine recombinases family are found in eukaryotes, and prokaryotes and their phages.” [↑](#footnote-ref-26)
27. “Most viruses known to infect either haloarchaeal or methanogens, of the kingdom Euryarchaeota, are typical head-and-tail phages with icosahedral heads and helical tails” (p. 233). [↑](#footnote-ref-27)
28. “…the tailed phages of Bacteria and Archaea…” (p. 7496). [↑](#footnote-ref-28)
29. Our opinion is that this is *not* a description of archaeal viruses but instead that these archaeal viruses are, as indicated, of a *type* that is equivalent to that of head-and-tail phages: “For the kingdom *Euryarchaeota* most are of the head-and-tail phage type…” (p. 366). [↑](#footnote-ref-29)
30. “Topo V might have been recently transferred from a virus to the archaeal lineage leading to *M. kandleri…*” (p. 246). [↑](#footnote-ref-30)
31. See Table 1 of this publication. [↑](#footnote-ref-31)
32. See their Figure 1 where “Phage” appears to be used as stand in for “Prokaryotic virus”. [↑](#footnote-ref-32)
33. See the row titled “Tailed bacteriophages” in Table 1 of this publication. [↑](#footnote-ref-33)
34. “The covalently bound terminal proteins of the *φ*29 phage family and that of an archaeal phage are ejected from the virion…” (p. 224). [↑](#footnote-ref-34)
35. “Virus” is used extensively in this paper, just not directly in consideration of archaeal viruses. [↑](#footnote-ref-35)
36. “…most viruses of mesophilic and moderately thermophilic bacteria and archaea, which have been characterised show a strong bias to head-tailed phages…” (p. 1203). [↑](#footnote-ref-36)
37. “Methanophage ΨM1” [↑](#footnote-ref-37)
38. “…T4 represents a huge family of viruses that is capable of infecting both Bacteria and Archaea…” (p. 243). [↑](#footnote-ref-38)
39. “’Phages’ or bacteriophages are prokaryote viruses and include viruses of eubacteria and archaea. They occur in vast numbers everywhere in the biosphere,

    especially in the oceans [references]. In addition, most cultivable bacteria harbor complete or defective prophages.” [↑](#footnote-ref-39)
40. See the heading of especially Table 1 of that publication (but also Table 2). [↑](#footnote-ref-40)
41. From p. 1470: “Some of these viruses are temperate phages that can lysogenize the host to become prophages.” [↑](#footnote-ref-41)
42. From p. 1709: “Clustered regularly interspaced short palindromic repeats (CRISPR) are a distinctive feature of the genomes of most Bacteria and Archaea and are thought to be involved in resistance to bacteriophages.” [↑](#footnote-ref-42)
43. “CRISPR Provides Acquired Resistance Against Viruses in Prokaryotes” (article title). [↑](#footnote-ref-43)
44. From p. 239: “Probably haloviral p.” [↑](#footnote-ref-44)
45. “…methanophage ψM2…” (p. 4517) [↑](#footnote-ref-45)
46. From p. 247: “Prophinder was run over 404 bacterial and archaeal genomes, generating around 550 prophage predictions distributed over 200 genomes.” This would be a “Yes” for “Prophage” if “Prophages” were indeed identified in archaeal genomes. [↑](#footnote-ref-46)
47. The descriptor “Prophage-like” is used generally, but not explicitly to describe the *Methanococcus voltae* gene transfer agent. [↑](#footnote-ref-47)
48. Though a clear distinction is made between “bacteriophages and archaeviruses” (p. 567), nevertheless the term “Phage” seems to be used generally throughout the article to imply both. [↑](#footnote-ref-48)
49. “…bacteria, archaea, and their viruses…” (p. 1027). [↑](#footnote-ref-49)
50. “The resurgence of interest in prokaryotic virus (phage) biology…” (p. 311). [↑](#footnote-ref-50)
51. From p. 1187: “…most of the viruses are prokaryote-infecting viruses also known as phages or bacteriophages.” [↑](#footnote-ref-51)
52. “The number of lysogenic prokaryotes (LP) is generally estimated by dividing the number of viruses produced, due to prophage induction…” (p. 1192). [↑](#footnote-ref-52)
53. Though a clear distinction is made between “bacterial and archael viruses” (p. 447), nevertheless the term “Phage” seems to be used generally to imply both [↑](#footnote-ref-53)
54. “*Acidianus* bottle-shaped virus” (p. 1126). [↑](#footnote-ref-54)
55. The phrase, “it is not currently possible to tell with certainty whether any of these prophages is inducible” (p. 299), appears to refer at least in part to the archaeal proviruses analyzed in this study. [↑](#footnote-ref-55)
56. “Horizontal gene transfer (HGT) in bacteria and archaea occurs through phage transduction, transformation, and conjugation…”, where “Phage” probably is intended to describe both bacterial and archaeal processes (p. 1983). [↑](#footnote-ref-56)
57. “…previous studies that show integrase homologs in six crenarchaeal viral genomes… and induction of prophage by mitomycin C in 1 to 9% of hot-spring microbial cells…” (p. 4167). [↑](#footnote-ref-57)
58. We interpret the following as describing viruses in combination with bacteriophages as constituting an all-inclusive whole rather than necessarily fully equating the two concepts (p. 7): “The dataset was then partitioned into four subsets categorized by molecules belonging to each of the three superkingdoms or viruses/bacteriophages.” [↑](#footnote-ref-58)
59. “Tailed phages infect Eubacteria and Archaea…” (p. 131). [↑](#footnote-ref-59)
60. “Recent recovery of genomic sequences from bacteriophage and archaeal viruses from AMD biofilms also indicated a low viral diversity…” (p. 2198). [↑](#footnote-ref-60)
61. It is difficult to parse whether “Phage” is being applied to viruses of bacteria or instead viruses of both *Bacteria* and *Archaea* (though we assume the former): “…viruses of archaea and bacteria (phage)…” (p. 1198). [↑](#footnote-ref-61)
62. From p. 408: “…acquired resistance mechanism in prokaryotes against phages and/or plasmids.” [↑](#footnote-ref-62)
63. “More recently, the same authors found that only 18% of viral ORFans (ORFs present in only one viral genome) have homologues in archaeal or bacterial genomes, and concluded that 'phage ORFans play a lesser role in horizontal gene transfer to prokaryotes'…” (p. 2). [↑](#footnote-ref-63)
64. From p. 6: “…distinct genomic blocks of up to 24 genes with atypical %GC content inferred to be the result of prophage insertion...” [↑](#footnote-ref-64)
65. There is some consideration of phages at the end of p. 133 and beginning of p. 135, but it is difficult to tie this discussion directly to consideration of archaeal viruses. [↑](#footnote-ref-65)
66. We assume that numerous instances of “Phage” in Table 1 of this publication all refer to bacterial viruses, as “Archaeal virus” otherwise is clearly labeled. [↑](#footnote-ref-66)
67. It is our opinion that “Phage” “Prophage” are used as synonyms for “Prokaryotic virus” and therefore to describe archaeal as well as bacterial viruses. [↑](#footnote-ref-67)
68. “…those present as prophages in the 736 completely sequenced prokaryotic genomes…” (p. 168). [↑](#footnote-ref-68)
69. It is our opinion that “Phage” is being used as a synonym for “Prokaryotic virus” in this publication, such as in the sentence (p. 208), “Viromes are good hunting grounds for unique host-adaptation genes, as shown in a recent metagenomic study of phage from deep-sea hydrothermal vents.” [↑](#footnote-ref-69)
70. We feel that this usage is consistent with “Phage” not being employed to describe archaeal viruses (from p. 2771): “Currently, it is thought that most of the viruses are phages that infect bacteria, but archaeal and eukaryotic viruses are certainly important components of most ecosystems.” [↑](#footnote-ref-70)
71. “…halophilic Bacteria and Archaea have been used to isolate halophages to better understand the ecology of haloviruses…” (p. 633). [↑](#footnote-ref-71)
72. From p. 735: “…viruses of eukaryotes inherited genetic structures from phages and viruses of archaeans.” [↑](#footnote-ref-72)
73. From p. 1139: “a… phage terminase could also be identified.” [↑](#footnote-ref-73)
74. “…phages (i.e. viruses that infect prokaryotes)…” (p. 98). [↑](#footnote-ref-74)
75. The phrase “phage integrase” is used in various places rather than “phage-like integrase” or the equivalent. [↑](#footnote-ref-75)
76. From p. 9: “…88 out of 4500 spacers from a broad range of bacteria and archaea matched to known sequences, with most being similar to bacteriophage and plasmids… Remarkably, species containing identified spacer elements were immune to the corresponding foreign invaders or had no prophage remnants as evidence of prior infections.” [↑](#footnote-ref-76)
77. “…only a handful of RNA viruses that infect bacteria and none are known to infect archaea… In line with this distribution, no RNA phages have been found so far in the marine RNA viromes…” (p. 17). [↑](#footnote-ref-77)
78. From p. 1: “…we have proposed that viruses can be grouped into lineages regardless of which domain of life (bacteria, archaea, eukarya) they infect.” [↑](#footnote-ref-78)
79. “…bacterial and archaeal genomes… evolve rapidly, acquiring new spacer sequences to adapt to highly dynamic viral populations…” (p. 568). [↑](#footnote-ref-79)
80. From p. 894: “The other eight genes have homologues in viruses whose hosts are from all three domains of life, the *Eukarya*, *Archaea*, and *Bacteria*.” [↑](#footnote-ref-80)
81. “…persisting prophage of prokaryotes… (p. 704). [↑](#footnote-ref-81)
82. “…the T4-like viruses (tailed icosahedral phage, Caudavirales) represent a huge family of viruses that is capable of infecting both bacteria and archaea…” (p. 703). [↑](#footnote-ref-82)
83. From p. 18431: “…Spunik’s genome have similarity to eukaryal, archaeal, and bacterial virus genes.” [↑](#footnote-ref-83)
84. “The in situ assembly of the *Sulfolobus* turreted icosahedral virus (STIV) which infects the archaea *Sulfolobus solfataricus*… STIV belongs to the PRD1-Adeno lineage of viruses…” (p. 494). [↑](#footnote-ref-84)
85. “…archaeal interactions with other microorganisms (including viruses and phages) and their hosts…” (p. 52). [↑](#footnote-ref-85)
86. This is based on the use of “other” here: “Broad degradative function has been observed in the metabolism of other bacteriophages…” (p. 623) [↑](#footnote-ref-86)
87. “CRISPR… system in bacteria and archaea is a small RNA-based defence mechanism against phages and plasmids…” (p. 926). [↑](#footnote-ref-87)
88. “As of August 2008, data for 727 completely sequenced microbial genomes were available, of which 351 were found to contain no integrated prophages…” (p. 1807). [↑](#footnote-ref-88)
89. From p. 6044: “Factors defining the abundance of phage defense and mobilome genes in bacterial and archaeal genomes.” [↑](#footnote-ref-89)
90. “…bacteria and archaea are subject to a constant barrage by diverse viruses… (p. 6039). [↑](#footnote-ref-90)
91. “Other halophilic viruses with similarities to the metagenome included Virus PhiCh1 (*E*-values = 1e-6, 2e-18 and 8e-13), the His1 virus (*E*-values = 9e-22 and 1e-5), bacteriophage phi-H (*E*-value = 1e-36) and various environmental halophages…” (p. 1962). [↑](#footnote-ref-91)
92. “…various environmental halophages…” (p. 1962). [↑](#footnote-ref-92)
93. “Phages found in the hot water environment also show large diversity… For example, thermophilic archaeal viruses display an exceptional degree of diversity with regard to both morphotype and genome…” (p. 152) [↑](#footnote-ref-93)
94. From p. 321: “…viruses and plasmids in archaea and bacteria.” [↑](#footnote-ref-94)
95. See heading, “Tailed phages”, in Table 2. [↑](#footnote-ref-95)
96. “Most archaeal viruses and halophages described here…” (p. 435) appears to use “halophage” as a description of bacterial viruses. [↑](#footnote-ref-96)
97. “It is important to consider that viral integration into archaeal genomes is a common occurrence…” (p. 3194). [↑](#footnote-ref-97)
98. “…notwithstanding the inclusion of archaeal viruses, we keep the acronym POGs for continuity and convenience” (from preprint). [↑](#footnote-ref-98)
99. “Tailed phages are uncommon in archaea...” (p. 477). [↑](#footnote-ref-99)
100. “…the CRISPR/Cas system seems not only limited to phage defense but also to play a more general role in the prevention of horizontal gene transfer and the maintenance of genetic integrity in *Bacteria* and *Archaea*…” (p. 2491). [↑](#footnote-ref-100)
101. This assessment is based on this (p. 1639) “…previously isolated halophages. This low GC content, together with the codon usage, allowed us to propose *Hqt. walsbyi* as the host.” Nonetheless, note the following from the same page: “The analysis of this genome (named environmental halophage-1 [EHP-1], although if this virus is infecting an archaeon this name may not be appropriate…” [↑](#footnote-ref-101)
102. “Euryarchaeal viruses are most frequently head-and-tail phages…” (p. 2899). [↑](#footnote-ref-102)
103. “Viruses, largely prokaryotic viruses (bacteriophages or phages) are the most abundant carrier of genetic material in marine environments…” (p. 1). [↑](#footnote-ref-103)
104. “…two archaea-specific viral hydrolases…” (p. 730). [↑](#footnote-ref-104)
105. Minimally, this entails use of the term, “Provirus”. [↑](#footnote-ref-105)
106. In some insteances more complex phrases have been simplified such as “Crenarchaeal hyperthermophilic viruses” which has been simplified to “Crenarcheal virus” or “Archaeal halophilic virus” which is listed simply as “Archaeal virus”. [↑](#footnote-ref-106)
107. Including euryarchaeal head-and-tail phage. [↑](#footnote-ref-107)
